# Bioinformatics analysis across pan-cancer and experimental validation in hepatocellular carcinoma revealed the oncogenic role of SF3B6

**DOI:** 10.3389/fphar.2025.1516534

**Published:** 2025-04-28

**Authors:** Linshuang Tu, Jiefu Luo, Ya Yin, Huihong Yu

**Affiliations:** ^1^ Department of Gastroenterology, The Second Affiliated Hospital of Chongqing Medical University, Chongqing, China; ^2^ Department of Hepatobiliary Surgery, The Second Affiliated Hospital of Chongqing Medical University, Chongqing, China

**Keywords:** SF3B6, pan-cancer, hepatocellular carcinoma (HCC), biomarker, alternative splicing

## Abstract

**Background:**

Splicing factor 3b subunit 6(SF3B6), a subunit of the SF3B complex, regulates the process of RNA splicing by recognizing the branch point adenosine in pre-mRNA and facilitating the interaction between U2 snRNA and the branch point sequence. Currently, there is no systematic multi-omics study exploring the diagnostic, prognostic, and immunotherapy predictive value of SF3B6 in pan-cancer, nor is its role in hepatocellular carcinoma (HCC) clear.

**Methods:**

We utilized various databases to systematically examine the expression and genetic variation of SF3B6 across multiple cancer types, assessing its relationship with diagnosis, prognosis, immune infiltration, immunotherapy response, and associated signaling pathways. Additionally, we investigated the correlation between SF3B6 and prognosis, clinicopathological features, and treatment responses in HCC, as well as the roles of its related alternative splicing isoforms. Finally, we conducted *in vitro* experiments to validate the effects of SF3B6 on the proliferation, migration, invasion, apoptosis, and cell cycle progression of liver cancer cells.

**Results:**

Results indicate that SF3B6 was highly expressed in various cancers and regulated by copy number variations and DNA methylation. The elevated expression of SF3B6 demonstrated predictive value for cancer diagnosis, prognosis, and responses to immunotherapy. Functional enrichment analysis suggests that SF3B6 was closely associated with pathways related to tumor immunity, tumor metabolism, and cell cycle. Additionally, high SF3B6 expression was an independent risk factor for overall survival and correlated with poor alpha-fetoprotein levels, pathological grading, clinical staging, and reduced responses to sorafenib and transarterial chemoembolization treatment in HCC. Interestingly, SF3B6 was associated with variant splicing isotypes of genes involved in the G2M checkpoint and DNA repair pathways, including NEIL3, NEK2, KIF4A, TROAP, and FANCD2. Moreover, SF3B6 was highly expressed in liver cancer cells, promoting the proliferation, migration, and invasion of cancer cells, inhibiting apoptosis, and regulating the transition from the S phase to the G2M phase of the cell cycle.

**Conclusion:**

We emphasize that SF3B6 has the potential to serve as a biomarker for predicting cancer diagnosis, prognosis, and immunotherapy responses, especially in HCC. SF3B6 and its related alternative splicing isoforms promote the occurrence and progression of HCC and may serve as potential therapeutic targets.

## 1 Introduction

Cancer represents a critical global health issue that poses a substantial threat to public health across the globe (Sung et al., 2021). GLOBOCAN data reveals that in 2020, there were 19.3 million new cancer cases worldwide, resulting in nearly 10 million fatalities (Sung et al., 2021). By 2040, the incidence of new cancer cases is projected to increase by 47% (Sung et al., 2021). In 2020, primary liver cancer was the sixth most prevalent cancer and the third leading cause of cancer-associated mortality worldwide (Llovet et al., 2021b). Hepatocellular carcinoma (HCC), which makes up 75%–85% of these cases, is the predominant type of primary liver cancer (Llovet et al., 2021b). Treatment options for patients vary depending on their stage of cancer (Brown et al., 2023). Surgical and local therapies, such as transarterial chemoembolization, are the primary treatment options for early and intermediate-stage HCC patients, while systemic therapies, primarily consisting of targeted therapies and immune checkpoint inhibitors, provide survival benefits for advanced HCC (Llovet et al., 2021a; Yang et al., 2023). Despite advancements in medical technology, the prognosis for cancer patients remains unfavorable due to limited drug efficacy, side effects, and resistance.With the advancements in high-throughput sequencing and bioinformatics, numerous studies have utilized transcriptomic data from authoritative databases such as TCGA and GEO to investigate the molecular mechanisms and resistance pathways of HCC (Gao et al., 2024; Yuan et al., 2024). These studies have identified potential biomarkers that provide a more comprehensive understanding of the intrinsic transcriptional characteristics and genetic heterogeneity of HCC, thereby advancing personalized diagnostic and therapeutic strategies (Gao et al., 2024; Yuan et al., 2024).

Alternative splicing (AS) is the process of selectively removing introns and joining exons from the same precursor mRNA to generate different mature RNA splice variants (Wilkinson et al., 2020). This process is primarily executed by the spliceosome and other trans-acting splicing factors that modulate its activity (Wilkinson et al., 2020). Dysregulation of alternative splicing—resulting from mutations or expression changes in key splicing factors, trans-acting splicing regulators, and mutations in cis-acting sequences—is prevalent in various cancers and facilitates tumor development (Bradley and Anczuków, 2023). Small-molecule inhibitors, siRNAs, and splice-switching oligonucleotides (ASOs) aimed at AS drivers, splicing factors, and pathological AS hold great therapeutic potential (Rogalska et al., 2023).

During mRNA splicing, the U2-type spliceosome comprises U1, U2, U4, and U6 small nuclear ribonucleoproteins (snRNPs), which specifically recognize GT-AG splice sites and facilitate the removal of approximately 99% of introns (Wilkinson et al., 2020). The Splicing Factor 3b (SF3B) complex, a critical component of the U2 snRNP, consists of seven subunits: SF3B1, SF3B2, SF3B3, SF3B4, SF3B5, SF3B6, and PHF5A (Du et al., 2021). These subunits play key roles in recognizing branch point sequences (BPS) and facilitating the assembly and activation of the spliceosome (Sun, 2020). Notably, SF3B1 and PHF5A participate in regulating various biological events, including maintaining embryonic stem cell pluripotency, chromatin remodeling, DNA damage repair, and cell growth and differentiation (Li et al., 2023; Samy et al., 2023). They achieve this by modulating the aberrant selective splicing of target genes or through their non-spliceosome-related functions, thus promoting tumorigenesis. Inhibitors targeting the SF3B complex or its SF3B1 subunit can precisely calibrate the constitutive or selective splicing of precursor mRNA by altering the ability of SF3B1 and PHF5A to recognize different intron branch point sequences, thereby exerting anti-tumor activity (Larsen, 2021; Yamauchi et al., 2022).

Previous studies have demonstrated that the SF3B complex recognizes the branch site (BS) of splice-supporting sequences in pre-mRNA through Splicing factor 3b subunit 6(SF3B6), thereby facilitating and maintaining the interaction between U2 snRNA and these sequences (Lee et al., 2020; Sun, 2020). Despite being an important subunit of the SF3B complex, SF3B6 has relatively few functional studies in tumors. Research has indicated that phosphorylated SF3B6 affects alternative splicing, inducing a carcinogenic phenotype in pancreatic ductal adenocarcinoma (PDAC) (Li J. et al., 2021). In breast cancer, upregulation of SF3B6 expression is related to worse prognosis (Peng et al., 2022). Additionally, silencing SF3B6 has been shown to increase p53 levels, resulting in cell cycle arrest and apoptosis in non-small cell lung cancer (NSCLC) (Siebring-van Olst et al., 2017). However, comprehensive multi-omics analyses on the role of SF3B6 in diagnosis, prognosis, immune infiltration, and responses to immunotherapy across various cancers are lacking. Furthermore, the role of SF3B6 in HCC remains inadequately defined.

This research aims to conduct a thorough analysis of SF3B6 expression and genetic variation across various cancers using multi-omics data. It will evaluate the correlation of SF3B6 with cancer diagnosis, prognosis, immune infiltration, immune therapy response, and signaling pathways. The study will further explore the clinical value and function of SF3B6 and its related alternative splicing events in HCC, validating the biological function of SF3B6 on HCC cells using *in vitro* experiments. The goal is to clarify its viability as a biomarker for predicting cancer diagnosis, prognosis, and immune therapy, while also establishing its potential as a therapeutic target. This research will provide a foundational experimental basis and innovative ideas for future studies on SF3B6 in HCC.

## 2 Results

### 2.1 Differential expression of SF3B6 in pan-cancer

**FIGURE 1:**
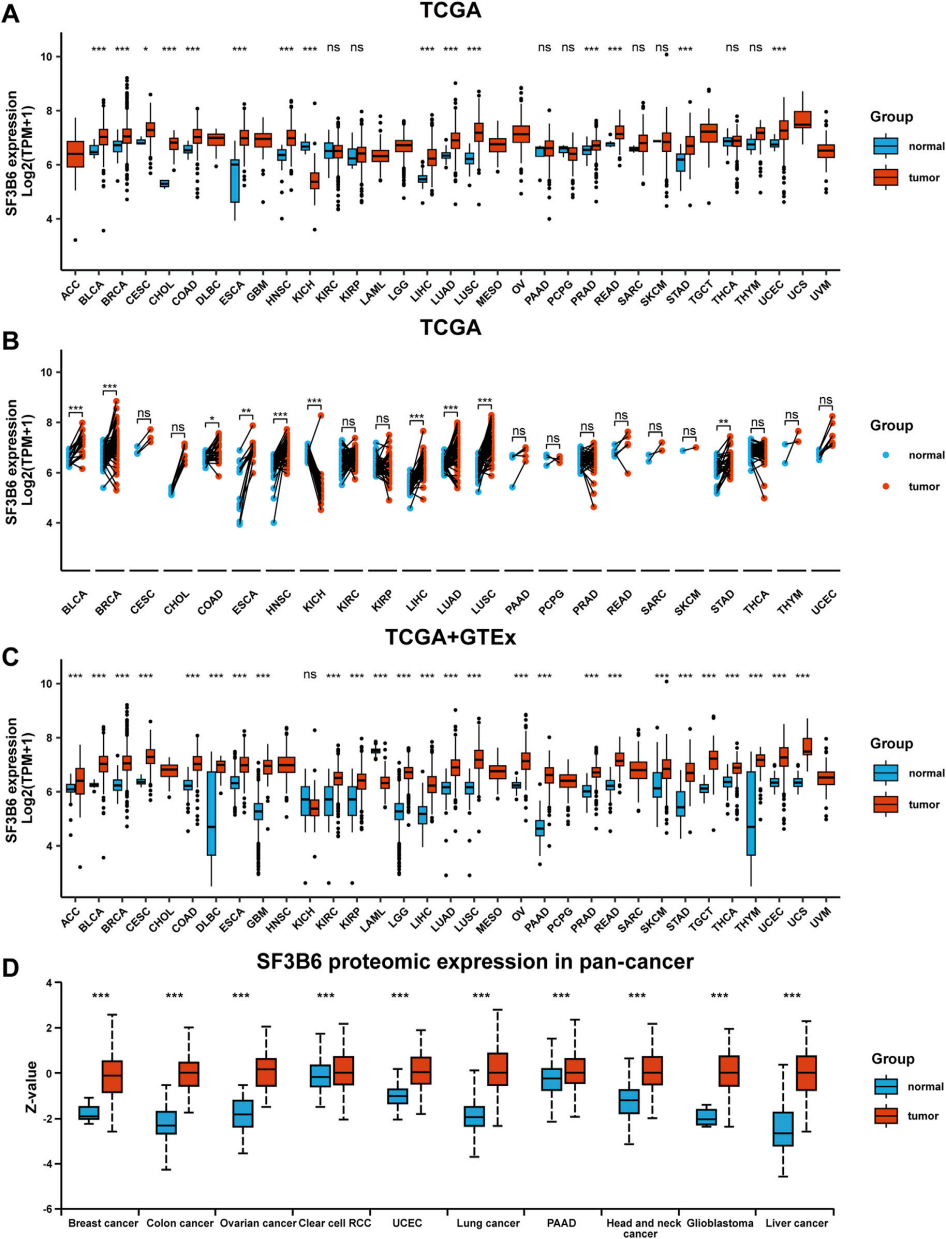
Differential expression analysis of SF3B6 between normal and tumor tissues in pan-cancer. **(A)** Unpaired analysis of SF3B6 mRNA expression between normal and tumor tissues based on TCGA database. **(B)** Paired analysis of SF3B6 mRNA expression between normal and tumor tissues based on TCGA database. **(C)** SF3B6 mRNA expression level between normal and tumor tissues based on TCGA and GTEx database. **(D)** SF3B6 protein expression level between normal and tumor tissues based on CPTAC databases. TCGA, The Cancer Genome Atlas Program; GTEx, Genotype-Tissue Expression; CPTAC, Clinical Proteomic Tumor Analysis Consortium. *p < 0.05, **p < 0.01, ***p < 0.001. ns not significant.

We initially obtained gene expression data for SF3B6 from the TCGA database and conducted unpaired differential analysis between tumor tissues and adjacent non-tumor tissues. The abbreviations of 33 cancer types in TCGA are listed in Supplementary Table S1. The results indicated that SF3B6 gene expression was significantly upregulated in 14 cancer types (BLCA, BRCA, CESC, CHOL, COAD, ESCA, HNSC, LIHC, LUAD, LUSC, PRAD, READ, STAD, UCEC), but downregulated in KICH (Figure 1A). Paired differential analysis of tumor and adjacent samples further revealed that SF3B6 expression was significantly upregulated in tumor tissues of BLCA, BRCA, COAD, ESCA, HNSC, LIHC, LUAD, LUSC, and STAD, but downregulated in KICH tumor tissues (Figure 1B). Due to the unavailability of adjacent samples for certain tumor types, we obtained SF3B6 gene expression data from both the TCGA and GTEx databases to perform unpaired differential analysis between tumor tissues and normal tissues. The results showed that SF3B6 gene expression was significantly upregulated in 24 cancer types (ACC, BLCA, BRCA, CESC, COAD, DLBC, ESCA, GBM, KIRC, KIRP, LGG, LIHC, LUAD, LUSC, OV, PAAD, PRAD, READ, SKCM, STAD, TGCT, THYM, UCEC, UCS), but downregulated in KICH and LAML (Figure 1C). Additionally, we retrieved protein expression data for SF3B6 from the UALCAN database, which indicated a significant increase in SF3B6 protein levels in liver cancer, glioblastoma, head and neck cancer, lung cancer, ovarian cancer, colon cancer, and breast cancer compared to normal tissues (Figure 1D).

### 2.2 Genetic variation analysis of SF3B6 in pan-cancer

**FIGURE 2:**
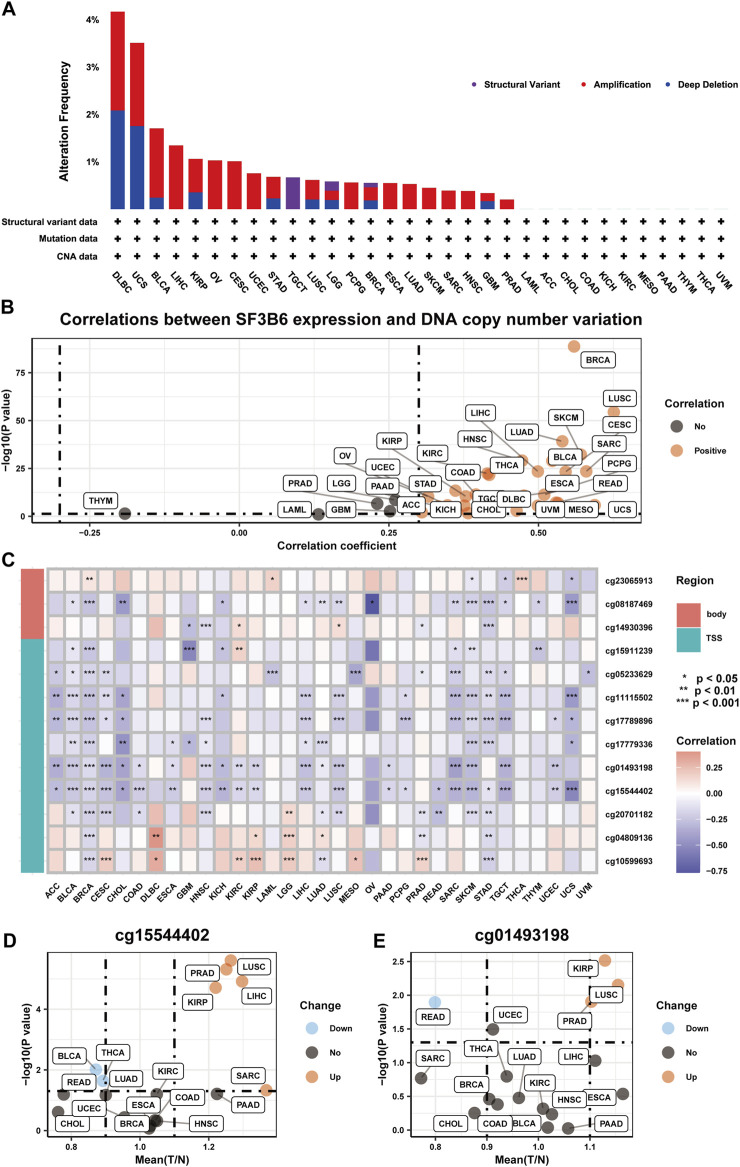
The genetic alterations of SF3B6 in pan-cancer. **(A)** Summary of SF3B6 genetic alteration in TCGA PanCancer Atlas Studies. **(B)** Scatter plot illustrating the results of Spearman correlation analysis between mRNA expression and DNA copy number variation of SF3B6 based on TCGA and GTEx database. **(C)** Heatmap displaying the results of Spearman correlation analysis between mRNA expression and methylation levels of multiple sites of SF3B6 based on TCGA and GTEx database. Scatter plots showing the results of Differential expression analysis of **(D)** cg15544402 and **(E)** cg01493198 between normal and tumor tissues based on TCGA and GTEx database. *p < 0.05, **p < 0.01, ***p < 0.001. ns not significant.

To investigate the underlying mechanisms for the increased expression of SF3B6, we explored the genetic variations and DNA methylation status of SF3B6 using the TCGA dataset. Analysis via the cBioPortal database revealed that amplification, deep deletion, and structural variants were the primary types of genetic alterations affecting SF3B6. Among these, DLBC exhibited the highest mutation frequency at 4.17%, with amplification and deep deletion noted at 2.08% (Figure 2A). Given that amplification and deep deletion represent copy number variations (CNVs), we further explored the correlation between SF3B6 expression and CNVs across different cancer types. The results suggested a positive association between SF3B6 expression and CNVs in most cancer types (Figure 2B), which may partially explain the elevated expression of SF3B6 observed in these cancers.

DNA methylation is a crucial epigenetic regulatory mechanism that can modulate gene expression levels without altering the DNA sequence (Meng et al., 2015; Mattei et al., 2022). High levels of methylation in DNA promoter regions are often associated with gene silencing and downregulation. Thus, we conducted further analysis to assess the association between SF3B6 expression and DNA methylation levels. Our findings indicated a positive association between SF3B6 gene expression and methylation levels at various sites in the DNA promoter region across 11 cancer types, suggesting that increased SF3B6 expression may be regulated by DNA methylation (Figure 2C). Notably, the methylation levels of cg15544402 and cg01493198 were negatively associated with SF3B6 gene expression in more than half of the cancer types. Furthermore, in PRAD, LUSC, KIRP, LIHC, and SARC, the methylation levels of cg15544402 were significantly increased (Figure 2D). Similarly, In KIRP, LUSC, and PRAD, the methylation levels of cg01493198 were also significantly elevated (Figure 2E).

### 2.3 Diagnostic value of SF3B6 in pan-cancer

**FIGURE 3:**
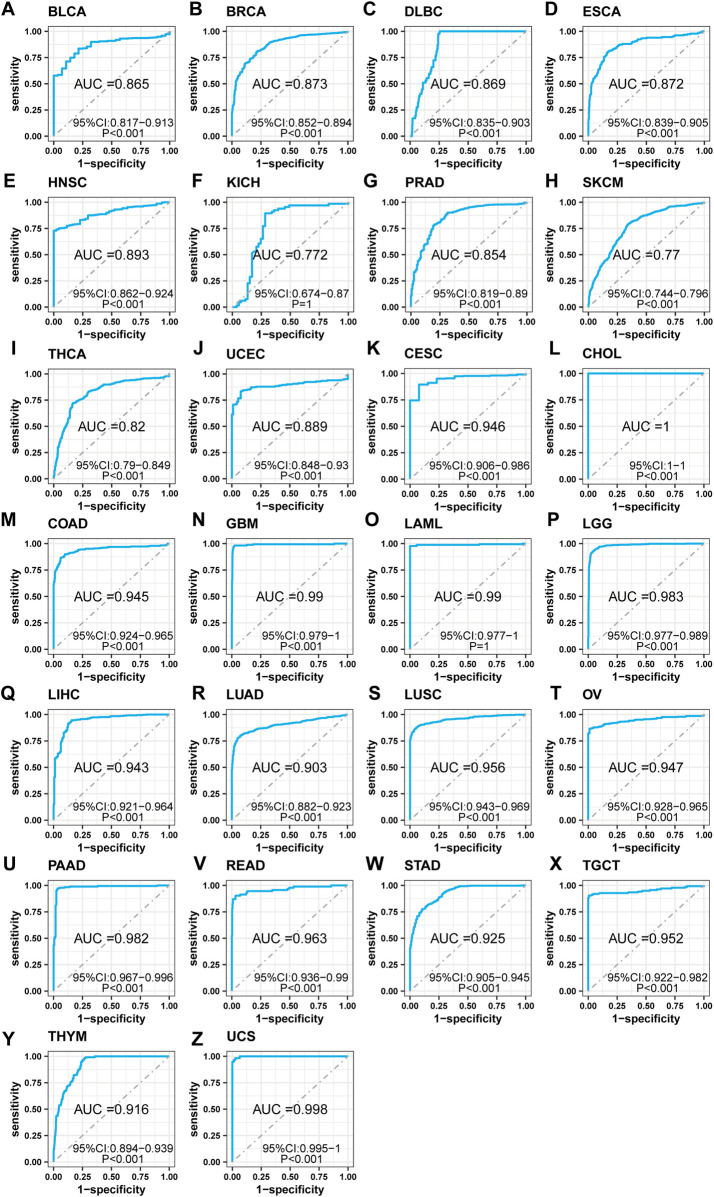
Receiver operator characteristic (ROC) analysis of SF3B6 in pan-cancer. **(A)** BLCA. **(B)** BRCA. **(C)** DLBC. **(D)** ESCA. **(E)** HNSC. **(F)** KICH. **(G)** PRAD. **(H)** SKCM. **(I)** THCA. **(J)** UCEC. **(K)** CESC. **(L)** CHOL. **(M)** COAD. **(N)** GBM. **(O)** LAML. **(P)** LGG. **(Q)** LIHC. **(R)** LUAD. **(S)** LUSC. **(T)** OV. **(U)** PAAD. **(V)** READ. **(W)** STAD. **(X)** TGCT. **(Y)** THYM. **(Z)** UCS.

To investigate the clinical relevance of SF3B6, we conducted a receiver operator characteristic (ROC) analysis to assess its diagnostic potential across various cancer types. The analysis demonstrated that SF3B6 exhibited good diagnostic value in 26 tumor types (AUC >0.7). Specifically, it showed strong diagnostic capability in the following cancer types, with AUC values ranging from 0.7 to 0.9: BLCA, BRCA, DLBC, ESCA, HNSC, KICH, PRAD, SKCM, THCA, and UCEC (Figures 3A-J). Furthermore, SF3B6 exhibited excellent diagnostic capability in the following cancers, with AUC values exceeding 0.9: CESC, CHOL, COAD, GBM, LAML, LGG, LIHC, LUAD, LUSC, OV, PAAD, READ, STAD, TGCT, THYM, and UCS (Figures 3K-Z).

### 2.4 Prognostic value of SF3B6 in pan-cancer

**FIGURE 4:**
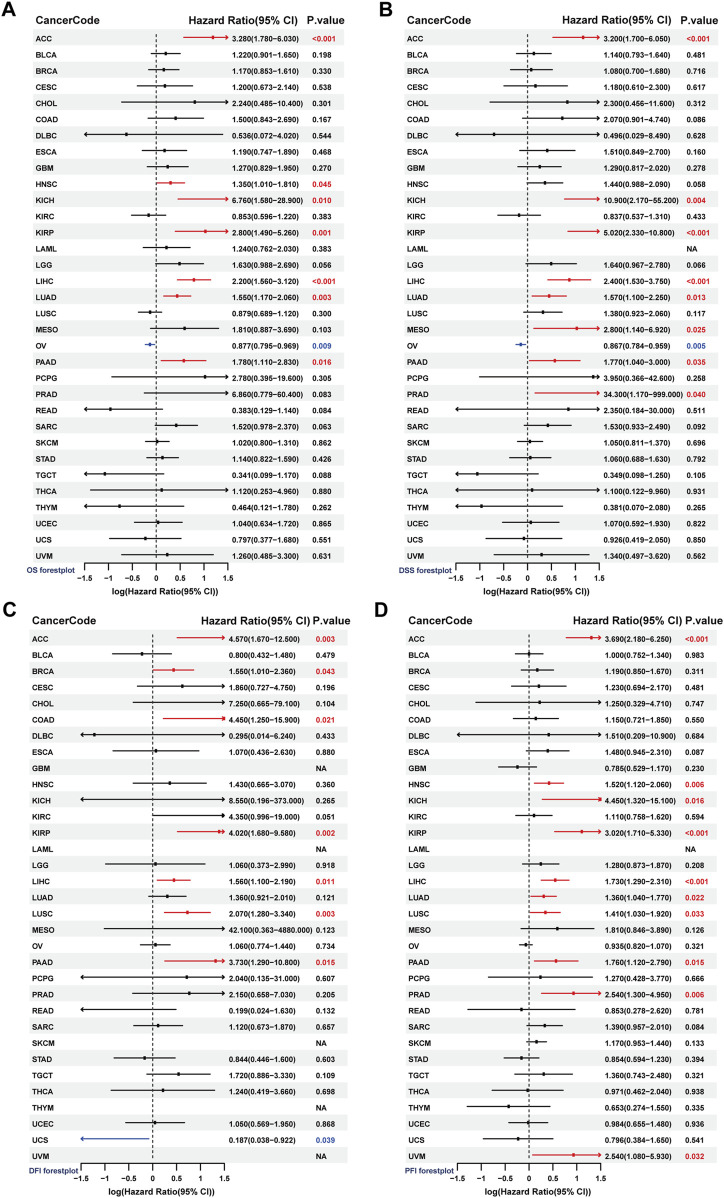
Analysis of the prognostic value of SF3B6 in pan-cancer. Forest plots show the results of univariate COX regression analysis of SF3B6 expression in pan-cancer for **(A)** OS, **(B)** DSS, **(C)** DFI, and **(D)** PFI. OS, Overall Survival. DSS, Disease Specific Survival. DFI, Disease Free.

We conducted univariate Cox regression analysis to assess the prognostic predictive value of SF3B6 for overall survival (OS), progression-free interval (PFI), disease-free interval (DFI), and disease-specific survival (DSS). The analysis indicated that elevated SF3B6 expression was associated with shorter OS in several cancers, including ACC, HNSC, KIRP, LIHC, LUAD, and PAAD. Conversely, low SF3B6 expression was linked to shorter OS in ovarian cancer (OV) (P < 0.05) (Figure 4A). Additionally, elevated SF3B6 expression was correlated with shorter DSS in ACC, KICH, KIRP, LIHC, LUAD, MESO, PAAD, and PRAD, while low expression in OV was associated with shorter DSS (P < 0.05) (Figure 4B). Elevated SF3B6 expression consistently correlated with shorter DSS in ACC, KICH, KIRP, LIHC, LUAD, MESO, PAAD, and PRAD, while lower expression was linked to shorter DFI in OV (P < 0.05) (Figure 4C). Furthermore, elevated SF3B6 expression was associated with shorter PFI in ACC, HNSC, KICH, KIRP, LIHC, LUAD, LUSC, PAAD, PRAD, and UVM (P < 0.05) (Figure 4D).

### 2.5 Immune infiltration analysis of SF3B6 in pan-cancer

**FIGURE 5:**
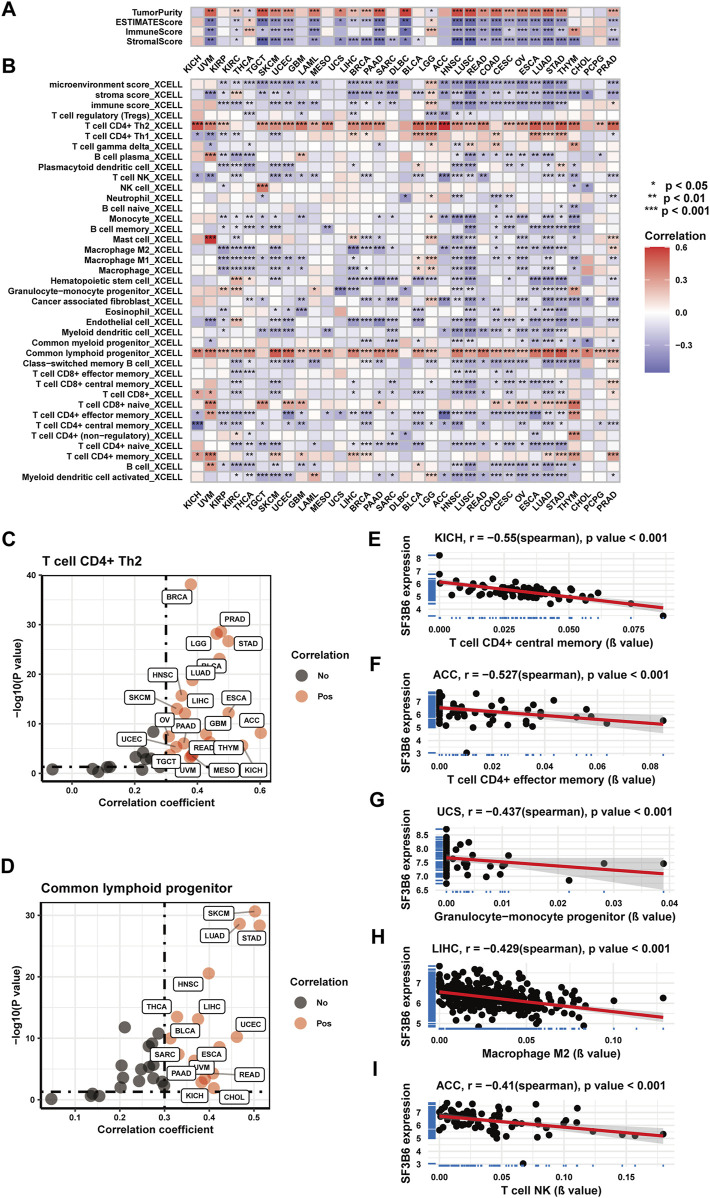
Correlation analysis between and with immune infiltration in pan-cancer. **(A)** Heatmap showing the correlation between SF3B6 and Immune infiltration in pan-cancer based on the ESTIMATE algorithm. **(B)** Heatmap showing the correlation between SF3B6 and various immune cell infiltration in pan-cancer utilizing the XCELL algorithm. **(C)** Scatter plot showing the correlation between SF3B6 and type 2 helper CD4 + T cells in pan-cancer. **(D)** Scatter plot showing the correlation between SF3B6 and common lymphoid progenitors in pan-cancer. **(E-I)** Scatter plots showing the top 5 strongest negative correlations between SF3B6 and immune cell infiltration utilizing the XCELL algorithm.

We initially examined the correlation between SF3B6 gene expression and the infiltration of immune and stromal cells across various cancer types utilizing the ESTIMATE algorithm. The findings revealed that in the majority of cancers, SF3B6 gene expression exhibited a negative relationship with both immune scores and stromal scores while showing a positive relationship with tumor purity scores (Figure 5A). Next, we analyzed the association of SF3B6 expression with various cell infiltrations in the tumor microenvironment by applying the XCELL algorithm. The results were consistent with those obtained from the ESTIMATE algorithm, indicating that SF3B6 gene expression was negatively linked to tumor microenvironment scores, stromal scores, and immune scores in most cancer types (Figure 5B). Additionally, SF3B6 was positively related to CD4^+^ T helper (Th) two cells and common lymphoid progenitors in most cancer types, while it demonstrated a negative association with the infiltration of other immune cell types (Figures 5C,D). Specifically, in ACC, SF3B6 showed a negative relationship with effector memory CD4 T cells (R = −0.527) and natural killer T cells (R = −0.41) (Figures 5E,I). Moreover, negative correlations were observed with central memory CD4 T cells (R = −0.55) in KICH (Figure 5F), granulocyte-monocyte progenitors (R = −0.437) in UCS (Figure 5G), and M2 macrophages (R = −0.429) in LIHC (Figure 5H).

### 2.6 Immunotherapy predictive value of SF3B6 in pan-cancer

**FIGURE 6:**
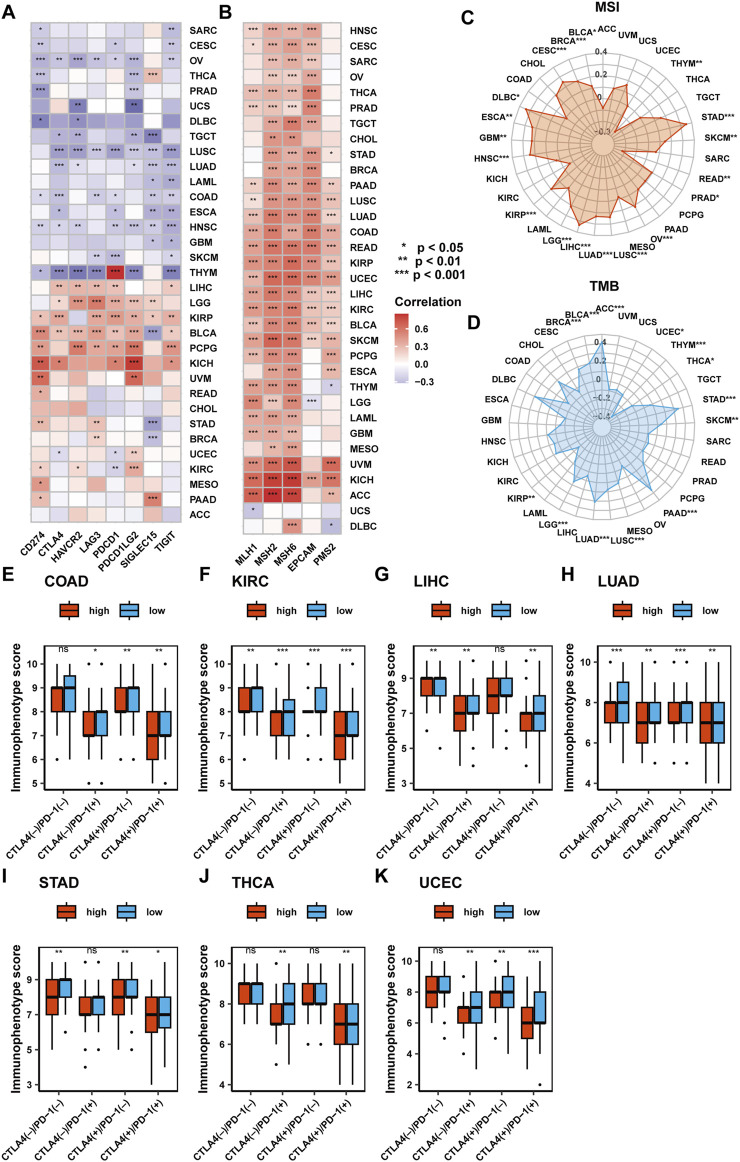
Analysis of the predictive value of SF3B6 in response to immunotherapy in pan-cancer. **(A)** Heatmap showing the correlation of SF3B6 with eight immune checkpoints. **(B)** Heatmap showing the correlation between SF3B6 and the six MMR genes. **(C)** Radar plot showing the correlation between SF3B6 and tumor mutation burden. **(D)** Radar diagram showing the correlation between SF3B6 and microsatellite instability. Differences in immunophenotype scores in **(E)** COAD, **(F)** KIRC, **(G)** LIHC, **(H)** LUAD, **(I)** STAD, **(J)** THCA, **(K)** UCEC.

Considering the association of SF3B6 with the tumor microenvironment and immune cell infiltration across various cancer types, we executed a further analysis of the predictive value of SF3B6 in immunotherapy. Previous studies have identified several biomarkers, such as mutation burden (TMB), microsatellite instability (MSI), mismatch repair system (MMR)-related genes, and immune checkpoint molecules, that can predict tumor response to immunotherapy (Ellegren, 2004; Baretti and Le, 2018; Steuer and Ramalingam, 2018). Initially, we examined the relationship between SF3B6 gene expression and eight well-known immune checkpoint molecules: CD274, CTLA4, HAVCR2, LAG3, PDCD1, PDCD1LG2, SIGLEC15, and TIGIT. Our findings indicated that SF3B6 was positively linked to the majority of immune checkpoint molecules in LIHC, LGG, KIRP, BLCA, PCPG, and KICH, whereas it exhibited negative associations with most immune checkpoint molecules in THYM, HNSC, ESCA, LUAD, LUSC, TGCT, and OV (Figure 6A).

Next, we assessed the association of SF3B6 gene expression with MSI, TMB, and the expression of six MMR-related genes (EPCAM, PMS2, MSH, MSH2, MLH1). SF3B6 gene expression was positively linked to most MMR-related genes across all tumor types, except in DLBC and UCS (Figure 6B). In 17 cancer types, including BLCA, BRCA, CESC, DLBC, ESCA, GBM, HNSC, KIRP, LGG, LIHC, LUAD, LUSC, OV, PRAD, READ, SKCM, STAD, SF3B6 gene expression demonstrated a positive relationship with MSI, while a negative relationship was detected in THYM (Figure 6C). Additionally, in nine cancer types, including ACC, BLCA, BRCA, LGG, LUAD, LUSC, PAAD, SKCM, and STAD, SF3B6 gene expression showed a positive relationship with TMB. Conversely, it demonstrated a negative relationship with TMB in KIRP, THCA, THYM, and UCEC (Figure 6D).

The immune phenotype scores, developed based on the expression of immune-related genes, including MHC molecules, immune regulators, effector cells, and suppressor cells, can predict patient responses to CTLA-4 and PD-1 blockade therapies (Charoentong et al., 2017). We assessed the association of SF3B6 gene expression with immune phenotype scores. Results indicated that in COAD, KIRC, LIHC, LUAD, STAD, THCA, and UCEC, patients with low SF3B6 expression were expected to achieve better immunotherapy outcomes when treated with CTLA-4 or PD-1 blockade (Figures 6E–K).

### 2.7 Functional enrichment analysis of SF3B6 in pan-cancer

**FIGURE 7:**
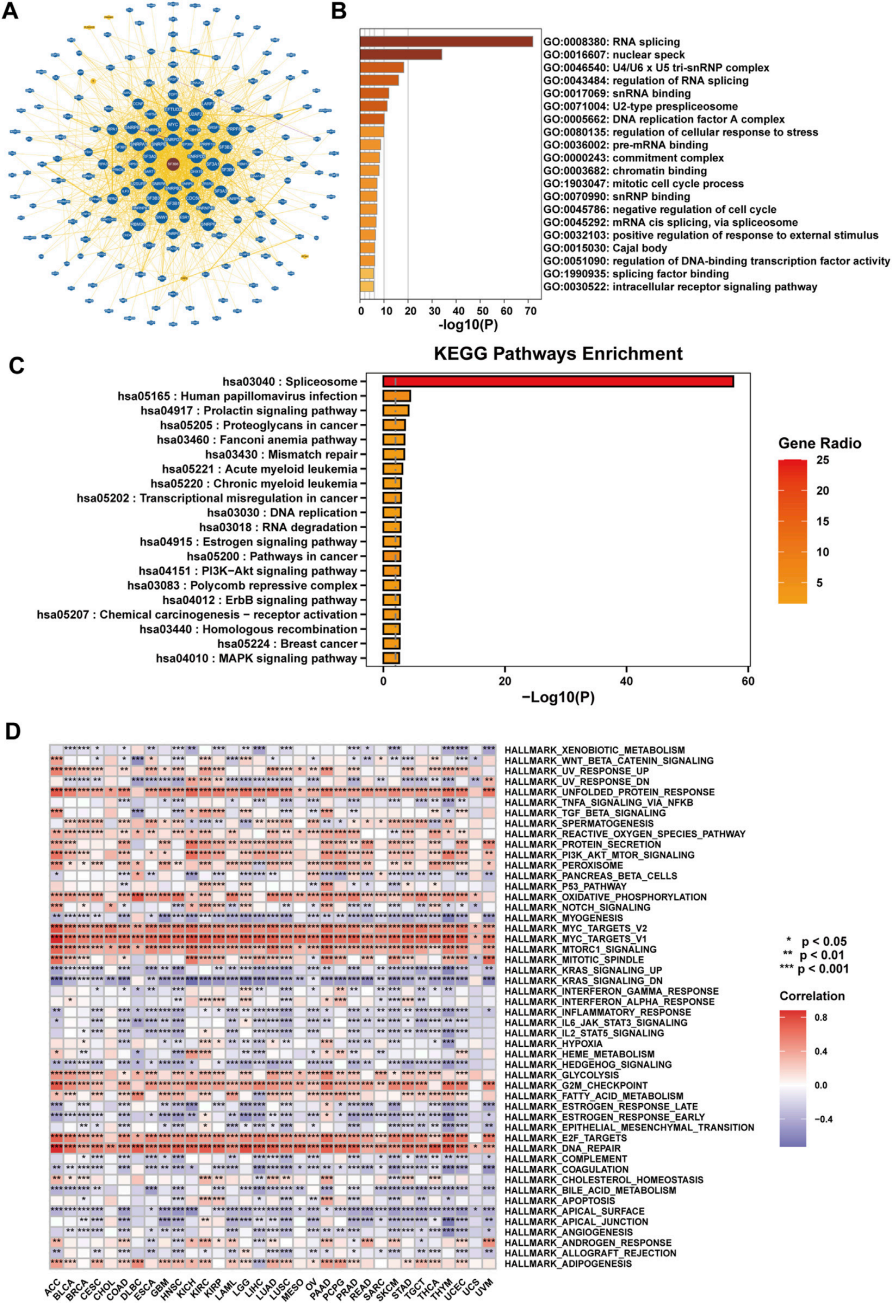
Functional enrichment analysis of SF3B6 in pan-cancer. **(A)** PPI network of SF3B6 and its interacting genes based on the BioGRID database. **(B)** GO function enrichment analysis of SF3B6-interacting proteins based on Metascape. **(C)** KEGG functional enrichment analysis based on SF3B6-interacting proteins by Metascape. **(D)** Heatmap showing the correlation between SF3B6 and HALLMARK pathway enrichment scores based on GSVA in pan-cancer.

To investigate the role of SF3B6 across pan-cancer, we initially retrieved a network interaction diagram of proteins and genes that interact with SF3B6 from the BioGRID database (Figure 7A). This network comprised 190 interacting proteins or genes and 283 interaction relationships. Using Metascape, we conducted GO and KEGG analyses on the acquired genes, revealing that the genes associated with SF3B6 were primarily enriched in pathways involved in RNA splicing, DNA replication, and the cell cycle (Figure 7B). The KEGG analysis demonstrated that the genes interacting with SF3B6 were enriched in pathways related to splicing, mismatch repair, DNA replication, RNA degradation, and several cancer-related pathways. Notable pathways included the prolactin signaling pathway, proteoglycans in cancer, Fanconi anemia pathway, PI3K−Akt signaling pathway, ErbB signaling pathway, and MAPK signaling pathway, among others (Figure 7C).

We then employed Gene Set Variation Analysis (GSVA) to examine the correlation of SF3B6 with HALLMARK pathways across different cancers. The analysis suggested that SF3B6 was significantly related to several immune response pathways, including the IL2-STAT5 signaling pathway, IL6-JAK-STAT3 signaling pathway, and inflammatory response pathways. Additionally, SF3B6 demonstrated associations with tumor metabolic pathways, such as the unfolded protein response, reactive oxygen species pathways, oxidative phosphorylation, mTORC1, glycolysis, fatty acid metabolism, and protein secretion. Furthermore, it was linked to classic tumor-related pathways, including KRAS, PI3K-AKT-mTOR, and MYC, as well as cell cycle and DNA repair pathways, such as the G2M checkpoint and E2F targets. SF3B6 also exhibited correlations with intercellular adhesion pathways, such as apical surface and tight junctions, in the majority of tumors (Figure 7D).

### 2.8 Analysis of SF3B6 differential expression in HCC, correlation with clinical pathological features, prognosis, and treatment response

**FIGURE 8:**
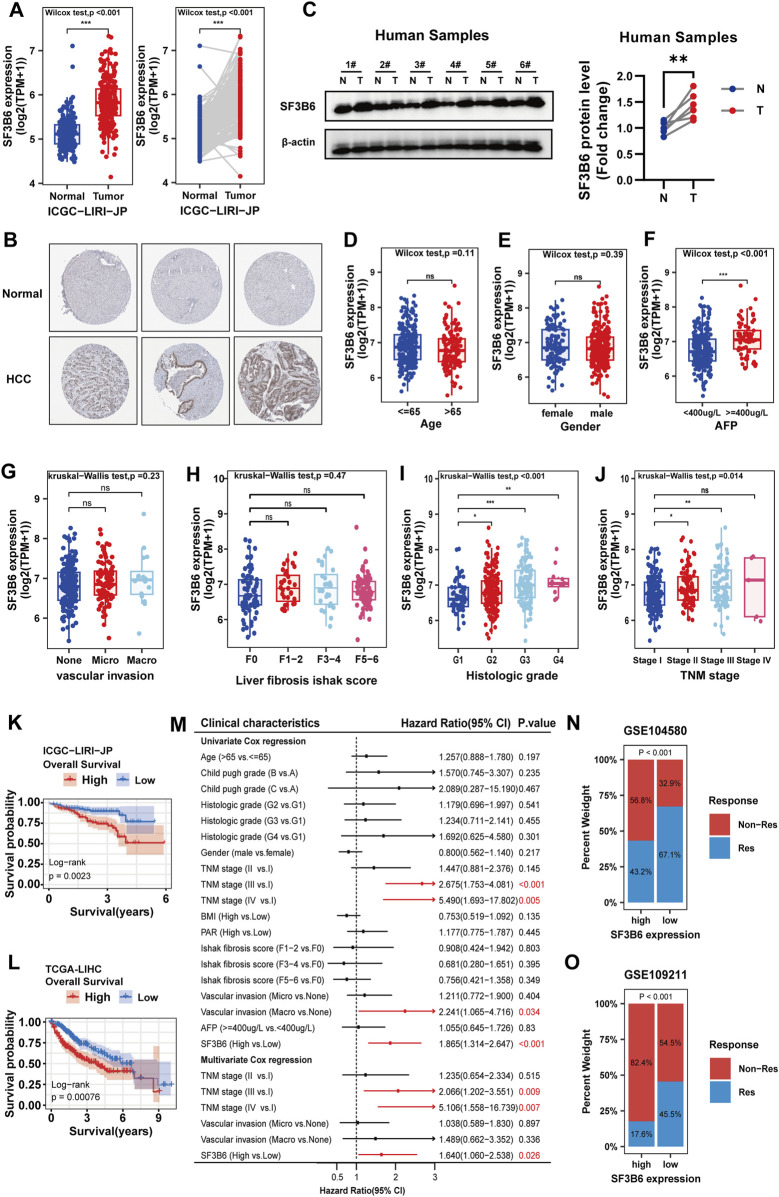
Differential expression of SF3B6, association between SF3B6 expression and clinicopathologic characteristics, and the prognostic predictive value of SF3B6 in HCC patients. **(A)** Unpaired and paired differential analysis of the mRNA expression of SF3B6 in tumor samples and adjacent normal samples based on the ICGC-LIRI-JP dataset.**(B)** Representative images of immunohistochemical staining for SF3B6 in normal liver and HCC tissues from the Human Protein Atlas database. **(C)** Protein expression levels of SF3B6 in six paired human HCC tumor specimens (T) and adjacent non-tumor tissues (N) were determined by Western blotting. Correlation analysis of mRNA expression of SF3B6 based on the TCGA-LIHC dataset with **(D)** age, **(E)** gender, **(F)** AFP, **(G)** vascular invasion, **(H)** liver fibrosis, **(I)** pathological grade and **(J)** TNM stage in HCC patients. Kaplan Meier curve of SF3B6 expression based on **(K)** ICGC-LIRI-JP dataset and **(L)** TCGA-LIHC for OS in HCC patients. **(M)** Forest plot showing the univariate and multivariate COX analyses of OS based on the TCGA-LIHC dataset in HCC patients. **(N)** Distribution of patients with different sorafenib therapy response statuses across high and low SF3B6 expression groups. **(O)** Distribution of patients with different TACE therapy response statuses across high and low SF3B6 expression groups. *p < 0.05, **p < 0.01, ***p < 0.001. ns not significant.

In addition to the TCGA-LIHC and CPTAC datasets, we further utilized the ICGC-LIRI-JP dataset as the validation cohort to investigate the differential expression of SF3B6 in patients with HCC. Consistently, both paired and unpaired differential expression analyses demonstrated significantly elevated levels of SF3B6 expression (Figure 8A). Moreover, immunohistochemical staining results from the Human Protein Atlas (HPA) database showed stronger SF3B6 staining intensity in HCC tissues than in normal liver tissues (Figure 8B). To validate the reliability of these findings, we performed Western blot analysis on six paired samples of HCC and adjacent normal tissues, which further confirmed the upregulation of SF3B6 expression in human HCC tumor tissues (Figure 8C).

Next, we explored the relationship between SF3B6 expression and clinical pathological features using TCGA-LIHC data. Results showed a positive correlation between elevated SF3B6 expression and alpha-fetoprotein (AFP) levels (p = 1.6e−05), TNM stage (p = 0.014), and histological grade (p = 2.2e−05), with no correlations found for age, gender, liver fibrosis score, or vascular invasion (Figures 8D–J). Kaplan-Meier survival analysis of the ICGC-LIRI-JP and TCGA-LIHC datasets indicated that higher SF3B6 expression was associated with shorter OS in HCC patients (Figures 8K,L). To control for confounding factors, we conducted univariate and multivariate Cox regression analyses, confirming that elevated SF3B6 expression is an independent risk factor for OS (Figure 8M). Additionally, we analyzed the GSE109221 cohort (sorafenib treatment) and the GSE104580 cohort (TACE treatment), revealing that patients with higher SF3B6 expression showed significantly greater resistance to both treatments (Figures 8N,O).

### 2.9 Functional enrichment analysis of SF3B6 in HCC

**FIGURE 9:**
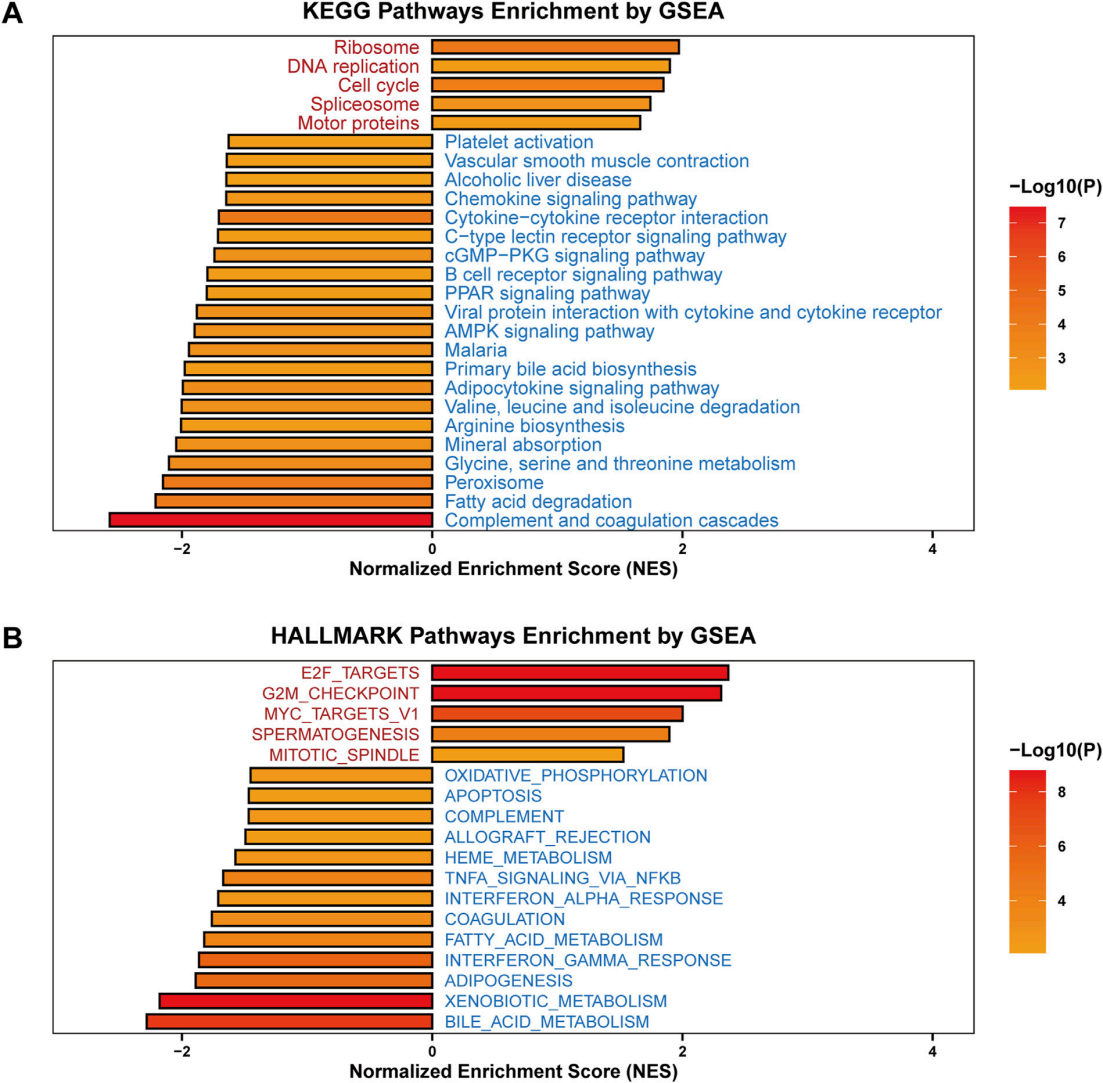
Functional enrichment analysis of SF3B6 in LIHC. **(A)** KEGG enrichment analysis on SF3B6 based on GSEA. **(B)** HALLMARK enrichment analysis on SF3B6 based on GSEA.

To examine the potential mechanisms underlying SF3B6 in HCC, we performed GSEA based on the TCGA-LIHC database, comparing patients with high versus low expression of SF3B6. The HALLMARK pathway enrichment analysis demonstrated a positive association between increased SF3B6 expression and pathways involving ribosomes, DNA replication, the cell cycle, spliceosomes, and motor proteins. Conversely, SF3B6 expression was significantly negatively correlated with various signaling pathways that influence tumor immunity, including platelet activation, vascular smooth muscle contraction, chemokine signaling, cytokine-cytokine receptor interaction, B cell receptor signaling, viral protein interactions with cytokines and their receptors, malaria, the coagulation complement cascade, and C-type lectin receptor signaling pathways. Additionally, SF3B6 expression exhibited negative correlations with tumor metabolism-related pathways, including alcoholic liver disease, the PPAR signaling pathway, the AMPK signaling pathway, primary bile acid synthesis, adipokine biosynthesis, valine/isoleucine/leucine degradation, arginine biosynthesis, mineral absorption, glycine/serine/threonine metabolism, peroxisome metabolism, cGMP-PKG signaling pathway, and fatty acid degradation (Figure 9A).

The KEGG pathway enrichment analysis results indicated significant positive relationships between high SF3B6 expression and pathways linked to cell proliferation, including E2F targets, the G2M checkpoint, spermatogenesis, MYC target V1, and mitosis/spindle pathways. By comparison, increased SF3B6 expression was significantly negatively related to apoptosis and tumor immunity-related pathways, such as complement activation, oxidative phosphorylation, allograft rejection, heme metabolism, NF-κB/TNF-α signaling, coagulation, interferon-gamma response, and interferon-alpha response. SF3B6 also negatively correlated with tumor metabolism-related pathways, including fatty acid synthesis, xenobiotic metabolism, bile acid metabolism, oxidative phosphorylation, and fatty acid metabolism. These findings corroborated the results from Gene Set Variation Analysis (GSVA) utilizing the TCGA-LIHC dataset (Figure 9B).

### 2.10 Construction and functional analysis of the protein-protein interaction (PPI) network of SF3B6-related survival alternative splicing events (SF3B6-SASEs)

**FIGURE 10:**
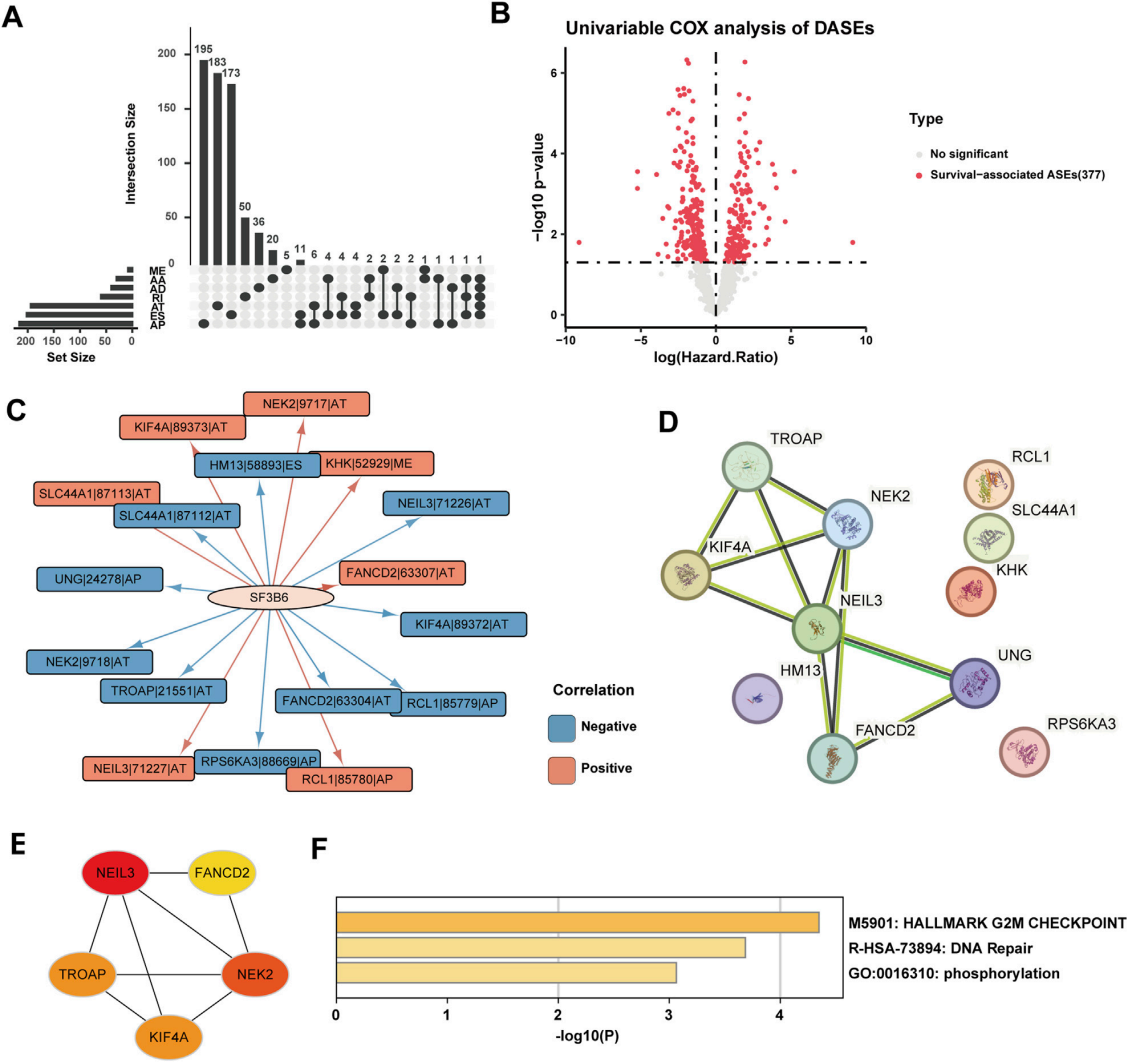
SF3B6-SASE parent-gene protein interaction network (PPI) construction and functional enrichment analysis. **(A)** The Upset plot showing the differential alternative splicing events (DASE) in the LIHC. **(B)** Volcano plot showing the result of the univariate COX analysis of DASE in LIHC. **(C)** The interaction network between SF3B6 and SF3B6-SASEs. **(D)** The PPI network of the SF3B6-SASE parental genes. **(E)** PPI network of core parental genes with the top 5 strongest interactions in SF3B6-SASEs. **(F)** Functional enrichment analysis of the SF3B6-SASE core parental genes based on Metascape.SF3B6-SASE, SF3B6-related survival-alternative splicing events. PPI, Protein-Protein Interaction.

**FIGURE 11:**
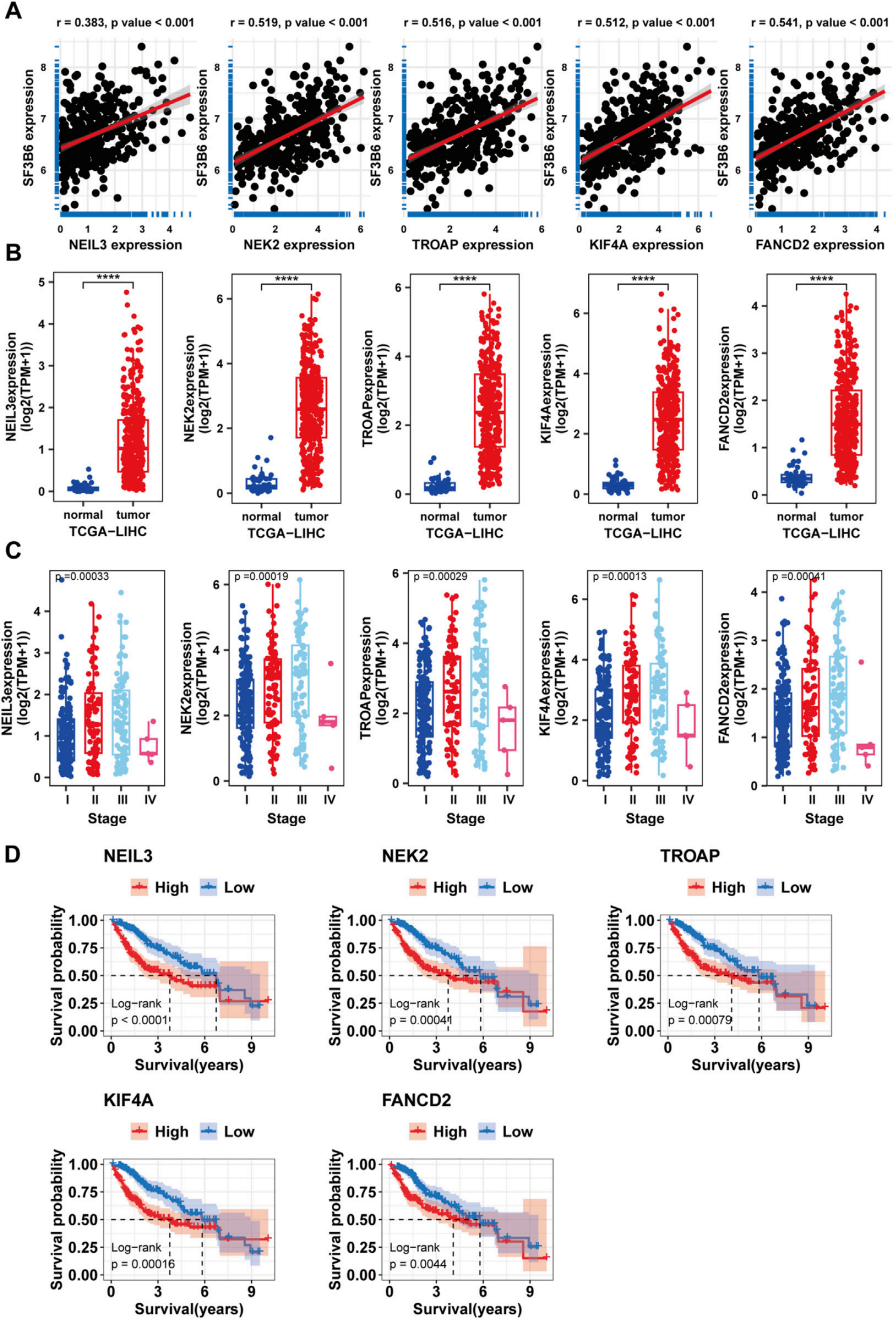
Differential expression analysis survival and clinical case characteristic analysis of hub genes. **(A)** Correlations between hub genes and SF3B6. **(B)** Differential expression analysis of hub genes between normal and tumor tissues in HCC. **(C)** Correlations between hub genes and clinical features. **(D)** Kaplan–Meier survival curves for patients with HCC malignancies stratified by hub genes expression levels.

Considering the importance of SF3B6 as a splicing factor in RNA splicing, we further identified key alternative splicing events associated with SF3B6. We obtained the splicing percentage in (PSI) values of ASEs for HCC from the TCGASplicingSeq database. The paired sample differential analysis results showed that, under the set threshold, we identified 1,177 differential alternative splicing events (DASEs) from 704 genes (Figure 10A; Supplementary Table S2). Based on the DASEs, we identified 379 SASEs from 241 genes using univariate Cox regression analysis (p < 0.05) (Figure 10B; Supplementary Table S3). To identify SF3B6-SASEs, we carried out a correlation analysis to evaluate the association between the PSI values of the SASEs and the gene expression levels of SF3B6. Ultimately, we identified 17 SF3B6-SASEs from 11 genes (Figure 10C; Supplementary Table S4). To investigate the potential biological functions of SF3B6-SASEs, we retrieved the PPI network of the parent genes from the STRING database (Figure 10D). We then calculated and visualized the five hub genes with the strongest interactions within the PPI network utilizing the MCC algorithm, including NEIL3, NEK2, KIF4A, TROAP, and FANCD2 (Figure 10E). Utilizing Metascape for functional enrichment analysis of these hub genes, we observed that they were primarily enriched in pathways associated with the G2M checkpoint, DNA repair, and phosphorylation (Figure 10F). Spearman correlation analysis suggested a markedly positive relationship of the SF3B6 expression with the five hub genes (Figure 11A).

Furthermore, we validated the correlation of the hub genes with the clinical characteristics and prognosis of HCC patients. The differential expression analysis suggested that the hub genes showed increased expression in HCC (Figure 11B), and their expression showed a positive relationship with histological staging (Figure 11C). Kaplan-Meier survival curve analysis suggested that elevated expression levels of the hub genes were associated with worse patient prognosis (Figure 11D).

### 2.11 Knockdown of SF3B6 suppressed proliferation, migration, and invasion of HCC cells

**FIGURE 12:**
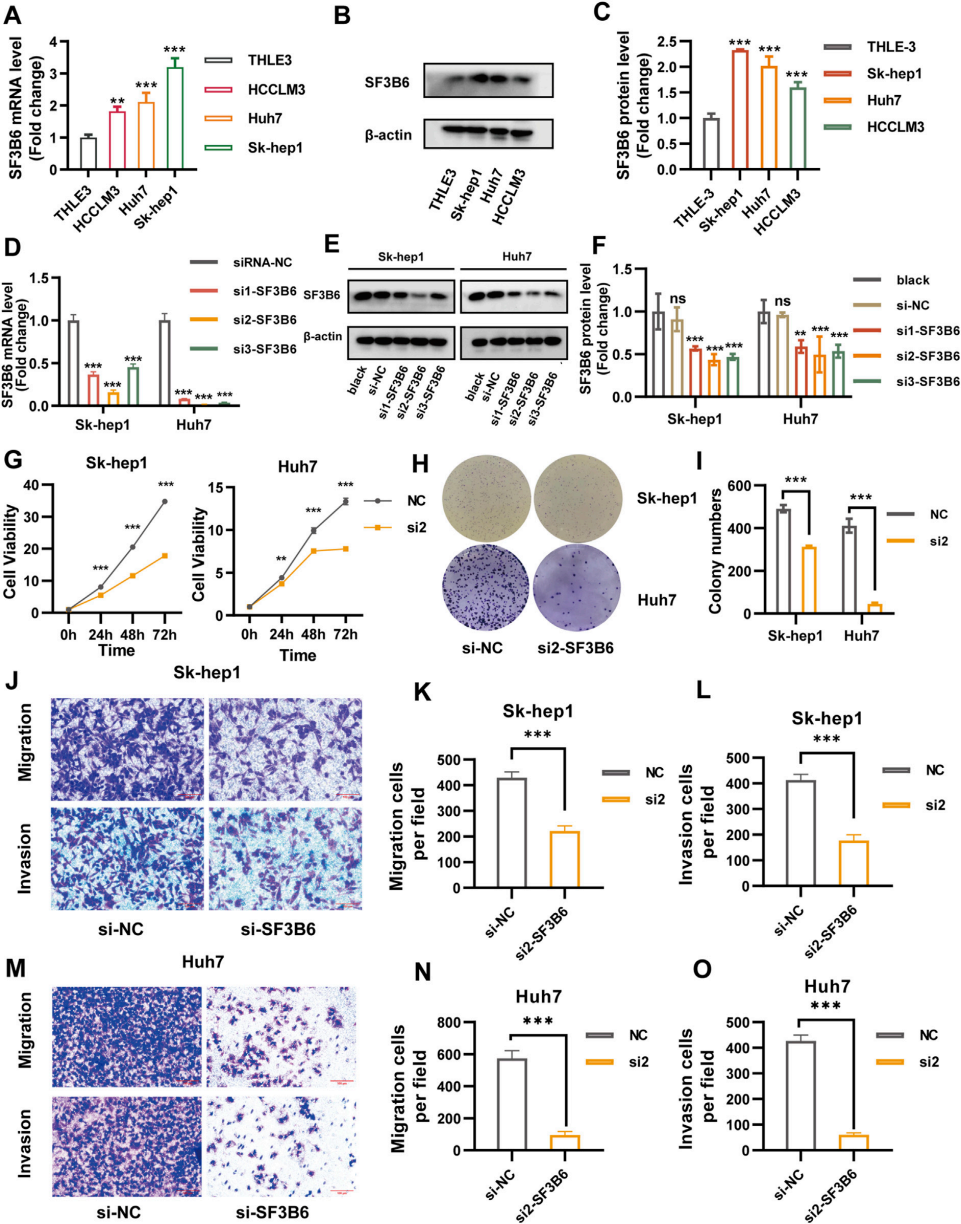
Knockdown of SF3B6 inhibited the proliferation, migration, and invasion ability of HCC cells. **(A)** The mRNA expression levels of THLE 3, Sk-hep1, Huh7, and HCCLM3 cells were determined by RT-qPCR. **(B, C)** Protein expression levels of THLE 3, Sk-hep1, Huh7, and HCCLM3 cells were determined by Western blotting. **(D)** RT-qPCR was used to detect the mRNA expression of SF3B6 in Sk-hep1 and Huh7 cells transfected with siRNA. **(E, F)** Western blotting was used to detect the protein expression of SF3B6 in Sk-hep1 and Huh7 cells after siRNA transfection. **(G)** CCK8 reagent was used to detect the proliferation of Sk-hep1 and Huh7 cells after knocking down SF3B6. **(H, I)** Colony formation assay detected the proliferation and colony formation of Sk-hep1 and Huh7 cells after knocking down SF3B6. **(J–L)** Transwell migration and invasion assay was used to detect the migration and invasion ability of Sk-hep1 cells after knocking down SF3B6. **(M–O)** Transwell migration and invasion assay was used to detect the migration and invasion ability of Huh7 cells after knocking down SF3B6. Data are shown as mean ± SD (*n* = 3 biologically independent samples per group). *p < 0.05; **p < 0.01; ***p < 0.001. ns not significant.

To investigate the expression of SF3B6 in liver cancer cell lines, we conducted RT-qPCR and Western blotting on a normal human liver epithelial cell line (THLE-3) and three liver cancer cell lines: Sk-hep1, Huh7, and HCCLM3. The RT-qPCR results displayed that the mRNA levels of SF3B6 were markedly elevated in the Sk-hep1, Huh7, and HCCLM3 liver cancer cell lines (Figure 12A). Similarly, the Western blotting results displayed that the protein expression levels of SF3B6 were elevated in these liver cancer cell lines (Figures 12B,C). Based on the higher expression of SF3B6 in SK-HEP1 and Huh7 cells, these two cell lines were selected for subsequent functional validation experiments.

To explore the impact of SF3B6 on HCC cell functions, we employed siRNAs to silence SF3B6 expression in Sk-hep1 and Huh7 cells. Both RT-qPCR and Western blotting demonstrated that siRNA2 exhibited the greatest knockdown efficiency. Thus, siRNA2 was chosen for further functional validation (Figures 12D–F). CCK-8 proliferation assays and colony formation assays demonstrated that silencing SF3B6 markedly reduced the proliferation capacity of HCC cells (Figures 12G–I). Furthermore, Transwell assays indicated that silencing SF3B6 markedly inhibited the migration and invasion abilities of HCC cells (Figures 12J–O).

### 2.12 Knockdown of SF3B6 increased apoptosis and caused S phase arrest in HCC cells

**FIGURE 13:**
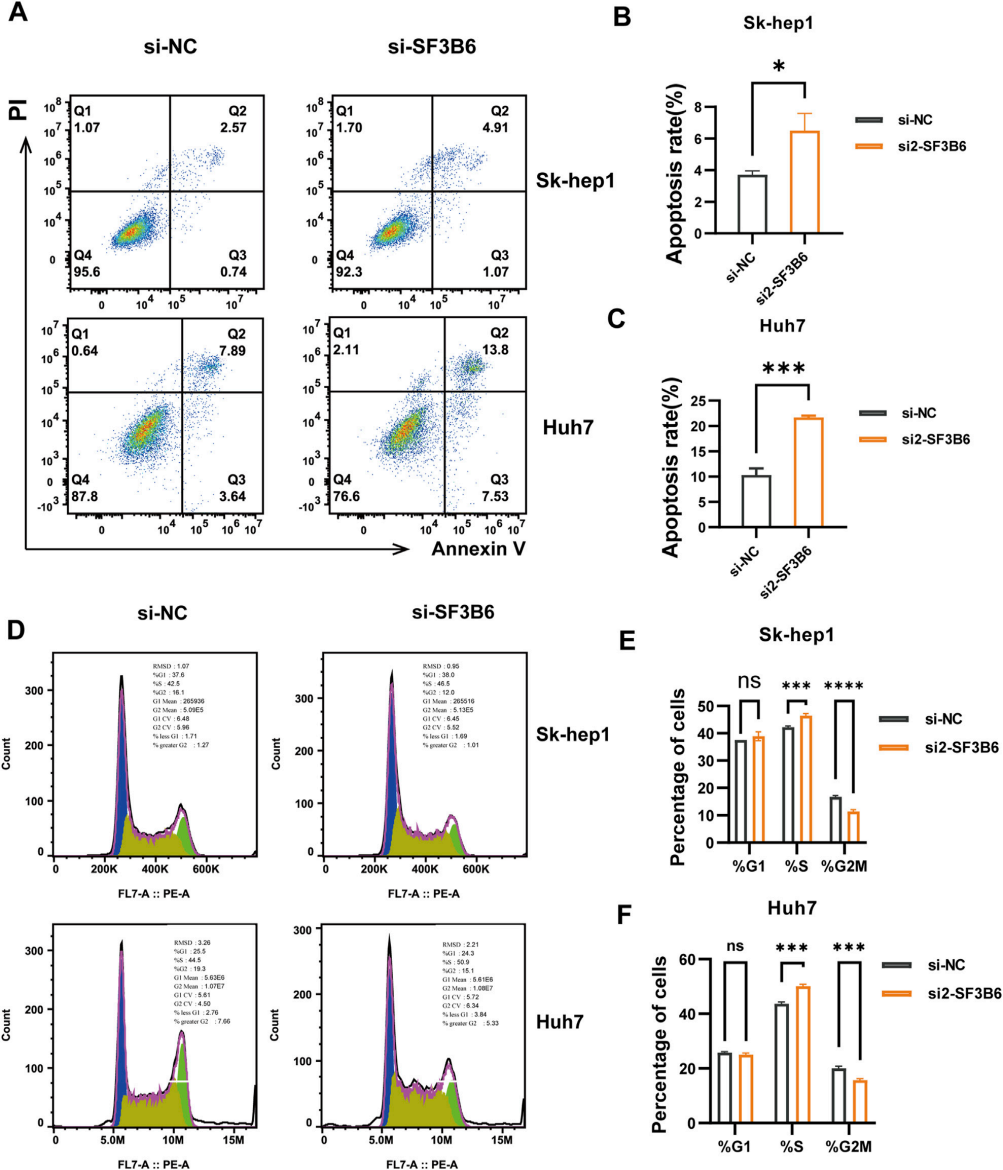
Knockdown of SF3B6 increased apoptosis and caused S phase arrest in HCC cells. **(A–C)** Flow cytometry detected the apoptosis rate of Sk-hep1 and Huh7 cells after knocking down SF3B6 **(D–F)** Flow cytometry was used to detect the cell cycle. Data are shown as mean ± SD (*n* = 3 biologically independent samples per group). *p < 0.05; **p < 0.01; ***p < 0.001. ns not significant.

Previous analyses of functional enrichment suggested a significant relationship between SF3B6 expression and both apoptosis and cell cycle processes. Therefore, we assessed the influence of SF3B6 on apoptosis and the cell cycle in HCC cell lines using flow cytometry. The results demonstrated that silencing SF3B6 markedly elevated the apoptosis rate in HCC cells (Figures 13A–C). Additionally, following the knockdown of SF3B6, there was an increase in the proportion of cells in the S phase, a reduction in the G2/M phase, and no significant change in the G1 phase. This result indicated that knocking down SF3B6 led to S phase arrest in HCC cells (Figures 13D–F).

## 3 Discussion

Dysregulation of alternative splicing is prevalent in multiple cancers and facilitates tumor development (Choi et al., 2023a; Choi et al., 2023b). RNA splicing factors and their splice variants can serve as biomarkers and therapeutic targets in cancer. During mRNA splicing, SF3B6, a crucial subunit of the SF3B complex, promotes the assembly, activation, and catalysis of the spliceosome by recognizing and binding to the branch point adenosine in pre-mRNA, thus facilitating stable interactions between U2 snRNA and the branch point sequence (Dybkov et al., 2006; Schellenberg et al., 2011; Tholen et al., 2022; Zhang et al., 2024). Previous studies of SF3B6 in tumors have been limited to PDAC, breast cancer, and NSCLC (Siebring-van Olst et al., 2017; Wu et al., 2019; Li J. et al., 2021; Peng et al., 2022). Furthermore, little is known about the role of SF3B6 in other cancer types. This research is the first to provide a comprehensive analysis of SF3B6 expression levels, clinical relevance, and potential roles across pan-cancer, further exploring its mechanisms in HCC through bioinformatics and *in vitro* experimental methods.

Our findings align with earlier research, demonstrating that SF3B6 was significantly elevated in the majority of tumor tissues, including those from breast cancer, pancreatic cancer, lung cancer, liver cancer, and so on. This upregulation could be regulated by copy number variation and DNA methylation. SF3B6 exhibited high diagnostic value in 16 types of tumors, including LIHC and LUAD, and so on (AUC >0.9). Prognostic analyses indicated that elevated SF3B6 expression served as a risk factor for OS, DFI, DSS, and PFI in patients with ACC, KIRP, LIHC, and LUAD. Functional enrichment analyses yielded consistent results across various cancers, indicating that SF3B6 expression was closely related to RNA splicing, DNA replication, cell cycle regulation, DNA damage repair, pathways associated with multiple cancer types, immune response pathways, tumor metabolic pathways, and classical tumor-related pathways (e.g., KRAS, PI3K/AKT/mTOR, MYC). These results strongly suggest that SF3B6 promotes tumorigenesis and progression, indicating its potential as a biomarker for guiding cancer diagnosis and prognosis.

The tumor microenvironment comprises various cells within the tumor (tumor cells, immune cells, stromal cells), vascular systems, extracellular matrices, and secreted factors, all of which are closely linked to immunotherapy. Immune infiltration analyses based on the XCELL and ESTIMATE algorithms suggested that SF3B6 showed a negative relationship with the levels of infiltration by immune and stromal cells across various cancer types. In most cancer types, SF3B6 displayed a positive relationship with CD4^+^ Th2 T cells and common lymphoid progenitors, while demonstrating a negative correlation with various other immune and stromal cells.CD4+ Th2 T cells are a major subclass of helper CD4^+^ T cells [32]. Cytokines produced by Th2 cells, including IL-10, IL-4, and TGF-β, can promote the dissemination and metastasis of cancer cells in various cancers. An imbalance between the Th1/Th2 cell ratio and their associated cytokines is linked to reduced PFI and OS in patients with breast cancer, melanoma, ovarian cancer, esophageal cancer, and colorectal cancer (Saravia et al., 2019). Our study indicates that SF3B6 may induce tumor immune suppression by promoting the differentiation of Th2 cells.

Considering the correlation between SF3B6 and the tumor microenvironment along with cell infiltration, we analyzed the predictive significance of SF3B6 for immunotherapy responses. Elevated expression of inhibitory immune checkpoints in the tumor microenvironment is linked to diminished T cell-mediated anti-tumor immune responses. Immune checkpoint inhibitors are monoclonal antibodies that selectively block these inhibitory immune checkpoints, promoting T cell-mediated anti-tumor immune responses (Wang et al., 2024). Our results demonstrated that SF3B6 expression was positively related to many immune checkpoint molecules in six types of cancer, including LIHC, while demonstrating a negative correlation with most immune checkpoint molecules in seven additional cancers, including LUAD and HNSC. Previous studies have shown that TMB, MSI, and MMR status have predictive value for tumor immunotherapy response (Ellegren, 2004; Baretti and Le, 2018; Steuer and Ramalingam, 2018). We demonstrated that SF3B6 was positively related to the majority of MMR-related genes across all tumor types except for DLBC and UCS. In specific cancer types, SF3B6 showed a significant association with MSI and TMB. Additionally, correlation analysis of immune phenotype scores indicated that in COAD, KIRC, LIHC, LUAD, STAD, THCA, and UCEC, tumor patients with low SF3B6 expression were anticipated to experience improved immunotherapy responses following treatment with CTLA-4 or PD-1 blockade (Van Allen et al., 2015; Hackl et al., 2016). This finding reveals that SF3B6 has the potential to function as a biomarker for predicting responses to immunotherapy in various cancers, including LIHC and LUAD.

We further explored the potential function of SF3B6 in HCC. Our results indicated that increased SF3B6 expression was positively associated with AFP levels, TNM staging, and histological grading in HCC. High expression of SF3B6 was associated with poor OS in HCC and was an independent risk factor for the disease. GSEA results suggested that SF3B6 was enriched in pathways associated with the cell cycle, DNA replication, apoptosis, tumor immunity, and tumor metabolism in HCC, indicating that SF3B6 may contribute to tumor cell proliferation, tumor microenvironment formation, and tumor metabolic reprogramming. Furthermore, in the GSE109211 and GSE104580 treatment cohorts for HCC, we observed that patients with high SF3B6 expression exhibited more pronounced resistance to sorafenib and TACE treatment. These findings indicate that SF3B6 is involved in the occurrence and progression of HCC, and it has the potential to serve as a prognostic biomarker and therapeutic target for this cancer.

Studies have shown that SF3B6 is vital for the regulation of alternative splicing, yet there is limited research on the target pre-mRNA of SF3B6. Therefore, we identified 17 SF3B6-related prognostic alternative splicing events from 11 parent genes, with the primary splice variant being alternative terminators. Subsequently, we employed the MCC algorithm to screen five hub genes with the strongest interactions: NEIL3, NEK2, KIF4A, TROAP, and FANCD2, all of which showed significant positive correlations with SF3B6 expression.NEIL3, a member of the DNA glycosylase family, plays a role in base damage repair and promotes hepatocellular carcinoma (HCC) advancement through mechanisms including telomere damage repair, enhanced phosphorylation of TWIST1, and activation of the PI3K/AKT/mTOR pathway (Chen et al., 2022). NEK2, a serine/threonine kinase, is essential in the cell cycle, and its dysregulation can lead to chromosomal instability, aneuploidy in cancer cells, activation of oncogenic signaling pathways, selective mRNA splicing, p53 loss, cilia disassembly, and tumor immune evasion (Xia et al., 2024). KIF4A, a kinesin family member, is essential for FOXM1-mediated proliferation of HCC cells, with its depletion linked to suppressed Akt kinase activity and induction of endogenous apoptotic signaling pathways (Hu G. et al., 2019).TROAP is critical for maintaining centriole integrity and spindle assembly during mitosis; its aberrant elevation enhances cyclin D1’s stability and nuclear localization through the DYRK1/Akt/GSK-3β signaling pathway, accelerating cell cycle progression and promoting malignant proliferation of HCC cells (Hu H. et al., 2019; Li L. et al., 2021). FANCD2, a key protein in the Fanconi anemia (FA) pathway, participates in DNA damage repair, cell cycle regulation, apoptosis, and chromatin remodeling, playing an important role in cancer via phosphorylation or ubiquitination (Brunner et al., 2023). In agreement with prior research, our study identified that these hub genes were enriched in the G2M checkpoint and DNA repair pathways, all being upregulated in HCC and strongly linked to worse prognosis and pathological staging in HCC patients. In conclusion, our findings demonstrate that SF3B6 may facilitate HCC development and progression by modulating the variable splicing of genes related to the G2M checkpoint and DNA repair pathways. Suppressing SF3B6 or targeting its associated splice variants with ASOs could represent a potential therapeutic approach for HCC.

Furthermore, we confirmed the high expression of SF3B6 in HCC tissues and cell lines. By establishing SF3B6 knockdown models and conducting cellular functional assays, we verified that SF3B6 enhanced the proliferation, migration, and invasion capabilities of HCC cells. Flow cytometric analysis also confirmed that SF3B6 inhibited apoptosis in HCC cells and modulated the transition from the S phase to the G2M phase in the cell cycle. These experimental findings validate the reliability of our bioinformatics analysis and lay the groundwork for future investigations of SF3B6 in HCC.

Despite these findings, several limitations and future directions warrant attention. First, pan-cancer analyses based on public databases such as TCGA and GTEx may be influenced by potential biases arising from sample heterogeneity, batch effects, and incomplete clinical annotations. Although experimental validation confirmed SF3B6 overexpression in HCC tissues and cell lines, the limited sample size may restrict the generalizability of diagnostic and prognostic models. Future studies should integrate multicenter clinical cohorts to systematically validate the correlation of SF3B6 expression patterns with clinical features, therapeutic responses and survival outcomes in HCC patients. Additionally, although *in vitro* experiments have confirmed the regulatory role of SF3B6 in the proliferation, migration, and apoptosis of HCC cells, the lack of animal models to validate its *in vivo* oncogenic function and its impact on the tumor microenvironment limits a comprehensive evaluation of its clinical translational potential in HCC. To address this issue, future research should employ conditional knockout or overexpression mouse models, syngeneic or xenograft models with genetically modified HCC cells, as well as patient-derived xenograft models. These models will facilitate the dynamic exploration of SF3B6’s regulatory networks in tumorigenesis, metastasis, and immune evasion, thus providing a more comprehensive theoretical foundation for its potential clinical applications. Furthermore, although bioinformatics analysis identified SF3B6-SASEs enriched in G2/M checkpoint and DNA repair pathways, the direct RNA targets of SF3B6 and its functional synergy with other SF3B complex subunits (e.g., SF3B1, PHF5A) remain to be elucidated. Advanced approaches such as CLIP-seq and RIP-seq are needed to map the RNA-binding sites of SF3B6. Additionally, the use of ASOs targeting key splice variants (e.g., NEK2, KIF4A) could provide insights into their downstream effects on cell cycle regulation and genomic stability. Moreover, proteomic and structural studies should explore the interaction interfaces between SF3B6 and other SF3B subunits, as well as their roles in spliceosome assembly. From a translational perspective, the development of SF3B6 inhibitors, along with their potential synergistic effects when combined with existing therapies (e.g., sorafenib, PD-1 inhibitors, chemotherapy drugs), could substantially accelerate the progress of personalized therapeutic strategies for HCC.

To summarize, this study represents the first comprehensive analysis of the correlation between SF3B6 expression and diagnosis, prognosis, immune infiltration, and immunotherapy response at the pan-cancer level. It explores the clinical significance and potential functions of SF3B6 and its related splice variants in HCC through bioinformatics and *in vitro* experimental approaches. This research lays a foundation and provides direction for future studies on the mechanisms and therapeutic implications of SF3B6 in cancer.

## 4 Materials and methods

### 4.1 Differential expression analysis of SF3B6 across pan-cancer

Gene expression data for SF3B6 was sourced from UCSC Xena (https://xenabrowser.net/datapages/) (Wang et al., 2016), normalized using the formula log2 (TPM+1), and combined with transcript expression data and clinical phenotype information. The R package “ggplot2” was utilized to examine and visualize the differences in SF3B6 expression between tumor and non-tumor samples. Tumor samples were obtained from the TCGA tumor dataset, while non-tumor samples were sourced from TCGA and GTEx. For the analysis of SF3B6 protein expression across different cancer types and their adjacent normal tissues, the “Proteomics” section of UALCAN (http://ualcan.path.uab.edu/) (Chandrashekar et al., 2022) was utilized, based on the CPTAC dataset (Uhlén et al., 2015).

### 4.2 Genetic variation analysis of SF3B6 in pan-cancer

The cBioPortal (http://www.cbioportal.org/) was employed to visualize the frequency of genetic alterations across various cancers sourced from TCGA (Cerami et al., 2012). Data on the CNV of the SF3B6 gene, along with gene expression and clinical features, were collected from UCSC Xena and integrated for analysis. Differences in SF3B6 gene CNV between tumor and non-tumor samples were evaluated and visualized using the R package “ggplot2.” Furthermore, correlations between SF3B6 gene CNV and expression levels were determined, with results illustrated using the “ggrepel” and “ggplot2” packages. Additionally, DNA methylation data for the SF3B6 gene, combined with its expression data from UCSC Xena, were analyzed to explore their relationship, employing “ggplot2” and “ggrepel” for visualization.

### 4.3 Diagnostic value analysis of SF3B6 in pan-cancer

Data on SF3B6 gene expression, obtained from UCSC Xena, were integrated with clinical phenotype information. The diagnostic potential of SF3B6 in differentiating tumor samples from non-tumor samples across various cancers was assessed using the ROC curve, computed with the “pROC” R package, and visualized using “ggplot2.” An AUC value approaching 1 indicates superior diagnostic accuracy. AUC values ranging from 0.5 to 0.7 suggest limited accuracy, those between 0.7 and 0.9 reflect good accuracy, while values from 0.9 to 1 signify high accuracy.

### 4.4 Prognostic value analysis of SF3B6 in pan-cancer

SF3B6 gene expression data obtained from UCSC Xena were merged with clinical information, focusing on samples that provided complete survival status and survival time data exceeding 30 days. Their relationships with OS, PFI, DFI, and DSS were examined through univariate Cox regression analysis, visualized using the “ezcox,” “survival,” “Survminer,” and “forestploter” R packages.

### 4.5 Immune infiltration analysis of SF3B6 in pan-cancer

Immune cell infiltration data obtained from TIMER2.0 (http://timer.cistrome.org/) (Li et al., 2017; Li et al., 2020), were combined with SF3B6 mRNA expression data from TCGA, focusing on data processed by the XCELL algorithm (Aran et al., 2017). The association of SF3B6 mRNA expression with various types of immune cell infiltration was calculated and visualized using the R package “ggplot2.” The ESTIMATE algorithm was employed to estimate the stromal score and immune score from the gene expression data. The sum of these two scores yields the ESTIMATE score, which is utilized to assess tumor purity. The correlation between SF3B6 mRNA expression levels and various ESTIMATE scores was examined and visualized with the R packages “ESTIMATE” and “ggplot2.”

### 4.6 Predictive value of SF3B6 for immunotherapy in pan-cancer

The TMB within tumor samples was calculated utilizing the “maftools” R package, and MSI scores were collected from prior studies. TMB, MSI scores, MMR-related genes, and immune checkpoint genes were integrated with SF3B6 gene expression data to assess their correlations, with results visualized using the “ggplot2” and “ggradar” R packages. The TCIA database (https://tcia.at/) provided comprehensive immunogenomic analysis results for 20 types of solid tumors from TCGA, including immunophenotype scores calculated from the expression of representative genes or gene sets related to MHC molecules, immune modulators, effector cells, and suppressor cells, which predict responses to anti-CTLA-4 and anti-PD-1 antibodies (Van Allen et al., 2015; Hackl et al., 2016; Charoentong et al., 2017). Immunophenotype scores for the 20 types of tumor samples were downloaded, and differences between groups with high and low levels of SF3B6 were calculated and visualized utilizing the “ggplot2” package in R.

### 4.7 Functional enrichment analysis of SF3B6 in pan-cancer

The BioGRID database was utilized to identify proteins or genes that interact with SF3B6. Metascape was employed to conduct Gene Ontology (GO) and KEGG enrichment analyses. The selection criteria included a minimum overlap of 3 and a minimum enrichment factor of 1.5, with a significance threshold set at P < 0.01. GSVA was performed and visualized using the R packages “GSEAbase,” “GSVA,” and “ggplot2.” This analysis yielded pathway-scoring results for each tumor sample, which were subsequently correlated with SF3B6 gene expression levels.

### 4.8 SF3B6 expression differential analysis in HCC

Gene expression profiles, along with phenotypic data were sourced from the ICGC-LIRI-JP dataset (https://dcc.icgc.org/), comprising 235 tumor samples and 202 corresponding normal samples. The gene expression profiles were standardized with the formula (TPM+1). The R package “ggplot2” was employed to analyze and visualize the SF3B6 gene expression levels. The HPA database (http://www.proteinatlas.org) was consulted to obtain information on SF3B6 immunohistochemistry staining, aiming to investigate the protein expression of SF3B6 in both HCC tissues and healthy control samples.

### 4.9 Correlation analysis of SF3B6 with clinical pathological features and treatment response in patients with HCC

The Kruskal-Wallis test or Wilcoxon test was employed to assess the relationship between SF3B6 and clinical pathological characteristics based on the TCGA-LIHC dataset. The study incorporated the GSE109221 cohort, which focused on sorafenib treatment for HCC, along with the GSE104580 cohort, which concentrated on TACE treatment for HCC. Differences in SF3B6 expression between the response and non-response groups were evaluated by employing the Chi-Square Test. The R package “ggplot2” was used for visualizations.

### 4.10 Functional enrichment analysis of SF3B6 in HCC

Using the TCGA-LIHC dataset, GSEA was executed employing the “clusterProfiler” package with the HALLMARK gene set (“h.all.v2023.2.Hs.symbols.gmt”) and the KEGG gene set (“c2.cp.kegg_legacy.v2023.2.Hs.symbols.gmt”) as reference datasets (Subramanian et al., 2005; Liberzon et al., 2015). A false discovery rate (FDR) of <0.25, an absolute normalized enrichment score (NES) > 1, and an adjusted P-value <0.01 were established as thresholds for statistical significance. The top 15 enriched terms were presented in a bar chart using the “ggplot2” package.

### 4.11 Screening of SF3B6-related survival alternative splicing events in HCC

The PSI values for splicing events of TCGA-LIHC were retrieved from the TCGASplicingSeq database (http://bioinformatics.mdanderson.org/TCGASpliceSeq). The PSI value is utilized to quantify each AS event, reflecting the ratio of normalized reads that include AS event transcript elements to total normalized reads, with a range from 0 to 1 (Ryan et al., 2016). To identify DASE in HCC, we employed the Wilcoxon paired sample non-parametric test to evaluate the Percent Spliced In PSI values in tumor samples compared to paired normal samples. DASEs were defined as samples where the change in the median PSI value between tumor samples and paired normal samples exceeded 0.1, along with a p-value of less than 0.05. To determine SASEs, we filtered DASEs utilizing univariate Cox regression analysis, defining DASEs with a P-value <0.05 as SASEs. We identified SF3B6-SASEs by conducting a Spearman correlation analysis to assess the association between SF3B6 gene expression and the PSI values of SASEs. SASEs with a p-value <0.05 and a correlation coefficient (R) > 0.4 were classified as SF3B6-SASEs.

### 4.12 Functional analysis of SF3B6-SASEs and construction of PPI network

To investigate the possible biological functions of SF3B6-SASEs, we used a minimum interaction score of >0.400 as the screening criterion to obtain the protein-protein interaction (PPI) data for the parent genes of SF3B6-SASEs from the STRING database. The top 5 hub genes within the PPI network were computed and visualized using the CytoHubba plugin in Cytoscape. Functional enrichment analysis for the hub genes was conducted utilizing Metascape.

### 4.13 Functional analysis of SF3B6-SASEs and construction of PPI network

To investigate the possible biological functions of SF3B6-SASEs, we used a minimum interaction score of > 0.400 as the screening criterion to obtain the protein-protein interaction (PPI) data for the parent genes of SF3B6-SASEs from the STRING database. The top 5 hub genes within the PPI network were computed and visualized using the CytoHubba plugin in Cytoscape. Functional enrichment analysis for the hub genes was conducted utilizing Metascape.

### 4.14 Cell culture and siRNA transfection

The normal human liver cell line THLE3 was acquired from ATCC (Manassas, VA, United States), while human liver cancer cell lines Sk-Hep1, Huh7, and HCCLM3 were sourced from the Institute of Biochemistry and Cell Biology (Shanghai, China). Cells were cultivated in DMEM containing 10% fetal bovine serum (FBS) incubated at 37°C with 5% CO2. siRNA transfection was conducted utilizing the Lipo2000 transfection reagent (Invitrogen, United States) following the manufacturer’s protocol. The sequences for the siRNA used in this study are detailed in Supplementary Table S5.

### 4.15 Patient samples collection

This study included 6 paired fresh-frozen tissue samples, consisting of hepatocellular carcinoma (HCC) and adjacent non-tumorous tissues, all obtained from patients who underwent hepatectomy at the Second Affiliated Hospital of Chongqing Medical University. The surgeries were performed between 10/2023 and 09/2024. Inclusion criteria: (1) Histologically confirmed HCC; (2) No preoperative chemotherapy or radiotherapy. Immediately after resection, both tumor and paired adjacent non-tumorous tissue samples were rapidly frozen in liquid nitrogen and stored at −80°C until protein extraction. The study protocol was approved by the Ethics Committee of the Second Affiliated Hospital of Chongqing Medical University (Approval No. 220/2022), and all participants provided written informed consent.

### 4.16 RNA extraction and real-time quantitative polymerase Chain PCR(RT-qPCR)

The RNA purification kit (ESScience, ES-RN001) was utilized to isolate total RNA from the cells in accordance with the manufacturer’s guidelines. The reverse transcription kit (Takara, RR047A) was utilized for reverse transcription. RT-qPCR was performed utilizing the qPCR kit (Accurate Biology, AG11701). The mRNA concentration for target genes was normalized utilizing the 2^−ΔΔCT^ method. The primer sequences employed in this experiment are provided in Supplementary Table S6.

### 4.17 Western blotting

To extract total protein from cells or tissues, a combination of RIPA lysis buffer and PMSF (Beyotime) was utilized. The extracted proteins were separated by 20% SDS-PAGE and then transferred to a PVDF membrane. The membrane was blocked by incubating it at room temperature for 2 h in 5% non-fat dry milk diluted in 1× TBST. Subsequently, the membrane was incubated overnight with primary antibodies diluted at a ratio of 1:2000 against SF3B6 (ProteinTech, 19677-1-AP) and beta-actin (ZENBIO, 380,624). After a 2-h incubation with the secondary antibody, the bands were exposed using ECL chemiluminescent substrate in a gel imaging system. Band intensity was analyzed utilizing ImageJ software.

### 4.18 CCK8 cell proliferation assay

After 36 h of siRNA transfection, 200 µL of cell suspension (totaling 4000 cells per well) was allocated to a 96-well plate. Cells were cultured overnight, after which a fresh CCK-8 working solution (100 µL DMEM high glucose medium +10 µL CCK-8) was poured into each well. After incubating at 37°C for 2.5 h, the absorbance at 450 nm was measured, representing the absorbance value at 0 h. This procedure was repeated every 24 h to record subsequent absorbance values.

### 4.19 Colony formation assay

After 36 h of siRNA transfection, 200 µL of cell suspension (totaling 4000 cells per well) was allocated to a 6-well plate. The cells were cultured at 37°C with 5% CO2 for 7–14 days, with daily observation and medium changes as necessary. When most individual cell colonies contained 50 cells or more, the culture was terminated. Cells were then fixed in 4% paraformaldehyde for 30 min, followed by staining with 1% crystal violet for an additional 30 min. Photographs of the cell colonies were taken and counted.

### 4.20 Transwell assay

Matrigel was purchased from Corning, and 8 μm 24-well Transwell chambers were obtained from BD Biosciences. Transwell chambers without Matrigel coating were employed to assess the cells’ migratory capacity, while Matrigel-coated Transwell chambers were used to evaluate cell invasion by mimicking the extracellular matrix (ECM) barrier. Medium supplemented with 20% FBS was used as the chemoattractant in the lower chamber. For the migration assay, cells were transfected with siRNA for 36 h, followed by a 12-h starvation in DMEM basal medium. The cells were then harvested and resuspended in serum-free DMEM at a density of 3 × 10^5 cells/mL. Next, 200 µL of the cell suspension (containing 6 × 10^4 Huh7 or Sk-hep1 cells) was added to the upper chamber, while the lower chamber was filled with DMEM supplemented with 20% FBS. After 48 h of incubation at 37°C, cells were fixed with 4% paraformaldehyde for 20 min, stained with 1% crystal violet for 20 min, and imaged under a light microscope. Cell counts were determined from five randomly selected fields. For the invasion assay, Matrigel was diluted 1:8 in DMEM and applied to the upper chamber. After solidification at 37°C for 3 h, excess Matrigel was removed, and the membrane was rehydrated with pre-warmed serum-free medium. The subsequent steps, including cell seeding, fixation, staining, and quantification, were performed as in the migration assay. Both siRNA-negative control (siRNA-NC) and siRNA-SF3B6 groups were tested in triplicate to ensure statistical reliability. Data are expressed as mean ± SD (n = 3 biologically independent samples per group), and statistical significance was determined using Student’s t-test.

### 4.21 Cell apoptosis detection

The cell apoptosis rate was measured using an apoptosis detection kit (Elabscience, E-CK-A320). Briefly, after 36 h of siRNA transfection, cells were digested with 0.25% trypsin without EDTA. Following the manufacturer’s guidelines, Annexin V-FITC and propidium iodide (PI) staining reagents were added to each tube containing 5 × 10^5 cells. The mixture was incubated for 20 min in the dark. Subsequently, the apoptosis rate was assessed using a flow cytometer within 1 hour.

### 4.22 Cell cycle detection

After 48 h of siRNA transfection, a cell cycle detection kit (Elabscience, E-CK-A351) was utilized to determine the distribution of cells across different phases following the manufacturer’s guidelines. The cell cycle distribution was assessed with a flow cytometer within 1 hour.

### 4.23 Statistical analysis

For celluar experiments, all quantitative measurements were obtained from a minimum of three independent biological replicates. Experimental results are presented as mean ± standard deviation (SD). R version 4.3.1 and GraphPad Prism 9.0 software were used for statistical analyses and plotting. Student’s t-test or ANOVA was employed for normally distributed continuous variables, while Wilcoxon or Kruskal-Wallis tests were applied for non-normally distributed variables. Spearman correlation analysis was conducted to determine the relationship between the two groups. The Kaplan-Meier curve was utilized to assess survival differences between high and low expression groups, with log-rank tests applied to evaluate statistical significance. To identify prognostic factors, univariate and multivariate Cox regression analyses were performed using a significance threshold of P < 0.05.

## Data Availability

The original contributions presented in the study are included in the article/Supplementary Material, further inquiries can be directed to the corresponding author.
