# Supplementary material for: Bioinformatics analysis across pan-cancer and experimental validation in hepatocellular carcinoma revealed the oncogenic role of SF3B6
Source: Front Pharmacol. 2025 Apr 28;16:1516534. doi: 10.3389/fphar.2025.1516534 (PMC12067594; doi:10.3389/fphar.2025.1516534)
Supplement: Supplementary file 1 [file Table1.docx]

Supplementary Table 1. Abbreviations of 33 types of cancer in TCGA database.

| **Cancer full name** | **Abbreviation** |
| --- | --- |
| Adrenocortical carcinoma | ACC |
| Bladder urothelial carcinoma | BLCA |
| Breast invasive carcinoma | BRCA |
| Cervical squamous cell carcinoma and endocervical adenocarcinoma | CESC |
| Cholangiocarcinoma | CHOL |
| Colon adenocarcinoma | COAD |
| Lymphoid neoplasm diffuse large B-cell lymphoma | DLBC |
| Esophageal carcinoma | ESCA |
| Glioblastoma multiforme | GBM |
| Head and Neck squamous cell carcinoma | HNSC |
| Kidney chromophobe | KICH |
| Kidney renal clear cell carcinoma | KIRC |
| Kidney renal papillary cell carcinoma | KIRP |
| Acute myeloid leukemia | LAML |
| Brain lower grade glioma | LGG |
| Liver hepatocellular carcinoma | LIHC |
| Lung adenocarcinoma | LUAD |
| Lung squamous cell carcinoma | LUSC |
| Mesothelioma | MESO |
| Ovarian serous cystadenocarcinoma | OV |
| Pancreatic adenocarcinoma | PAAD |
| Pheochromocytoma and paraganglioma | PCPG |
| Prostate adenocarcinoma | PRAD |
| Rectum adenocarcinoma | READ |
| Sarcoma | SARC |
| Skin cutaneous melanoma | SKCM |
| Stomach adenocarcinoma | STAD |
| Testicular germ cell tumors | TGCT |
| Thyroid carcinoma | THCA |
| Thymoma | THYM |
| Uterine corpus endometrial carcinoma | UCEC |
| Uterine carcinosarcoma | UCS |
| Uveal melanoma | UVM |

Supplementary Table 2. Summary of 1177 differential alternative splicing events (DASEs) in TCGA-LIHC dataset.

| **ASE** | **normalMedian** | **tumorMedian** | **Pvalue** | **Δmedian** |
| --- | --- | --- | --- | --- |
| DVL1\|141\|AD | 0.373 | 0.480 | 0.002 | 0.107 |
| CCNL2\|160\|ES | 0.448 | 0.631 | 0.020 | 0.182 |
| SLC35E2\|222\|AT | 0.846 | 0.723 | 0.001 | -0.124 |
| SLC35E2\|223\|AT | 0.154 | 0.277 | 0.001 | 0.124 |
| MORN1\|253\|AT | 0.873 | 0.726 | 0.000 | -0.147 |
| ACOT7\|388\|AP | 0.574 | 0.188 | 0.000 | -0.385 |
| ACOT7\|389\|AP | 0.344 | 0.603 | 0.002 | 0.259 |
| HES2\|400\|AT | 0.307 | 0.412 | 0.000 | 0.105 |
| HES2\|401\|AT | 0.693 | 0.588 | 0.000 | -0.105 |
| TMEM201\|564\|AT | 0.454 | 0.344 | 0.000 | -0.110 |
| TMEM201\|565\|AT | 0.546 | 0.656 | 0.000 | 0.110 |
| CLSTN1\|575\|ES | 0.600 | 0.382 | 0.008 | -0.218 |
| CASZ1\|621\|AT | 0.551 | 0.676 | 0.000 | 0.125 |
| CASZ1\|622\|AT | 0.449 | 0.324 | 0.000 | -0.125 |
| CLCN6\|686\|AT | 0.574 | 0.682 | 0.000 | 0.108 |
| NBPF1\|831\|AP | 0.411 | 0.308 | 0.001 | -0.103 |
| PADI4\|851\|AT | 0.460 | 0.142 | 0.000 | -0.318 |
| PADI4\|852\|AT | 0.540 | 0.869 | 0.000 | 0.329 |
| EPHB2\|1026\|RI | 0.813 | 0.601 | 0.001 | -0.212 |
| MAN1C1\|1192\|AP | 0.406 | 0.599 | 0.000 | 0.193 |
| MAN1C1\|1193\|AP | 0.594 | 0.401 | 0.000 | -0.193 |
| MTFR1L\|1210\|AA | 0.359 | 0.235 | 0.000 | -0.124 |
| MTFR1L\|1211\|ME | 0.810 | 0.673 | 0.001 | -0.137 |
| PIGV\|1299\|AP | 0.258 | 0.389 | 0.003 | 0.131 |
| PIGV\|1300\|AP | 0.728 | 0.606 | 0.001 | -0.122 |
| CSMD2\|1687\|AT | 0.974 | 0.713 | 0.000 | -0.261 |
| CSMD2\|1689\|AT | 0.019 | 0.244 | 0.000 | 0.225 |
| CLSPN\|1730\|AT | 0.000 | 0.321 | 0.000 | 0.321 |
| CLSPN\|1731\|AT | 1.000 | 0.679 | 0.000 | -0.321 |
| MACF1\|1881\|ES | 0.669 | 0.546 | 0.000 | -0.123 |
| PPCS\|2075\|AD | 0.572 | 0.731 | 0.000 | 0.159 |
| EBNA1BP2\|2145\|AP | 0.663 | 0.856 | 0.007 | 0.193 |
| EBNA1BP2\|2146\|AP | 0.337 | 0.144 | 0.007 | -0.193 |
| DMAP1\|2545\|RI | 0.601 | 0.710 | 0.003 | 0.109 |
| NASP\|2706\|ES | 0.230 | 0.401 | 0.000 | 0.171 |
| CYP4A11\|2857\|ES | 0.845 | 0.723 | 0.000 | -0.122 |
| TTC39A\|2950\|AT | 0.207 | 0.073 | 0.001 | -0.134 |
| TTC39A\|2951\|AT | 0.790 | 0.915 | 0.002 | 0.125 |
| ECHDC2\|3024\|ES | 0.905 | 0.791 | 0.000 | -0.114 |
| SCP2\|3045\|ES | 0.038 | 0.176 | 0.000 | 0.138 |
| YIPF1\|3079\|ES | 0.746 | 0.601 | 0.000 | -0.145 |
| TMEM59\|3108\|AP | 0.497 | 0.126 | 0.000 | -0.372 |
| TMEM59\|3111\|AP | 0.492 | 0.846 | 0.000 | 0.353 |
| C8B\|3190\|ES | 0.200 | 0.100 | 0.000 | -0.101 |
| FGGY\|3207\|AP | 0.767 | 1.000 | 0.000 | 0.233 |
| FGGY\|3208\|AP | 0.233 | 0.000 | 0.000 | -0.233 |
| LEPR\|3295\|AT | 0.494 | 0.357 | 0.000 | -0.137 |
| LEPR\|3297\|AT | 0.482 | 0.623 | 0.000 | 0.141 |
| PTGER3\|3416\|AT | 0.642 | 0.494 | 0.043 | -0.148 |
| PRKACB\|3584\|AP | 0.650 | 0.772 | 0.002 | 0.122 |
| PRKACB\|3585\|AP | 0.344 | 0.162 | 0.000 | -0.183 |
| GBP2\|3712\|AP | 0.824 | 0.511 | 0.000 | -0.313 |
| GBP2\|3713\|AP | 0.176 | 0.489 | 0.000 | 0.313 |
| TGFBR3\|3739\|AP | 0.440 | 0.304 | 0.035 | -0.136 |
| TGFBR3\|3746\|ES | 0.697 | 0.816 | 0.016 | 0.119 |
| BCAR3\|3793\|AP | 0.399 | 0.256 | 0.008 | -0.143 |
| BCAR3\|3795\|AP | 0.506 | 0.631 | 0.014 | 0.125 |
| GSTM2\|4057\|AT | 0.055 | 0.163 | 0.000 | 0.107 |
| GSTM2\|4061\|AT | 0.937 | 0.826 | 0.000 | -0.111 |
| KCNC4\|4098\|AA | 0.692 | 0.803 | 0.016 | 0.111 |
| LAMTOR5\|4114\|RI | 0.768 | 0.637 | 0.014 | -0.130 |
| ADORA3\|4174\|AT | 0.238 | 0.620 | 0.000 | 0.383 |
| ADORA3\|4175\|AT | 0.762 | 0.380 | 0.000 | -0.383 |
| GDAP2\|4377\|AT | 0.719 | 0.605 | 0.000 | -0.115 |
| GDAP2\|4378\|AT | 0.281 | 0.395 | 0.000 | 0.115 |
| PDE4DIP\|4407\|AP | 0.857 | 0.733 | 0.000 | -0.124 |
| PDE4DIP\|4410\|AT | 0.192 | 0.085 | 0.000 | -0.106 |
| RNF115\|7297\|ES | 0.360 | 0.122 | 0.000 | -0.237 |
| FMO5\|7372\|ES | 0.196 | 0.075 | 0.000 | -0.122 |
| FMO5\|7373\|RI | 0.571 | 0.725 | 0.003 | 0.154 |
| SEMA6C\|7562\|AD | 0.667 | 0.462 | 0.002 | -0.205 |
| SCNM1\|7563\|AP | 0.130 | 0.029 | 0.000 | -0.101 |
| SCNM1\|7564\|AP | 0.870 | 0.971 | 0.000 | 0.101 |
| UBAP2L\|7813\|AT | 0.442 | 0.575 | 0.000 | 0.133 |
| UBAP2L\|7814\|AT | 0.476 | 0.368 | 0.000 | -0.109 |
| ZBTB7B\|7878\|ES | 0.450 | 0.220 | 0.000 | -0.229 |
| ADAM15\|7903\|ES | 0.345 | 0.172 | 0.000 | -0.173 |
| ADAM15\|7904\|ES | 0.318 | 0.198 | 0.002 | -0.120 |
| ADAM15\|7911\|ES | 0.792 | 1.000 | 0.010 | 0.208 |
| ADAM15\|7919\|ES | 0.647 | 0.753 | 0.031 | 0.106 |
| SLC50A1\|7940\|AP | 0.195 | 0.085 | 0.000 | -0.110 |
| SLC50A1\|7941\|AP | 0.805 | 0.915 | 0.000 | 0.110 |
| LMNA\|8178\|AT | 0.436 | 0.325 | 0.000 | -0.111 |
| LMNA\|8179\|AT | 0.564 | 0.675 | 0.000 | 0.111 |
| PYHIN1\|8389\|AT | 0.708 | 0.808 | 0.031 | 0.100 |
| PYHIN1\|8390\|AT | 0.292 | 0.192 | 0.031 | -0.100 |
| APOA2\|8613\|ES | 0.508 | 0.405 | 0.002 | -0.104 |
| APOA2\|8614\|ES | 0.735 | 0.628 | 0.000 | -0.107 |
| NR1I3\|8622\|AT | 0.925 | 0.742 | 0.000 | -0.183 |
| NR1I3\|8624\|AT | 0.075 | 0.258 | 0.000 | 0.183 |
| NR1I3\|8626\|RI | 0.122 | 0.316 | 0.000 | 0.194 |
| NR1I3\|8628\|RI | 0.148 | 0.406 | 0.000 | 0.259 |
| NR1I3\|8629\|RI | 0.063 | 0.198 | 0.000 | 0.134 |
| NR1I3\|8631\|RI | 0.100 | 0.469 | 0.000 | 0.369 |
| NR1I3\|8632\|AA | 0.251 | 0.406 | 0.000 | 0.155 |
| NR1I3\|8633\|AA | 0.620 | 0.786 | 0.000 | 0.166 |
| NR1I3\|8634\|AD | 0.450 | 0.641 | 0.000 | 0.191 |
| NR1I3\|8635\|ES | 0.740 | 0.582 | 0.000 | -0.157 |
| NR1I3\|8637\|RI | 0.033 | 0.208 | 0.000 | 0.175 |
| NR1I3\|8639\|AA | 0.371 | 0.525 | 0.001 | 0.154 |
| NR1I3\|8641\|ES | 0.497 | 0.317 | 0.000 | -0.179 |
| NR1I3\|8642\|ES | 0.832 | 0.699 | 0.000 | -0.133 |
| NR1I3\|8644\|AD | 0.268 | 0.429 | 0.000 | 0.161 |
| NR1I3\|8645\|ES | 0.561 | 0.394 | 0.000 | -0.168 |
| NR1I3\|8647\|RI | 0.236 | 0.544 | 0.000 | 0.308 |
| UAP1\|8751\|ES | 0.617 | 0.461 | 0.000 | -0.157 |
| DCAF6\|8887\|ES | 0.661 | 0.561 | 0.000 | -0.100 |
| FMO3\|8976\|ES | 0.785 | 0.905 | 0.000 | 0.121 |
| GLUL\|9148\|AD | 0.328 | 0.437 | 0.039 | 0.109 |
| LAMC2\|9169\|AT | 0.168 | 0.408 | 0.000 | 0.240 |
| LAMC2\|9170\|AT | 0.832 | 0.592 | 0.000 | -0.240 |
| COLGALT2\|9202\|AT | 0.384 | 0.170 | 0.013 | -0.213 |
| COLGALT2\|9203\|AT | 0.616 | 0.797 | 0.015 | 0.180 |
| ASPM\|9286\|ES | 1.000 | 0.889 | 0.017 | -0.111 |
| NR5A2\|9331\|ES | 0.486 | 0.608 | 0.003 | 0.122 |
| KDM5B\|9420\|ES | 0.725 | 0.623 | 0.027 | -0.102 |
| ETNK2\|9466\|AP | 0.459 | 0.158 | 0.000 | -0.301 |
| ETNK2\|9467\|AP | 0.537 | 0.842 | 0.000 | 0.305 |
| NFASC\|9492\|AT | 0.793 | 0.654 | 0.000 | -0.139 |
| NFASC\|9493\|AT | 0.207 | 0.346 | 0.000 | 0.139 |
| FAM72A\|9575\|AP | 0.820 | 0.422 | 0.000 | -0.398 |
| FAM72A\|9576\|AP | 0.180 | 0.578 | 0.000 | 0.398 |
| FAM72A\|9577\|AT | 0.683 | 0.275 | 0.000 | -0.408 |
| FAM72A\|9578\|AT | 0.283 | 0.707 | 0.000 | 0.425 |
| PFKFB2\|9616\|AT | 0.571 | 0.415 | 0.000 | -0.156 |
| PFKFB2\|9617\|AT | 0.429 | 0.585 | 0.000 | 0.156 |
| NEK2\|9717\|AT | 0.355 | 0.813 | 0.000 | 0.458 |
| NEK2\|9718\|AT | 0.645 | 0.187 | 0.000 | -0.458 |
| ANGEL2\|9775\|AP | 0.253 | 0.364 | 0.002 | 0.111 |
| ANGEL2\|9776\|AP | 0.747 | 0.636 | 0.002 | -0.111 |
| SRP9\|9994\|ME | 0.853 | 0.970 | 0.000 | 0.116 |
| EPHX1\|9997\|AP | 0.306 | 0.570 | 0.000 | 0.264 |
| EPHX1\|9998\|AP | 0.694 | 0.430 | 0.000 | -0.264 |
| SNAP47\|10055\|AP | 0.411 | 0.300 | 0.004 | -0.111 |
| SNAP47\|10056\|AP | 0.590 | 0.700 | 0.004 | 0.111 |
| MRPL55\|10116\|ES | 0.449 | 0.313 | 0.000 | -0.136 |
| MRPL55\|10120\|ES | 0.291 | 0.184 | 0.033 | -0.107 |
| MRPL55\|10127\|ES | 0.763 | 0.639 | 0.000 | -0.124 |
| MRPL55\|10146\|ES | 0.651 | 0.527 | 0.005 | -0.124 |
| COA6\|10335\|AP | 0.746 | 0.846 | 0.000 | 0.101 |
| COA6\|10336\|AP | 0.254 | 0.154 | 0.000 | -0.101 |
| EFCAB2\|10479\|AT | 0.402 | 0.581 | 0.000 | 0.179 |
| EFCAB2\|10480\|AT | 0.598 | 0.419 | 0.000 | -0.179 |
| ZNF124\|10514\|AT | 0.491 | 0.368 | 0.000 | -0.123 |
| PRTFDC1\|11016\|AT | 0.257 | 0.429 | 0.000 | 0.172 |
| PRTFDC1\|11017\|AT | 0.743 | 0.571 | 0.000 | -0.172 |
| PARD3\|11211\|ES | 0.859 | 0.964 | 0.000 | 0.106 |
| PARD3\|11216\|ES | 0.715 | 0.564 | 0.000 | -0.151 |
| CREM\|11229\|AP | 0.172 | 0.365 | 0.000 | 0.193 |
| CREM\|11231\|AP | 0.545 | 0.248 | 0.000 | -0.297 |
| RTKN2\|11869\|AT | 0.308 | 0.416 | 0.004 | 0.108 |
| RTKN2\|11870\|AT | 0.000 | 0.154 | 0.000 | 0.154 |
| RTKN2\|11871\|AT | 0.651 | 0.393 | 0.000 | -0.258 |
| SLC25A16\|11952\|ES | 0.258 | 0.572 | 0.000 | 0.315 |
| AIFM2\|12029\|AT | 0.278 | 0.046 | 0.000 | -0.232 |
| AIFM2\|12030\|AT | 0.722 | 0.954 | 0.000 | 0.232 |
| CDH23\|12059\|AT | 0.414 | 0.630 | 0.000 | 0.217 |
| CDH23\|12060\|AT | 0.451 | 0.100 | 0.000 | -0.351 |
| MICU1\|12093\|AP | 0.232 | 0.535 | 0.000 | 0.303 |
| MICU1\|12094\|AP | 0.768 | 0.465 | 0.000 | -0.303 |
| PPP3CB\|12155\|ES | 0.395 | 0.500 | 0.002 | 0.106 |
| ADK\|12257\|AP | 0.823 | 0.609 | 0.000 | -0.214 |
| ADK\|12258\|AP | 0.177 | 0.382 | 0.000 | 0.204 |
| RPS24\|12295\|ES | 0.581 | 0.347 | 0.000 | -0.234 |
| RPS24\|12296\|ES | 0.830 | 0.565 | 0.000 | -0.265 |
| CCSER2\|12409\|ES | 0.358 | 0.252 | 0.002 | -0.106 |
| PAPSS2\|12457\|ES | 0.933 | 0.760 | 0.000 | -0.173 |
| PANK1\|12493\|AP | 0.108 | 0.225 | 0.000 | 0.117 |
| PANK1\|12494\|AP | 0.892 | 0.765 | 0.000 | -0.127 |
| FFAR4\|12553\|AT | 0.651 | 0.880 | 0.007 | 0.229 |
| FFAR4\|12554\|AT | 0.349 | 0.120 | 0.007 | -0.229 |
| SORBS1\|12641\|ES | 0.876 | 0.712 | 0.000 | -0.163 |
| ZDHHC16\|12704\|ES | 0.905 | 0.787 | 0.000 | -0.118 |
| MORN4\|12730\|AT | 0.180 | 0.293 | 0.000 | 0.113 |
| MORN4\|12731\|AT | 0.820 | 0.707 | 0.000 | -0.113 |
| ERLIN1\|12788\|RI | 0.283 | 0.102 | 0.000 | -0.181 |
| LDB1\|12934\|AP | 0.182 | 0.354 | 0.000 | 0.171 |
| LDB1\|12935\|AP | 0.818 | 0.638 | 0.000 | -0.179 |
| TMEM180\|12955\|ES | 0.864 | 0.725 | 0.000 | -0.139 |
| ADD3\|13077\|ES | 0.269 | 0.392 | 0.000 | 0.123 |
| ACSL5\|13108\|AP | 0.234 | 0.659 | 0.000 | 0.425 |
| ACSL5\|13111\|AP | 0.751 | 0.304 | 0.000 | -0.447 |
| VTI1A\|13115\|AT | 0.684 | 0.889 | 0.000 | 0.205 |
| VTI1A\|13116\|AT | 0.316 | 0.112 | 0.000 | -0.205 |
| BCCIP\|13431\|AT | 0.702 | 0.600 | 0.000 | -0.103 |
| SIGIRR\|13650\|AP | 0.516 | 0.815 | 0.000 | 0.299 |
| SIGIRR\|13651\|AP | 0.484 | 0.184 | 0.000 | -0.300 |
| SLC25A22\|13758\|AP | 0.935 | 0.834 | 0.000 | -0.101 |
| CHID1\|13801\|AP | 0.167 | 0.031 | 0.000 | -0.136 |
| CHID1\|13803\|AP | 0.597 | 0.111 | 0.000 | -0.486 |
| CHID1\|13804\|AP | 0.188 | 0.722 | 0.000 | 0.535 |
| IGF2\|13896\|AP | 0.059 | 0.213 | 0.000 | 0.155 |
| IGF2\|13898\|AP | 0.026 | 0.494 | 0.000 | 0.469 |
| IGF2\|13899\|AP | 0.907 | 0.006 | 0.000 | -0.901 |
| PRKCDBP\|14097\|ES | 0.324 | 0.219 | 0.020 | -0.105 |
| MPPED2\|14793\|AT | 0.501 | 0.704 | 0.000 | 0.202 |
| MPPED2\|14794\|AT | 0.499 | 0.296 | 0.000 | -0.202 |
| APIP\|14965\|AT | 0.833 | 0.974 | 0.000 | 0.141 |
| APIP\|14967\|AT | 0.167 | 0.026 | 0.000 | -0.141 |
| DGKZ\|15540\|AP | 0.489 | 0.612 | 0.001 | 0.122 |
| DGKZ\|15541\|AP | 0.404 | 0.282 | 0.002 | -0.122 |
| MDK\|15566\|AP | 0.544 | 0.806 | 0.000 | 0.262 |
| MDK\|15567\|AP | 0.456 | 0.194 | 0.000 | -0.262 |
| NR1H3\|15704\|RI | 0.487 | 0.602 | 0.016 | 0.116 |
| SLC43A1\|15844\|AP | 0.405 | 0.513 | 0.008 | 0.108 |
| GLYAT\|16016\|RI | 0.468 | 0.623 | 0.000 | 0.154 |
| DDB1\|16152\|AP | 0.696 | 0.837 | 0.016 | 0.141 |
| DDB1\|16155\|AP | 0.217 | 0.113 | 0.002 | -0.104 |
| CYB561A3\|16164\|AA | 0.784 | 0.898 | 0.000 | 0.114 |
| PPP1R32\|16239\|AP | 0.797 | 0.673 | 0.000 | -0.125 |
| PPP1R32\|16240\|AP | 0.199 | 0.308 | 0.000 | 0.110 |
| FADS1\|16298\|AP | 0.656 | 0.528 | 0.008 | -0.128 |
| AHNAK\|16347\|AT | 0.383 | 0.228 | 0.000 | -0.155 |
| AHNAK\|16348\|AT | 0.617 | 0.772 | 0.000 | 0.155 |
| BSCL2\|16400\|AP | 0.459 | 0.580 | 0.000 | 0.121 |
| BSCL2\|16403\|AP | 0.534 | 0.403 | 0.000 | -0.131 |
| SLC3A2\|16462\|AP | 0.062 | 0.163 | 0.000 | 0.102 |
| SLC3A2\|16464\|AP | 0.931 | 0.818 | 0.000 | -0.114 |
| PLA2G16\|16516\|AP | 0.445 | 0.564 | 0.000 | 0.119 |
| PLA2G16\|16517\|AP | 0.555 | 0.436 | 0.000 | -0.119 |
| VEGFB\|16601\|AA | 0.697 | 0.799 | 0.000 | 0.103 |
| BAD\|16616\|RI | 0.163 | 0.055 | 0.000 | -0.108 |
| LTBP3\|16862\|AP | 0.850 | 0.729 | 0.001 | -0.121 |
| LTBP3\|16865\|ES | 0.615 | 0.419 | 0.000 | -0.196 |
| EIF1AD\|16978\|AD | 0.775 | 0.661 | 0.008 | -0.114 |
| RBM14-RBM4\|17102\|ES | 0.957 | 0.659 | 0.000 | -0.298 |
| PC\|17134\|AP | 0.561 | 0.801 | 0.003 | 0.240 |
| PC\|17135\|AP | 0.439 | 0.199 | 0.003 | -0.240 |
| ANO1\|17387\|ES | 0.049 | 0.185 | 0.000 | 0.136 |
| NUMA1\|17513\|AP | 0.928 | 0.786 | 0.000 | -0.143 |
| NUMA1\|17514\|AP | 0.064 | 0.194 | 0.000 | 0.130 |
| NUMA1\|17515\|ES | 0.707 | 0.603 | 0.000 | -0.105 |
| ARAP1\|17639\|AP | 0.071 | 0.191 | 0.000 | 0.120 |
| ARAP1\|17640\|AP | 0.875 | 0.748 | 0.000 | -0.126 |
| SLCO2B1\|17818\|AP | 0.209 | 0.672 | 0.000 | 0.462 |
| SLCO2B1\|17819\|AP | 0.791 | 0.316 | 0.000 | -0.475 |
| UVRAG\|17878\|AP | 0.419 | 0.654 | 0.000 | 0.235 |
| UVRAG\|17880\|AP | 0.457 | 0.257 | 0.000 | -0.201 |
| PAK1\|17950\|AP | 0.356 | 0.199 | 0.000 | -0.157 |
| PAK1\|17951\|AP | 0.621 | 0.773 | 0.000 | 0.152 |
| TMEM126B\|18122\|ES | 0.808 | 0.695 | 0.000 | -0.113 |
| SMCO4\|18299\|AP | 0.219 | 0.525 | 0.000 | 0.306 |
| SMCO4\|18300\|AP | 0.782 | 0.475 | 0.000 | -0.306 |
| C11orf1\|18686\|AP | 0.370 | 0.475 | 0.000 | 0.105 |
| C11orf1\|18688\|AP | 0.621 | 0.513 | 0.000 | -0.107 |
| CRYAB\|18691\|AP | 0.297 | 0.141 | 0.000 | -0.157 |
| BCO2\|18749\|AT | 0.534 | 0.341 | 0.000 | -0.193 |
| BCO2\|18750\|AT | 0.466 | 0.659 | 0.000 | 0.193 |
| APOA1\|18869\|RI | 0.559 | 0.746 | 0.000 | 0.186 |
| APOA1\|18870\|RI | 0.172 | 0.342 | 0.000 | 0.170 |
| VWA5A\|19211\|AT | 0.421 | 0.531 | 0.000 | 0.110 |
| VWA5A\|19212\|AT | 0.579 | 0.469 | 0.000 | -0.110 |
| TBRG1\|19224\|ES | 0.450 | 0.339 | 0.000 | -0.111 |
| TBRG1\|19225\|ES | 0.788 | 0.663 | 0.000 | -0.126 |
| CHEK1\|19311\|AT | 0.273 | 0.472 | 0.000 | 0.199 |
| CHEK1\|19312\|AT | 0.727 | 0.528 | 0.000 | -0.199 |
| APLP2\|19479\|ES | 0.711 | 0.526 | 0.000 | -0.186 |
| SLC6A12\|19579\|AP | 0.325 | 0.592 | 0.000 | 0.267 |
| SLC6A12\|19580\|AP | 0.632 | 0.367 | 0.000 | -0.265 |
| ING4\|19914\|ES | 1.000 | 0.857 | 0.000 | -0.143 |
| LRRC23\|19994\|AP | 0.471 | 0.325 | 0.001 | -0.146 |
| LRRC23\|19997\|AP | 0.527 | 0.675 | 0.001 | 0.148 |
| PTPN6\|20022\|AP | 0.621 | 0.779 | 0.001 | 0.158 |
| PTPN6\|20023\|AP | 0.379 | 0.221 | 0.000 | -0.158 |
| PHB2\|20044\|ES | 0.413 | 0.614 | 0.000 | 0.201 |
| YBX3\|20481\|ES | 0.506 | 0.667 | 0.000 | 0.161 |
| SLCO1A2\|20676\|AP | 0.165 | 0.000 | 0.006 | -0.165 |
| SLCO1A2\|20677\|AP | 0.835 | 1.000 | 0.006 | 0.165 |
| FGFR1OP2\|20856\|ES | 0.617 | 0.498 | 0.002 | -0.119 |
| DNM1L\|21045\|ES | 0.355 | 0.462 | 0.001 | 0.106 |
| PPHLN1\|21227\|ES | 0.735 | 0.626 | 0.000 | -0.109 |
| ADCY6\|21464\|AP | 0.860 | 0.741 | 0.022 | -0.118 |
| ADCY6\|21465\|AP | 0.140 | 0.259 | 0.023 | 0.118 |
| TROAP\|21551\|AT | 0.680 | 0.266 | 0.000 | -0.414 |
| TROAP\|21552\|AT | 0.194 | 0.451 | 0.000 | 0.256 |
| TROAP\|21553\|AT | 0.132 | 0.245 | 0.000 | 0.113 |
| FAM186B\|21594\|AT | 0.466 | 0.360 | 0.000 | -0.106 |
| TMBIM6\|21613\|AD | 0.334 | 0.228 | 0.017 | -0.106 |
| RACGAP1\|21625\|AT | 0.139 | 0.015 | 0.008 | -0.123 |
| RACGAP1\|21628\|AT | 0.862 | 0.985 | 0.008 | 0.123 |
| LIMA1\|21688\|AP | 0.367 | 0.217 | 0.001 | -0.151 |
| LETMD1\|21759\|ES | 0.861 | 0.756 | 0.000 | -0.105 |
| NR4A1\|21884\|AP | 0.900 | 0.718 | 0.000 | -0.183 |
| NR4A1\|21887\|AP | 0.100 | 0.282 | 0.000 | 0.183 |
| PCBP2\|22052\|ES | 0.692 | 0.800 | 0.000 | 0.108 |
| CALCOCO1\|22108\|RI | 0.098 | 0.206 | 0.000 | 0.107 |
| ITGA7\|22209\|AP | 0.574 | 0.792 | 0.005 | 0.218 |
| ITGA7\|22210\|AP | 0.317 | 0.159 | 0.003 | -0.158 |
| ITGA7\|22216\|ES | 0.530 | 0.254 | 0.000 | -0.276 |
| SUOX\|22340\|ES | 0.583 | 0.746 | 0.000 | 0.164 |
| SMARCC2\|22393\|ES | 0.405 | 0.277 | 0.000 | -0.128 |
| SLC39A5\|22411\|AP | 0.857 | 0.747 | 0.000 | -0.109 |
| B4GALNT1\|22675\|AT | 0.545 | 0.696 | 0.000 | 0.151 |
| MON2\|22839\|ES | 0.434 | 0.570 | 0.000 | 0.136 |
| TMEM5\|22852\|ES | 0.446 | 0.294 | 0.000 | -0.153 |
| SRGAP1\|22854\|AP | 0.416 | 0.266 | 0.008 | -0.149 |
| SRGAP1\|22855\|AP | 0.572 | 0.727 | 0.009 | 0.154 |
| RAB3IP\|23342\|AP | 0.436 | 0.237 | 0.000 | -0.199 |
| RAB3IP\|23343\|AP | 0.335 | 0.526 | 0.000 | 0.191 |
| THAP2\|23408\|AT | 0.229 | 0.372 | 0.005 | 0.143 |
| THAP2\|23409\|AT | 0.771 | 0.628 | 0.005 | -0.143 |
| SOCS2\|23705\|AP | 0.611 | 0.450 | 0.000 | -0.162 |
| ANKS1B\|23885\|AT | 0.018 | 0.135 | 0.000 | 0.117 |
| ANKS1B\|23886\|AT | 0.982 | 0.847 | 0.000 | -0.135 |
| IGF1\|24050\|AT | 0.355 | 0.571 | 0.000 | 0.217 |
| IGF1\|24051\|AT | 0.645 | 0.429 | 0.000 | -0.217 |
| NFYB\|24093\|AP | 0.495 | 0.387 | 0.002 | -0.108 |
| NFYB\|24094\|AP | 0.505 | 0.613 | 0.002 | 0.108 |
| UNG\|24277\|AP | 0.019 | 0.136 | 0.000 | 0.118 |
| UNG\|24278\|AP | 0.981 | 0.864 | 0.000 | -0.118 |
| GIT2\|24385\|ES | 0.864 | 0.755 | 0.042 | -0.108 |
| VPS29\|24446\|ES | 0.089 | 0.256 | 0.000 | 0.166 |
| PRKAB1\|24709\|ES | 0.733 | 0.281 | 0.000 | -0.452 |
| RPLP0\|24731\|ES | 0.295 | 0.181 | 0.000 | -0.114 |
| WDR66\|24918\|AT | 0.481 | 0.336 | 0.001 | -0.145 |
| WDR66\|24919\|AT | 0.519 | 0.658 | 0.001 | 0.139 |
| ABCB9\|24994\|AP | 0.790 | 0.660 | 0.000 | -0.130 |
| OGFOD2\|25005\|AP | 0.105 | 0.220 | 0.000 | 0.115 |
| OGFOD2\|25006\|AP | 0.895 | 0.780 | 0.000 | -0.115 |
| NCOR2\|25143\|AD | 0.767 | 0.890 | 0.000 | 0.123 |
| AACS\|25173\|AP | 0.731 | 0.837 | 0.007 | 0.106 |
| PXMP2\|25289\|ES | 0.341 | 0.443 | 0.003 | 0.102 |
| PHF11\|25890\|AP | 0.249 | 0.129 | 0.000 | -0.120 |
| PHF11\|25891\|AP | 0.751 | 0.872 | 0.001 | 0.120 |
| UGGT2\|26129\|AT | 0.426 | 0.619 | 0.000 | 0.193 |
| UGGT2\|26131\|AT | 0.386 | 0.237 | 0.000 | -0.149 |
| NALCN\|26202\|AT | 0.755 | 0.451 | 0.000 | -0.304 |
| NALCN\|26204\|AT | 0.228 | 0.432 | 0.000 | 0.204 |
| MYO16\|26245\|AT | 0.427 | 0.241 | 0.003 | -0.187 |
| MYO16\|26246\|AT | 0.556 | 0.719 | 0.006 | 0.163 |
| CARKD\|26250\|AP | 0.571 | 0.288 | 0.000 | -0.283 |
| CARKD\|26251\|AP | 0.429 | 0.683 | 0.000 | 0.254 |
| MCF2L\|26317\|AP | 0.142 | 0.258 | 0.000 | 0.116 |
| MCF2L\|26320\|AP | 0.384 | 0.271 | 0.014 | -0.114 |
| F7\|26339\|ES | 0.296 | 0.547 | 0.000 | 0.251 |
| F7\|26344\|ES | 0.745 | 0.861 | 0.000 | 0.116 |
| TMEM55B\|26455\|AD | 0.291 | 0.417 | 0.000 | 0.126 |
| HNRNPC\|26552\|ES | 0.331 | 0.208 | 0.000 | -0.124 |
| HNRNPC\|26556\|ES | 0.389 | 0.268 | 0.000 | -0.121 |
| MRPL52\|26638\|ES | 0.312 | 0.430 | 0.001 | 0.118 |
| MRPL52\|26642\|RI | 0.358 | 0.559 | 0.004 | 0.201 |
| ACIN1\|26708\|ES | 0.850 | 0.732 | 0.000 | -0.118 |
| AP1G2\|26772\|RI | 0.505 | 0.695 | 0.011 | 0.190 |
| DCAF11\|26830\|AP | 0.812 | 0.598 | 0.000 | -0.213 |
| DCAF11\|26831\|AP | 0.188 | 0.377 | 0.000 | 0.189 |
| GMPR2\|26912\|AP | 0.386 | 0.565 | 0.000 | 0.179 |
| GMPR2\|26913\|AP | 0.614 | 0.435 | 0.000 | -0.179 |
| LTB4R\|26963\|AP | 0.646 | 0.540 | 0.005 | -0.107 |
| LTB4R\|26964\|AP | 0.354 | 0.460 | 0.005 | 0.107 |
| SDR39U1\|27005\|AP | 0.444 | 0.558 | 0.016 | 0.114 |
| CTAGE5\|27371\|AP | 0.303 | 0.132 | 0.000 | -0.171 |
| CTAGE5\|27373\|AP | 0.498 | 0.668 | 0.000 | 0.170 |
| KTN1\|27637\|ES | 0.208 | 0.333 | 0.000 | 0.125 |
| MAX\|27935\|RI | 0.600 | 0.710 | 0.039 | 0.110 |
| NUMB\|28288\|ES | 0.307 | 0.529 | 0.000 | 0.223 |
| PTGR2\|28317\|AD | 0.416 | 0.301 | 0.000 | -0.116 |
| ALDH6A1\|28366\|AP | 0.910 | 0.756 | 0.000 | -0.153 |
| ALDH6A1\|28367\|AP | 0.090 | 0.244 | 0.000 | 0.153 |
| GSTZ1\|28583\|AP | 0.747 | 0.079 | 0.000 | -0.668 |
| GSTZ1\|28584\|AP | 0.251 | 0.896 | 0.000 | 0.645 |
| FBLN5\|28893\|ES | 0.342 | 0.482 | 0.001 | 0.141 |
| PPP4R4\|29091\|AT | 0.980 | 0.877 | 0.000 | -0.103 |
| SERPINA1\|29103\|RI | 0.801 | 0.977 | 0.000 | 0.177 |
| SERPINA1\|29104\|RI | 0.639 | 0.961 | 0.000 | 0.323 |
| SERPINA1\|29114\|ES | 0.072 | 0.191 | 0.001 | 0.118 |
| SERPINA1\|29115\|AA | 0.435 | 0.898 | 0.000 | 0.462 |
| SERPINA1\|29122\|ES | 0.048 | 0.163 | 0.000 | 0.115 |
| SERPINA1\|29123\|AA | 0.338 | 0.854 | 0.000 | 0.516 |
| SERPINA1\|29130\|ES | 0.657 | 0.940 | 0.000 | 0.284 |
| SERPINA1\|29132\|ES | 0.739 | 0.541 | 0.000 | -0.199 |
| SERPINA1\|29133\|ES | 0.151 | 0.000 | 0.046 | -0.151 |
| WARS\|29272\|AP | 0.218 | 0.115 | 0.000 | -0.103 |
| XRCC3\|29495\|AT | 0.300 | 0.173 | 0.000 | -0.126 |
| XRCC3\|29496\|AT | 0.700 | 0.827 | 0.000 | 0.126 |
| C14orf2\|29533\|ES | 0.329 | 0.454 | 0.000 | 0.125 |
| ASPG\|29539\|ES | 0.915 | 0.786 | 0.000 | -0.129 |
| C14orf80\|29661\|ES | 0.528 | 0.354 | 0.000 | -0.174 |
| C14orf80\|29662\|ES | 0.770 | 0.667 | 0.001 | -0.103 |
| NIPA2\|29683\|ES | 0.768 | 0.619 | 0.000 | -0.149 |
| NIPA2\|29684\|ES | 0.707 | 0.541 | 0.000 | -0.166 |
| NIPA2\|29685\|ES | 0.652 | 0.539 | 0.000 | -0.112 |
| DUT\|30484\|AP | 0.899 | 0.771 | 0.000 | -0.128 |
| DUT\|30485\|AP | 0.086 | 0.205 | 0.000 | 0.119 |
| GALK2\|30516\|AP | 0.650 | 0.510 | 0.000 | -0.141 |
| GALK2\|30518\|AP | 0.345 | 0.474 | 0.000 | 0.130 |
| TEX9\|30756\|AT | 0.378 | 0.539 | 0.013 | 0.161 |
| TEX9\|30759\|AT | 0.609 | 0.423 | 0.014 | -0.186 |
| LIPC\|30893\|AP | 0.369 | 0.261 | 0.041 | -0.108 |
| LIPC\|30897\|ES | 0.807 | 0.966 | 0.000 | 0.158 |
| SMAD6\|31294\|AP | 0.207 | 0.420 | 0.000 | 0.213 |
| SMAD6\|31295\|AP | 0.793 | 0.580 | 0.000 | -0.213 |
| CLK3\|31725\|AP | 1.000 | 0.740 | 0.004 | -0.260 |
| CLK3\|31726\|AP | 0.000 | 0.253 | 0.003 | 0.253 |
| NEIL1\|31857\|ES | 0.141 | 0.243 | 0.000 | 0.101 |
| NRG4\|31911\|AT | 0.170 | 0.000 | 0.000 | -0.170 |
| NRG4\|31913\|AT | 0.723 | 0.969 | 0.000 | 0.246 |
| FAH\|32179\|RI | 0.838 | 0.613 | 0.000 | -0.225 |
| FAH\|32180\|RI | 0.917 | 0.680 | 0.000 | -0.236 |
| FAM154B\|32219\|AT | 0.446 | 0.334 | 0.003 | -0.113 |
| ZSCAN2\|32289\|AT | 0.453 | 0.565 | 0.001 | 0.113 |
| ACAN\|32395\|AT | 0.378 | 0.662 | 0.000 | 0.284 |
| ACAN\|32396\|AT | 0.632 | 0.335 | 0.000 | -0.297 |
| LRRC28\|32712\|AD | 0.241 | 0.094 | 0.000 | -0.147 |
| TM2D3\|32771\|ES | 0.322 | 0.441 | 0.000 | 0.119 |
| PIGQ\|32903\|ES | 0.645 | 0.494 | 0.001 | -0.151 |
| C16orf13\|32923\|ES | 0.839 | 0.679 | 0.000 | -0.160 |
| FAM173A\|32964\|AA | 0.252 | 0.396 | 0.003 | 0.143 |
| SPSB3\|33131\|AP | 1.000 | 0.897 | 0.005 | -0.103 |
| SPSB3\|33132\|AP | 0.000 | 0.103 | 0.005 | 0.103 |
| MLST8\|33211\|AP | 0.401 | 0.538 | 0.000 | 0.137 |
| MLST8\|33212\|AP | 0.599 | 0.462 | 0.000 | -0.137 |
| PKMYT1\|33330\|AA | 0.893 | 0.636 | 0.000 | -0.257 |
| NAA60\|33527\|RI | 0.712 | 0.524 | 0.000 | -0.188 |
| UBALD1\|33765\|RI | 0.549 | 0.667 | 0.049 | 0.118 |
| EMP2\|33986\|AP | 0.266 | 0.538 | 0.000 | 0.272 |
| EMP2\|33987\|AP | 0.734 | 0.462 | 0.000 | -0.272 |
| SNX29\|34058\|ES | 0.990 | 0.694 | 0.000 | -0.296 |
| NPIPA5\|34149\|AD | 0.215 | 0.328 | 0.000 | 0.112 |
| NPIPA5\|34150\|ES | 0.391 | 0.542 | 0.000 | 0.151 |
| ABCC6\|34219\|AT | 0.298 | 0.543 | 0.000 | 0.246 |
| ABCC6\|34220\|AT | 0.702 | 0.457 | 0.000 | -0.246 |
| SYT17\|34292\|AP | 0.268 | 0.371 | 0.012 | 0.104 |
| SYT17\|34293\|AT | 0.775 | 0.672 | 0.001 | -0.104 |
| SYT17\|34294\|AT | 0.225 | 0.328 | 0.001 | 0.104 |
| ACSM2B\|34359\|AA | 0.533 | 0.710 | 0.000 | 0.176 |
| ACSM2B\|34361\|ES | 0.765 | 0.609 | 0.001 | -0.156 |
| LYRM1\|34403\|AP | 0.741 | 0.502 | 0.000 | -0.238 |
| LYRM1\|34404\|AP | 0.110 | 0.345 | 0.000 | 0.235 |
| TMEM159\|34426\|ES | 0.390 | 0.515 | 0.023 | 0.125 |
| NPIPB4\|35507\|AD | 0.805 | 0.920 | 0.000 | 0.115 |
| SULT1A2\|35764\|RI | 0.620 | 0.800 | 0.000 | 0.180 |
| SULT1A2\|35777\|ES | 0.564 | 0.452 | 0.000 | -0.111 |
| SULT1A2\|35779\|ES | 0.040 | 0.144 | 0.000 | 0.105 |
| SULT1A2\|35782\|ES | 0.172 | 0.276 | 0.000 | 0.104 |
| SULT1A2\|35791\|ES | 0.706 | 0.458 | 0.000 | -0.248 |
| SULT1A2\|35810\|ES | 0.475 | 0.367 | 0.001 | -0.109 |
| SULT1A2\|35811\|RI | 0.048 | 0.225 | 0.000 | 0.177 |
| KIF22\|35935\|AD | 0.214 | 0.065 | 0.000 | -0.149 |
| ASPHD1\|35983\|AT | 0.429 | 0.315 | 0.009 | -0.114 |
| ASPHD1\|35984\|AT | 0.514 | 0.678 | 0.004 | 0.164 |
| INO80E\|36009\|ES | 0.424 | 0.268 | 0.011 | -0.156 |
| ZNF688\|36160\|AD | 0.936 | 0.737 | 0.000 | -0.198 |
| ORAI3\|36207\|AT | 0.579 | 0.685 | 0.000 | 0.106 |
| ORAI3\|36208\|AT | 0.422 | 0.315 | 0.000 | -0.106 |
| ZNF720\|36283\|AT | 0.776 | 0.657 | 0.003 | -0.118 |
| ZNF720\|36284\|AT | 0.224 | 0.343 | 0.003 | 0.118 |
| GNAO1\|36460\|AT | 0.515 | 0.248 | 0.000 | -0.267 |
| GNAO1\|36461\|AT | 0.485 | 0.752 | 0.000 | 0.267 |
| BEAN1\|36708\|AT | 0.182 | 0.293 | 0.006 | 0.111 |
| BEAN1\|36710\|AT | 0.757 | 0.652 | 0.006 | -0.105 |
| CES3\|36906\|AP | 0.939 | 0.817 | 0.000 | -0.122 |
| NOL3\|36955\|ES | 0.571 | 0.684 | 0.000 | 0.113 |
| LRRC36\|37014\|AT | 0.152 | 0.286 | 0.011 | 0.134 |
| PLA2G15\|37203\|ES | 0.386 | 0.274 | 0.018 | -0.112 |
| PDPR\|37327\|AP | 0.534 | 0.639 | 0.001 | 0.105 |
| PDPR\|37329\|AP | 0.458 | 0.353 | 0.001 | -0.106 |
| MTSS1L\|37410\|ES | 0.702 | 0.522 | 0.000 | -0.180 |
| CMC2\|37721\|ES | 0.568 | 0.330 | 0.000 | -0.238 |
| CMC2\|37726\|ES | 0.781 | 0.554 | 0.000 | -0.227 |
| CENPN\|37739\|AT | 0.688 | 0.472 | 0.000 | -0.217 |
| CENPN\|37740\|AT | 0.286 | 0.528 | 0.000 | 0.242 |
| PKD1L2\|37759\|AT | 0.406 | 0.245 | 0.003 | -0.161 |
| COX4I1\|37903\|RI | 0.663 | 0.767 | 0.001 | 0.104 |
| SPATA33\|38106\|AT | 0.417 | 0.535 | 0.000 | 0.118 |
| SPATA33\|38107\|AT | 0.583 | 0.465 | 0.000 | -0.118 |
| CDK10\|38115\|RI | 0.150 | 0.254 | 0.000 | 0.104 |
| TUBB3\|38166\|AP | 0.354 | 0.205 | 0.008 | -0.150 |
| DBNDD1\|38196\|AP | 0.843 | 0.650 | 0.000 | -0.193 |
| DBNDD1\|38197\|AP | 0.083 | 0.184 | 0.000 | 0.102 |
| ENO3\|38619\|AT | 0.035 | 0.231 | 0.000 | 0.195 |
| ENO3\|38620\|AT | 0.965 | 0.769 | 0.000 | -0.195 |
| SCIMP\|38666\|AT | 0.296 | 0.482 | 0.000 | 0.186 |
| SCIMP\|38667\|AT | 0.704 | 0.518 | 0.000 | -0.186 |
| RPAIN\|38694\|ES | 0.617 | 0.447 | 0.000 | -0.171 |
| DERL2\|38705\|RI | 0.813 | 0.705 | 0.036 | -0.108 |
| ASGR2\|38835\|AA | 0.174 | 0.322 | 0.000 | 0.148 |
| GABARAP\|38867\|AD | 0.658 | 0.844 | 0.000 | 0.186 |
| ELP5\|38887\|AT | 0.188 | 0.073 | 0.000 | -0.115 |
| ELP5\|38888\|AT | 0.812 | 0.927 | 0.000 | 0.115 |
| CLDN7\|38892\|AP | 1.000 | 0.780 | 0.000 | -0.220 |
| CLDN7\|38894\|AP | 0.000 | 0.217 | 0.000 | 0.217 |
| DNAH2\|39052\|AT | 0.408 | 0.197 | 0.000 | -0.211 |
| DNAH2\|39053\|AT | 0.604 | 0.803 | 0.000 | 0.199 |
| TMEM88\|39057\|AA | 0.834 | 0.722 | 0.001 | -0.112 |
| TMEM107\|39128\|ES | 0.516 | 0.368 | 0.000 | -0.148 |
| TMEM107\|39129\|ES | 0.730 | 0.486 | 0.000 | -0.245 |
| RANGRF\|39163\|RI | 0.384 | 0.514 | 0.000 | 0.130 |
| NDEL1\|39189\|ES | 0.447 | 0.340 | 0.000 | -0.107 |
| TNFRSF13B\|39449\|AT | 0.289 | 0.133 | 0.000 | -0.156 |
| TNFRSF13B\|39450\|AT | 0.711 | 0.863 | 0.000 | 0.152 |
| PEMT\|39486\|AP | 0.082 | 0.507 | 0.000 | 0.425 |
| PEMT\|39487\|AP | 0.913 | 0.493 | 0.000 | -0.420 |
| MYO15A\|39574\|AP | 0.132 | 0.000 | 0.003 | -0.132 |
| TRIM16L\|39629\|AP | 0.791 | 0.419 | 0.000 | -0.372 |
| TRIM16L\|39631\|AP | 0.209 | 0.531 | 0.000 | 0.322 |
| TRIM16L\|39638\|ES | 1.000 | 0.869 | 0.006 | -0.131 |
| SPECC1\|39790\|AT | 0.405 | 0.515 | 0.000 | 0.110 |
| SPECC1\|39792\|AT | 0.595 | 0.485 | 0.000 | -0.110 |
| TLCD1\|39968\|AP | 0.710 | 0.853 | 0.000 | 0.142 |
| TLCD1\|39969\|AP | 0.290 | 0.147 | 0.000 | -0.142 |
| RHOT1\|40187\|ES | 0.497 | 0.382 | 0.008 | -0.115 |
| AP2B1\|40319\|ES | 0.645 | 0.541 | 0.000 | -0.104 |
| RDM1\|40348\|AT | 0.293 | 0.447 | 0.000 | 0.153 |
| RDM1\|40349\|AT | 0.636 | 0.517 | 0.000 | -0.119 |
| CCL14\|40366\|AP | 0.515 | 0.657 | 0.000 | 0.142 |
| CCL14\|40367\|AP | 0.485 | 0.343 | 0.000 | -0.142 |
| CCL14\|40370\|RI | 0.187 | 0.389 | 0.000 | 0.202 |
| CCL14\|40381\|RI | 0.087 | 0.239 | 0.000 | 0.152 |
| MYO19\|40482\|AA | 0.401 | 0.278 | 0.000 | -0.123 |
| TBC1D3F\|40558\|AA | 0.350 | 0.458 | 0.018 | 0.108 |
| PLXDC1\|40623\|ES | 0.379 | 0.275 | 0.000 | -0.104 |
| THRA\|40839\|AT | 0.714 | 0.601 | 0.000 | -0.113 |
| THRA\|40840\|AT | 0.286 | 0.399 | 0.000 | 0.113 |
| COASY\|41068\|RI | 0.590 | 0.776 | 0.000 | 0.186 |
| TMUB2\|41814\|ES | 0.690 | 0.585 | 0.002 | -0.105 |
| SLC25A39\|41837\|AA | 0.395 | 0.283 | 0.000 | -0.113 |
| HEXIM2\|41949\|AP | 0.543 | 0.398 | 0.000 | -0.145 |
| HEXIM2\|41950\|AP | 0.457 | 0.602 | 0.000 | 0.145 |
| SCRN2\|42120\|RI | 0.241 | 0.367 | 0.000 | 0.126 |
| HLF\|42576\|AP | 0.271 | 0.585 | 0.000 | 0.314 |
| HLF\|42579\|AP | 0.715 | 0.345 | 0.000 | -0.369 |
| BCAS3\|42864\|AP | 0.406 | 0.269 | 0.001 | -0.137 |
| BCAS3\|42865\|AP | 0.595 | 0.731 | 0.001 | 0.136 |
| ICAM2\|43038\|AP | 0.162 | 0.262 | 0.000 | 0.101 |
| ICAM2\|43039\|AP | 0.838 | 0.738 | 0.000 | -0.101 |
| PRKAR1A\|43141\|AP | 0.348 | 0.236 | 0.008 | -0.112 |
| EXOC7\|43569\|ES | 0.312 | 0.486 | 0.000 | 0.174 |
| EXOC7\|43572\|ES | 0.455 | 0.271 | 0.000 | -0.184 |
| CYGB\|43589\|AP | 0.587 | 0.427 | 0.001 | -0.160 |
| CYGB\|43591\|AP | 0.400 | 0.573 | 0.001 | 0.173 |
| SEC14L1\|43703\|AP | 0.212 | 0.339 | 0.000 | 0.127 |
| SEPT9\|43716\|AP | 0.347 | 0.448 | 0.002 | 0.101 |
| SEPT9\|43726\|AP | 0.359 | 0.188 | 0.000 | -0.171 |
| AFMID\|43794\|ES | 0.739 | 0.405 | 0.000 | -0.333 |
| AFMID\|43795\|ES | 0.901 | 0.653 | 0.000 | -0.249 |
| AFMID\|43796\|ES | 0.333 | 0.185 | 0.000 | -0.148 |
| AFMID\|43800\|ES | 0.892 | 0.711 | 0.000 | -0.181 |
| AFMID\|43801\|ES | 0.431 | 0.188 | 0.000 | -0.242 |
| AFMID\|43804\|ES | 0.740 | 0.398 | 0.000 | -0.342 |
| AFMID\|43811\|ES | 0.067 | 0.199 | 0.000 | 0.133 |
| AFMID\|43814\|ES | 0.931 | 0.762 | 0.000 | -0.168 |
| AFMID\|43824\|ES | 0.643 | 0.317 | 0.000 | -0.327 |
| AFMID\|43829\|ES | 0.489 | 0.362 | 0.002 | -0.128 |
| AFMID\|43832\|ES | 0.761 | 0.526 | 0.000 | -0.235 |
| AFMID\|43844\|ES | 0.664 | 0.783 | 0.001 | 0.119 |
| CANT1\|43973\|RI | 0.516 | 0.397 | 0.004 | -0.120 |
| C1QTNF1\|43983\|AP | 0.433 | 0.616 | 0.000 | 0.183 |
| C1QTNF1\|43985\|AP | 0.376 | 0.269 | 0.000 | -0.107 |
| CARD14\|44027\|AT | 0.564 | 0.454 | 0.013 | -0.110 |
| ENDOV\|44053\|AT | 0.340 | 0.466 | 0.000 | 0.127 |
| ENDOV\|44054\|AT | 0.627 | 0.483 | 0.000 | -0.145 |
| BAIAP2\|44096\|ES | 0.616 | 0.379 | 0.000 | -0.237 |
| C17orf70\|44129\|AD | 0.251 | 0.361 | 0.000 | 0.110 |
| OXLD1\|44143\|AD | 0.400 | 0.553 | 0.000 | 0.153 |
| PCYT2\|44230\|ES | 0.521 | 0.357 | 0.000 | -0.164 |
| NARF\|44391\|AP | 0.261 | 0.133 | 0.000 | -0.128 |
| NARF\|44392\|AP | 0.740 | 0.867 | 0.000 | 0.128 |
| ENOSF1\|44462\|AT | 0.294 | 0.394 | 0.000 | 0.100 |
| ENOSF1\|44464\|AT | 0.706 | 0.606 | 0.000 | -0.100 |
| MYOM1\|44486\|ES | 0.358 | 0.699 | 0.000 | 0.342 |
| MYL12A\|44491\|ES | 0.151 | 0.269 | 0.000 | 0.118 |
| RBBP8\|44784\|AP | 0.848 | 0.732 | 0.000 | -0.116 |
| ANKRD29\|44841\|AT | 0.854 | 0.672 | 0.000 | -0.182 |
| ANKRD29\|44842\|AT | 0.146 | 0.328 | 0.000 | 0.182 |
| OSBPL1A\|44876\|AP | 0.293 | 0.406 | 0.004 | 0.113 |
| OSBPL1A\|44877\|AP | 0.694 | 0.521 | 0.004 | -0.173 |
| SS18\|44906\|AP | 0.345 | 0.203 | 0.000 | -0.142 |
| MAPRE2\|45135\|AP | 0.962 | 0.862 | 0.000 | -0.100 |
| ZNF397\|45144\|AT | 0.518 | 0.392 | 0.000 | -0.126 |
| ZNF397\|45145\|AT | 0.248 | 0.364 | 0.000 | 0.116 |
| RNF165\|45394\|AT | 0.234 | 0.605 | 0.000 | 0.371 |
| RNF165\|45395\|AT | 0.766 | 0.395 | 0.000 | -0.371 |
| NEDD4L\|45674\|ES | 0.796 | 0.902 | 0.038 | 0.106 |
| KIAA1468\|45699\|ME | 0.534 | 0.636 | 0.006 | 0.101 |
| ZCCHC2\|45702\|AP | 0.644 | 0.928 | 0.000 | 0.285 |
| ZCCHC2\|45703\|AP | 0.356 | 0.072 | 0.000 | -0.285 |
| SERPINB5\|45716\|AT | 0.160 | 0.048 | 0.000 | -0.113 |
| PQLC1\|46257\|ES | 0.302 | 0.124 | 0.000 | -0.178 |
| TXNL4A\|46282\|ES | 0.790 | 0.614 | 0.000 | -0.176 |
| PARD6G\|46292\|AT | 0.823 | 0.572 | 0.000 | -0.251 |
| PARD6G\|46293\|AT | 0.177 | 0.428 | 0.000 | 0.251 |
| CIRBP\|46426\|RI | 0.238 | 0.388 | 0.001 | 0.150 |
| C19orf24\|46446\|AP | 0.331 | 0.432 | 0.000 | 0.101 |
| C19orf24\|46447\|AP | 0.669 | 0.568 | 0.000 | -0.101 |
| MUM1\|46457\|ES | 0.194 | 0.328 | 0.000 | 0.134 |
| ATP8B3\|46543\|AT | 0.732 | 0.909 | 0.000 | 0.177 |
| ATP8B3\|46544\|AT | 0.268 | 0.091 | 0.000 | -0.177 |
| INSR\|47099\|ES | 0.906 | 0.792 | 0.000 | -0.114 |
| SNAPC2\|47195\|AP | 0.333 | 0.176 | 0.000 | -0.157 |
| SNAPC2\|47196\|AP | 0.682 | 0.820 | 0.000 | 0.138 |
| C19orf66\|47446\|AP | 0.275 | 0.119 | 0.000 | -0.156 |
| C19orf66\|47447\|AP | 0.725 | 0.881 | 0.000 | 0.156 |
| C19orf66\|47451\|RI | 0.321 | 0.507 | 0.000 | 0.186 |
| ICAM3\|47503\|RI | 0.156 | 0.439 | 0.000 | 0.282 |
| ACP5\|47745\|AP | 0.159 | 0.057 | 0.000 | -0.102 |
| HOOK2\|47862\|AP | 0.082 | 0.207 | 0.000 | 0.125 |
| HOOK2\|47863\|AP | 0.918 | 0.797 | 0.000 | -0.121 |
| ZNF333\|48017\|AT | 0.351 | 0.195 | 0.000 | -0.156 |
| AKAP8L\|48076\|ES | 0.331 | 0.199 | 0.000 | -0.132 |
| WIZ\|48090\|ES | 0.800 | 0.694 | 0.003 | -0.106 |
| CYP4F12\|48110\|RI | 0.054 | 0.170 | 0.000 | 0.115 |
| TPM4\|48124\|AP | 0.416 | 0.083 | 0.000 | -0.333 |
| TPM4\|48125\|AP | 0.584 | 0.917 | 0.000 | 0.333 |
| SSBP4\|48426\|AP | 0.844 | 0.953 | 0.000 | 0.108 |
| SSBP4\|48427\|AP | 0.156 | 0.047 | 0.000 | -0.108 |
| COPE\|48519\|ES | 0.742 | 0.601 | 0.003 | -0.141 |
| ZNF101\|48677\|RI | 0.929 | 0.638 | 0.000 | -0.291 |
| ZNF506\|48680\|AT | 0.474 | 0.366 | 0.000 | -0.108 |
| ZNF737\|48720\|AT | 1.000 | 0.878 | 0.030 | -0.122 |
| ZNF737\|48721\|AT | 0.000 | 0.122 | 0.030 | 0.122 |
| ZNF626\|48723\|AT | 0.702 | 0.594 | 0.004 | -0.107 |
| ZNF626\|48724\|AT | 0.298 | 0.406 | 0.004 | 0.107 |
| ZNF85\|48732\|AP | 0.691 | 0.549 | 0.008 | -0.143 |
| ZNF85\|48733\|AP | 0.306 | 0.451 | 0.008 | 0.145 |
| ZNF738\|48763\|AT | 0.673 | 0.512 | 0.001 | -0.161 |
| ZNF208\|48799\|AT | 0.066 | 0.222 | 0.016 | 0.156 |
| ZNF675\|48823\|AT | 0.272 | 0.160 | 0.023 | -0.112 |
| ZNF726\|48832\|AT | 0.537 | 0.355 | 0.007 | -0.182 |
| FXYD1\|49055\|AP | 0.389 | 0.182 | 0.000 | -0.207 |
| FXYD1\|49056\|AP | 0.426 | 0.635 | 0.000 | 0.209 |
| WDR62\|49337\|RI | 1.000 | 0.787 | 0.000 | -0.213 |
| WDR62\|49339\|RI | 0.763 | 0.521 | 0.000 | -0.242 |
| ZNF567\|49414\|AP | 0.294 | 0.591 | 0.000 | 0.297 |
| ZNF567\|49415\|AP | 0.579 | 0.372 | 0.000 | -0.207 |
| ZNF527\|49506\|AT | 0.880 | 0.772 | 0.007 | -0.108 |
| ZNF527\|49507\|AT | 0.120 | 0.228 | 0.007 | 0.108 |
| TMEM91\|50052\|RI | 0.492 | 0.662 | 0.002 | 0.170 |
| TMEM91\|50058\|RI | 0.439 | 0.558 | 0.025 | 0.119 |
| TMEM145\|50151\|AT | 0.000 | 0.308 | 0.000 | 0.308 |
| TMEM145\|50152\|AT | 1.000 | 0.692 | 0.000 | -0.308 |
| CEACAM1\|50162\|ES | 0.195 | 0.488 | 0.000 | 0.293 |
| ZNF226\|50290\|RI | 0.525 | 0.344 | 0.000 | -0.182 |
| ZNF226\|50291\|AA | 0.938 | 0.797 | 0.000 | -0.141 |
| ZNF235\|50303\|AT | 0.646 | 0.532 | 0.000 | -0.114 |
| APOC1\|50356\|AP | 0.655 | 0.784 | 0.002 | 0.130 |
| APOC1\|50357\|AP | 0.345 | 0.216 | 0.002 | -0.130 |
| ERCC1\|50438\|AP | 0.478 | 0.639 | 0.012 | 0.162 |
| ERCC1\|50439\|AP | 0.505 | 0.354 | 0.014 | -0.151 |
| OPA3\|50488\|AT | 0.285 | 0.177 | 0.000 | -0.108 |
| OPA3\|50489\|AT | 0.715 | 0.823 | 0.000 | 0.108 |
| SYMPK\|50529\|AP | 0.361 | 0.558 | 0.000 | 0.197 |
| SYMPK\|50530\|AP | 0.639 | 0.442 | 0.000 | -0.197 |
| PTGIR\|50569\|AT | 0.870 | 0.674 | 0.000 | -0.196 |
| PTGIR\|50570\|AT | 0.130 | 0.326 | 0.000 | 0.196 |
| DACT3\|50576\|AT | 0.398 | 0.499 | 0.002 | 0.100 |
| DACT3\|50577\|AT | 0.602 | 0.501 | 0.002 | -0.100 |
| BCAT2\|50810\|AP | 0.265 | 0.808 | 0.000 | 0.543 |
| BCAT2\|50811\|AP | 0.735 | 0.192 | 0.000 | -0.543 |
| BCAT2\|50816\|ES | 0.828 | 0.391 | 0.000 | -0.436 |
| IRF3\|51010\|ES | 1.000 | 0.873 | 0.000 | -0.127 |
| AKT1S1\|51110\|AP | 0.786 | 0.672 | 0.006 | -0.114 |
| NUP62\|51127\|RI | 0.829 | 0.713 | 0.000 | -0.116 |
| ZNF577\|51381\|RI | 0.807 | 1.000 | 0.046 | 0.193 |
| ZNF83\|51479\|AP | 0.000 | 0.205 | 0.000 | 0.205 |
| ZNF331\|51722\|AP | 0.068 | 0.328 | 0.000 | 0.260 |
| ZNF331\|51724\|AP | 0.278 | 0.051 | 0.000 | -0.228 |
| RPS9\|51823\|ES | 0.157 | 0.328 | 0.000 | 0.171 |
| RPS9\|51824\|ES | 0.125 | 0.304 | 0.000 | 0.179 |
| RPS9\|51825\|ES | 0.596 | 0.793 | 0.000 | 0.197 |
| ISOC2\|52105\|ES | 0.901 | 0.640 | 0.000 | -0.261 |
| CCDC106\|52130\|RI | 0.370 | 0.269 | 0.000 | -0.101 |
| ZNF530\|52304\|ES | 1.000 | 0.660 | 0.002 | -0.340 |
| ZNF814\|52354\|AT | 0.514 | 0.367 | 0.005 | -0.148 |
| ZNF606\|52380\|AT | 0.554 | 0.435 | 0.000 | -0.120 |
| ZNF446\|52471\|AD | 0.438 | 0.333 | 0.003 | -0.105 |
| SH3YL1\|52493\|AP | 0.545 | 0.158 | 0.000 | -0.386 |
| SH3YL1\|52495\|AP | 0.126 | 0.323 | 0.000 | 0.197 |
| ACP1\|52514\|ES | 0.539 | 0.436 | 0.000 | -0.103 |
| ITGB1BP1\|52612\|AP | 0.490 | 0.384 | 0.000 | -0.106 |
| ITGB1BP1\|52613\|AP | 0.510 | 0.616 | 0.000 | 0.106 |
| ITGB1BP1\|52617\|AA | 0.642 | 0.454 | 0.000 | -0.188 |
| EFR3B\|52847\|AT | 0.408 | 0.520 | 0.001 | 0.111 |
| KHK\|52929\|ME | 0.061 | 0.198 | 0.000 | 0.137 |
| ATL2\|53248\|ES | 0.192 | 0.000 | 0.001 | -0.192 |
| ABCG5\|53408\|RI | 0.356 | 0.525 | 0.000 | 0.169 |
| SLC3A1\|53419\|AP | 0.345 | 0.802 | 0.000 | 0.457 |
| SLC3A1\|53420\|AP | 0.655 | 0.198 | 0.000 | -0.457 |
| PRKCE\|53460\|AT | 0.780 | 0.899 | 0.000 | 0.119 |
| PRKCE\|53461\|AT | 0.220 | 0.102 | 0.000 | -0.119 |
| MCFD2\|53478\|ES | 0.361 | 0.242 | 0.000 | -0.118 |
| PUS10\|53672\|AP | 1.000 | 0.890 | 0.000 | -0.110 |
| PUS10\|53673\|AP | 0.000 | 0.108 | 0.000 | 0.108 |
| EHBP1\|53718\|ES | 0.253 | 0.470 | 0.000 | 0.217 |
| UGP2\|53744\|AP | 0.129 | 0.366 | 0.000 | 0.238 |
| UGP2\|53745\|AP | 0.870 | 0.628 | 0.000 | -0.242 |
| MEIS1\|53804\|AP | 0.810 | 0.922 | 0.000 | 0.112 |
| MEIS1\|53805\|AP | 0.172 | 0.066 | 0.000 | -0.106 |
| DGUOK\|54003\|ES | 0.676 | 0.552 | 0.000 | -0.123 |
| DGUOK\|54006\|ES | 0.859 | 0.733 | 0.000 | -0.125 |
| DGUOK\|54008\|ES | 0.815 | 0.699 | 0.000 | -0.116 |
| DGUOK\|54009\|ES | 0.448 | 0.282 | 0.000 | -0.166 |
| THNSL2\|54465\|AP | 0.695 | 0.826 | 0.000 | 0.131 |
| THNSL2\|54466\|AP | 0.305 | 0.168 | 0.000 | -0.136 |
| THNSL2\|54471\|ES | 0.884 | 0.746 | 0.000 | -0.139 |
| IL1R1\|54771\|AP | 0.242 | 0.371 | 0.000 | 0.128 |
| IL1R1\|54773\|AP | 0.670 | 0.382 | 0.000 | -0.288 |
| ST6GAL2\|54866\|AT | 0.809 | 0.501 | 0.000 | -0.308 |
| ST6GAL2\|54867\|AT | 0.191 | 0.500 | 0.000 | 0.308 |
| CBWD2\|55054\|ES | 0.325 | 0.547 | 0.004 | 0.222 |
| STEAP3\|55101\|ES | 0.210 | 0.341 | 0.000 | 0.131 |
| BIN1\|55200\|ES | 0.267 | 0.538 | 0.000 | 0.271 |
| ARHGEF4\|55355\|AT | 0.219 | 0.049 | 0.044 | -0.170 |
| ARHGEF4\|55356\|AT | 0.781 | 0.951 | 0.044 | 0.170 |
| RIF1\|55572\|ES | 0.740 | 0.579 | 0.000 | -0.161 |
| NR4A2\|55613\|AP | 0.121 | 0.531 | 0.000 | 0.409 |
| NR4A2\|55614\|AP | 0.857 | 0.438 | 0.000 | -0.418 |
| CCDC148\|55662\|AT | 0.335 | 0.191 | 0.003 | -0.144 |
| ITGA6\|55968\|ES | 0.581 | 0.450 | 0.000 | -0.130 |
| CCDC141\|56396\|AT | 0.177 | 0.322 | 0.000 | 0.145 |
| CCDC141\|56398\|AT | 0.705 | 0.471 | 0.000 | -0.234 |
| SSFA2\|56440\|AP | 0.156 | 0.013 | 0.000 | -0.143 |
| GULP1\|56499\|AT | 0.408 | 0.592 | 0.020 | 0.184 |
| GULP1\|56500\|AT | 0.592 | 0.408 | 0.020 | -0.184 |
| GLS\|56589\|AT | 0.440 | 0.599 | 0.000 | 0.158 |
| GLS\|56590\|AT | 0.560 | 0.401 | 0.000 | -0.158 |
| MYO1B\|56608\|ES | 0.311 | 0.524 | 0.000 | 0.213 |
| NABP1\|56614\|ES | 0.328 | 0.494 | 0.000 | 0.166 |
| ANKRD44\|56670\|AT | 0.669 | 0.502 | 0.003 | -0.167 |
| ANKRD44\|56671\|AT | 0.321 | 0.431 | 0.004 | 0.111 |
| FN1\|57392\|AA | 0.687 | 0.867 | 0.000 | 0.180 |
| FN1\|57393\|AA | 0.267 | 0.634 | 0.000 | 0.367 |
| FN1\|57395\|ES | 0.703 | 0.902 | 0.000 | 0.199 |
| FN1\|57397\|ES | 0.045 | 0.279 | 0.000 | 0.234 |
| ATG9A\|57634\|AP | 0.586 | 0.458 | 0.000 | -0.128 |
| ATG9A\|57635\|AP | 0.414 | 0.542 | 0.000 | 0.128 |
| GMPPA\|57715\|AD | 0.232 | 0.362 | 0.000 | 0.130 |
| SP140L\|57884\|AP | 0.238 | 0.470 | 0.000 | 0.232 |
| SP140L\|57885\|AP | 0.762 | 0.530 | 0.000 | -0.232 |
| COPS7B\|57936\|AP | 0.409 | 0.615 | 0.000 | 0.206 |
| COPS7B\|57937\|AP | 0.591 | 0.355 | 0.000 | -0.236 |
| COPS7B\|57959\|ES | 0.351 | 0.201 | 0.047 | -0.150 |
| FARP2\|58380\|AT | 0.222 | 0.444 | 0.000 | 0.222 |
| FARP2\|58381\|AT | 0.778 | 0.556 | 0.000 | -0.222 |
| THAP4\|58391\|AP | 0.155 | 0.559 | 0.000 | 0.403 |
| THAP4\|58392\|AP | 0.845 | 0.442 | 0.000 | -0.403 |
| TRIB3\|58448\|AP | 0.760 | 0.882 | 0.000 | 0.122 |
| TRIB3\|58449\|AP | 0.240 | 0.118 | 0.000 | -0.122 |
| SNX5\|58751\|RI | 0.784 | 0.518 | 0.000 | -0.265 |
| HM13\|58889\|AD | 0.478 | 0.585 | 0.001 | 0.107 |
| HM13\|58893\|ES | 0.806 | 0.680 | 0.000 | -0.126 |
| ACSS2\|59036\|AP | 0.640 | 0.320 | 0.000 | -0.321 |
| ACSS2\|59037\|AP | 0.360 | 0.668 | 0.000 | 0.308 |
| EDEM2\|59063\|AP | 0.706 | 0.813 | 0.000 | 0.108 |
| EDEM2\|59064\|AP | 0.293 | 0.185 | 0.000 | -0.109 |
| RBM39\|59248\|ME | 0.853 | 0.965 | 0.010 | 0.112 |
| SNX21\|59618\|ES | 0.640 | 0.492 | 0.000 | -0.148 |
| CTCFL\|59903\|AT | 0.210 | 0.063 | 0.033 | -0.147 |
| RPS21\|60075\|AA | 0.435 | 0.543 | 0.002 | 0.108 |
| ZGPAT\|60161\|AA | 0.822 | 0.290 | 0.000 | -0.532 |
| ZGPAT\|60162\|ES | 0.977 | 0.830 | 0.000 | -0.147 |
| RCAN1\|60488\|AP | 0.037 | 0.172 | 0.000 | 0.135 |
| RCAN1\|60491\|AP | 0.159 | 0.278 | 0.000 | 0.119 |
| RCAN1\|60494\|AP | 0.799 | 0.467 | 0.000 | -0.332 |
| PRDM15\|60682\|RI | 1.000 | 0.887 | 0.024 | -0.113 |
| TMPRSS3\|60707\|AT | 0.915 | 0.789 | 0.000 | -0.126 |
| TMPRSS3\|60708\|AT | 0.085 | 0.211 | 0.000 | 0.126 |

Supplementary Table 3. Univariate Cox regression analysis of survival alternative splicing events (SASEs) in TCGA-LIHC dataset.

| **ASE** | **HR** | **HR.95L** | **HR.95H** | **pValue** |
| --- | --- | --- | --- | --- |
| MORN1\|253\|AT | 0.375 | 0.204 | 0.691 | 0.002 |
| HES2\|400\|AT | 6.642 | 1.455 | 30.308 | 0.015 |
| HES2\|401\|AT | 0.151 | 0.033 | 0.687 | 0.015 |
| CLCN6\|686\|AT | 5.697 | 1.140 | 28.466 | 0.034 |
| MTFR1L\|1211\|ME | 0.100 | 0.032 | 0.311 | 0.000 |
| CSMD2\|1687\|AT | 0.471 | 0.236 | 0.941 | 0.033 |
| CSMD2\|1689\|AT | 2.605 | 1.266 | 5.359 | 0.009 |
| CLSPN\|1730\|AT | 7.208 | 2.850 | 18.233 | 0.000 |
| CLSPN\|1731\|AT | 0.139 | 0.055 | 0.351 | 0.000 |
| PPCS\|2075\|AD | 7.481 | 1.351 | 41.407 | 0.021 |
| EBNA1BP2\|2145\|AP | 3.500 | 1.351 | 9.069 | 0.010 |
| EBNA1BP2\|2146\|AP | 0.293 | 0.113 | 0.759 | 0.011 |
| ECHDC2\|3024\|ES | 0.141 | 0.040 | 0.497 | 0.002 |
| SCP2\|3045\|ES | 2.367 | 1.297 | 4.321 | 0.005 |
| PTGER3\|3416\|AT | 2.256 | 1.069 | 4.761 | 0.033 |
| GBP2\|3712\|AP | 0.485 | 0.238 | 0.989 | 0.047 |
| GBP2\|3713\|AP | 2.071 | 1.017 | 4.219 | 0.045 |
| GDAP2\|4377\|AT | 0.143 | 0.029 | 0.718 | 0.018 |
| GDAP2\|4378\|AT | 6.969 | 1.392 | 34.891 | 0.018 |
| RNF115\|7297\|ES | 0.065 | 0.009 | 0.474 | 0.007 |
| UBAP2L\|7814\|AT | 0.116 | 0.019 | 0.710 | 0.020 |
| ZBTB7B\|7878\|ES | 0.156 | 0.039 | 0.624 | 0.009 |
| NR1I3\|8628\|RI | 2.653 | 1.153 | 6.102 | 0.022 |
| UAP1\|8751\|ES | 0.401 | 0.176 | 0.913 | 0.030 |
| DCAF6\|8887\|ES | 0.098 | 0.016 | 0.606 | 0.012 |
| ASPM\|9286\|ES | 0.341 | 0.136 | 0.854 | 0.022 |
| ETNK2\|9466\|AP | 0.136 | 0.035 | 0.523 | 0.004 |
| ETNK2\|9467\|AP | 7.254 | 1.936 | 27.185 | 0.003 |
| FAM72A\|9575\|AP | 0.489 | 0.239 | 0.998 | 0.049 |
| FAM72A\|9577\|AT | 0.227 | 0.075 | 0.683 | 0.008 |
| FAM72A\|9578\|AT | 4.355 | 1.513 | 12.535 | 0.006 |
| NEK2\|9717\|AT | 9.215 | 2.527 | 33.606 | 0.001 |
| NEK2\|9718\|AT | 0.109 | 0.030 | 0.396 | 0.001 |
| EPHX1\|9997\|AP | 2.568 | 1.005 | 6.564 | 0.049 |
| EPHX1\|9998\|AP | 0.389 | 0.152 | 0.995 | 0.049 |
| SNAP47\|10055\|AP | 0.110 | 0.029 | 0.419 | 0.001 |
| SNAP47\|10056\|AP | 9.955 | 2.576 | 38.465 | 0.001 |
| MRPL55\|10116\|ES | 0.325 | 0.120 | 0.876 | 0.026 |
| MRPL55\|10120\|ES | 0.166 | 0.047 | 0.584 | 0.005 |
| MRPL55\|10127\|ES | 0.193 | 0.068 | 0.550 | 0.002 |
| MRPL55\|10146\|ES | 0.358 | 0.148 | 0.861 | 0.022 |
| COA6\|10335\|AP | 27.500 | 1.783 | 424.065 | 0.018 |
| COA6\|10336\|AP | 0.036 | 0.002 | 0.561 | 0.018 |
| EFCAB2\|10479\|AT | 3.183 | 1.053 | 9.620 | 0.040 |
| EFCAB2\|10480\|AT | 0.314 | 0.104 | 0.949 | 0.040 |
| RTKN2\|11869\|AT | 3.525 | 1.160 | 10.715 | 0.026 |
| RTKN2\|11871\|AT | 0.342 | 0.144 | 0.810 | 0.015 |
| MICU1\|12093\|AP | 4.750 | 2.352 | 9.593 | 0.000 |
| MICU1\|12094\|AP | 0.208 | 0.103 | 0.423 | 0.000 |
| ADK\|12257\|AP | 0.301 | 0.098 | 0.927 | 0.036 |
| ADK\|12258\|AP | 3.540 | 1.160 | 10.809 | 0.026 |
| PAPSS2\|12457\|ES | 0.311 | 0.134 | 0.721 | 0.007 |
| PANK1\|12493\|AP | 2.721 | 1.160 | 6.380 | 0.021 |
| PANK1\|12494\|AP | 0.374 | 0.158 | 0.884 | 0.025 |
| ZDHHC16\|12704\|ES | 0.103 | 0.021 | 0.503 | 0.005 |
| LDB1\|12934\|AP | 5.766 | 2.030 | 16.375 | 0.001 |
| LDB1\|12935\|AP | 0.146 | 0.052 | 0.411 | 0.000 |
| ACSL5\|13108\|AP | 2.349 | 1.185 | 4.659 | 0.014 |
| ACSL5\|13111\|AP | 0.431 | 0.218 | 0.853 | 0.016 |
| CHID1\|13801\|AP | 0.077 | 0.008 | 0.742 | 0.027 |
| CHID1\|13803\|AP | 0.324 | 0.146 | 0.718 | 0.005 |
| CHID1\|13804\|AP | 2.293 | 1.189 | 4.424 | 0.013 |
| IGF2\|13899\|AP | 0.546 | 0.301 | 0.990 | 0.046 |
| APIP\|14965\|AT | 9055.181 | 5.455 | 15030971.581 | 0.016 |
| APIP\|14967\|AT | 0.000 | 0.000 | 0.183 | 0.016 |
| DGKZ\|15540\|AP | 9.172 | 2.966 | 28.361 | 0.000 |
| DGKZ\|15541\|AP | 0.164 | 0.048 | 0.562 | 0.004 |
| SLC43A1\|15844\|AP | 4.848 | 1.186 | 19.813 | 0.028 |
| SLC3A2\|16462\|AP | 4.644 | 1.237 | 17.439 | 0.023 |
| SLC3A2\|16464\|AP | 0.146 | 0.041 | 0.520 | 0.003 |
| VEGFB\|16601\|AA | 55.793 | 5.439 | 572.363 | 0.001 |
| BAD\|16616\|RI | 0.021 | 0.001 | 0.710 | 0.031 |
| LTBP3\|16865\|ES | 0.082 | 0.026 | 0.261 | 0.000 |
| NUMA1\|17515\|ES | 0.248 | 0.068 | 0.897 | 0.034 |
| SLCO2B1\|17818\|AP | 2.973 | 1.466 | 6.030 | 0.003 |
| SLCO2B1\|17819\|AP | 0.359 | 0.177 | 0.731 | 0.005 |
| SMCO4\|18299\|AP | 3.621 | 1.605 | 8.170 | 0.002 |
| SMCO4\|18300\|AP | 0.279 | 0.124 | 0.629 | 0.002 |
| C11orf1\|18686\|AP | 0.326 | 0.113 | 0.939 | 0.038 |
| C11orf1\|18688\|AP | 2.983 | 1.013 | 8.786 | 0.047 |
| APOA1\|18870\|RI | 5.125 | 1.531 | 17.159 | 0.008 |
| VWA5A\|19211\|AT | 5.548 | 1.825 | 16.869 | 0.003 |
| VWA5A\|19212\|AT | 0.180 | 0.059 | 0.547 | 0.002 |
| TBRG1\|19224\|ES | 0.252 | 0.076 | 0.842 | 0.025 |
| TBRG1\|19225\|ES | 0.177 | 0.055 | 0.572 | 0.004 |
| CHEK1\|19311\|AT | 9.458 | 2.350 | 38.063 | 0.002 |
| CHEK1\|19312\|AT | 0.106 | 0.026 | 0.426 | 0.002 |
| TROAP\|21551\|AT | 0.057 | 0.016 | 0.201 | 0.000 |
| TROAP\|21552\|AT | 5.329 | 1.848 | 15.365 | 0.002 |
| TROAP\|21553\|AT | 5.774 | 1.326 | 25.136 | 0.019 |
| RACGAP1\|21625\|AT | 0.120 | 0.021 | 0.671 | 0.016 |
| RACGAP1\|21628\|AT | 8.348 | 1.490 | 46.778 | 0.016 |
| SUOX\|22340\|ES | 7.843 | 2.317 | 26.545 | 0.001 |
| UNG\|24277\|AP | 4.619 | 1.627 | 13.117 | 0.004 |
| UNG\|24278\|AP | 0.219 | 0.077 | 0.621 | 0.004 |
| VPS29\|24446\|ES | 8.087 | 2.450 | 26.689 | 0.001 |
| PRKAB1\|24709\|ES | 0.326 | 0.138 | 0.768 | 0.010 |
| OGFOD2\|25005\|AP | 8.974 | 2.332 | 34.530 | 0.001 |
| OGFOD2\|25006\|AP | 0.111 | 0.029 | 0.429 | 0.001 |
| UGGT2\|26129\|AT | 3.990 | 1.125 | 14.150 | 0.032 |
| UGGT2\|26131\|AT | 0.237 | 0.060 | 0.933 | 0.039 |
| NALCN\|26204\|AT | 2.185 | 1.050 | 4.543 | 0.036 |
| CARKD\|26250\|AP | 0.093 | 0.029 | 0.298 | 0.000 |
| CARKD\|26251\|AP | 7.857 | 2.633 | 23.448 | 0.000 |
| TMEM55B\|26455\|AD | 5.699 | 1.489 | 21.815 | 0.011 |
| MRPL52\|26638\|ES | 5.379 | 1.819 | 15.904 | 0.002 |
| MRPL52\|26642\|RI | 8.762 | 3.472 | 22.113 | 0.000 |
| ACIN1\|26708\|ES | 0.186 | 0.057 | 0.609 | 0.005 |
| DCAF11\|26830\|AP | 0.320 | 0.133 | 0.766 | 0.011 |
| DCAF11\|26831\|AP | 3.188 | 1.344 | 7.565 | 0.009 |
| KTN1\|27637\|ES | 14.694 | 3.863 | 55.892 | 0.000 |
| GSTZ1\|28583\|AP | 0.353 | 0.147 | 0.850 | 0.020 |
| GSTZ1\|28584\|AP | 2.677 | 1.110 | 6.457 | 0.028 |
| XRCC3\|29495\|AT | 0.005 | 0.000 | 0.090 | 0.000 |
| XRCC3\|29496\|AT | 185.210 | 11.071 | 3098.411 | 0.000 |
| C14orf2\|29533\|ES | 6.778 | 2.500 | 18.373 | 0.000 |
| ASPG\|29539\|ES | 0.094 | 0.027 | 0.321 | 0.000 |
| C14orf80\|29661\|ES | 0.221 | 0.073 | 0.666 | 0.007 |
| C14orf80\|29662\|ES | 0.310 | 0.136 | 0.709 | 0.005 |
| DUT\|30485\|AP | 3.945 | 1.152 | 13.507 | 0.029 |
| SMAD6\|31294\|AP | 6.648 | 2.620 | 16.870 | 0.000 |
| SMAD6\|31295\|AP | 0.146 | 0.057 | 0.370 | 0.000 |
| ACAN\|32395\|AT | 1.850 | 1.012 | 3.384 | 0.046 |
| ACAN\|32396\|AT | 0.541 | 0.296 | 0.989 | 0.046 |
| LRRC28\|32712\|AD | 0.041 | 0.002 | 0.814 | 0.036 |
| C16orf13\|32923\|ES | 0.187 | 0.078 | 0.446 | 0.000 |
| MLST8\|33211\|AP | 16.803 | 3.858 | 73.188 | 0.000 |
| MLST8\|33212\|AP | 0.061 | 0.014 | 0.263 | 0.000 |
| PKMYT1\|33330\|AA | 0.213 | 0.052 | 0.875 | 0.032 |
| NAA60\|33527\|RI | 0.080 | 0.028 | 0.229 | 0.000 |
| ABCC6\|34219\|AT | 3.047 | 1.016 | 9.135 | 0.047 |
| ABCC6\|34220\|AT | 0.328 | 0.109 | 0.984 | 0.047 |
| ACSM2B\|34361\|ES | 4.370 | 1.768 | 10.803 | 0.001 |
| SULT1A2\|35791\|ES | 0.315 | 0.133 | 0.749 | 0.009 |
| SULT1A2\|35810\|ES | 0.235 | 0.087 | 0.632 | 0.004 |
| INO80E\|36009\|ES | 0.274 | 0.130 | 0.579 | 0.001 |
| MTSS1L\|37410\|ES | 0.256 | 0.081 | 0.806 | 0.020 |
| CMC2\|37721\|ES | 0.289 | 0.120 | 0.699 | 0.006 |
| CMC2\|37726\|ES | 0.237 | 0.107 | 0.525 | 0.000 |
| CENPN\|37740\|AT | 2.367 | 1.003 | 5.589 | 0.049 |
| CDK10\|38115\|RI | 3.311 | 1.062 | 10.321 | 0.039 |
| ENO3\|38619\|AT | 3.382 | 1.242 | 9.205 | 0.017 |
| ENO3\|38620\|AT | 0.296 | 0.109 | 0.805 | 0.017 |
| ASGR2\|38835\|AA | 4.903 | 1.388 | 17.325 | 0.014 |
| CLDN7\|38892\|AP | 0.220 | 0.098 | 0.496 | 0.000 |
| CLDN7\|38894\|AP | 4.426 | 1.964 | 9.978 | 0.000 |
| TMEM107\|39128\|ES | 0.468 | 0.241 | 0.908 | 0.025 |
| MYO15A\|39574\|AP | 0.191 | 0.048 | 0.753 | 0.018 |
| AP2B1\|40319\|ES | 0.130 | 0.023 | 0.748 | 0.022 |
| CCL14\|40366\|AP | 4.307 | 1.400 | 13.252 | 0.011 |
| CCL14\|40367\|AP | 0.233 | 0.076 | 0.713 | 0.011 |
| MYO19\|40482\|AA | 0.239 | 0.073 | 0.788 | 0.019 |
| COASY\|41068\|RI | 5.875 | 1.756 | 19.661 | 0.004 |
| SLC25A39\|41837\|AA | 0.079 | 0.007 | 0.901 | 0.041 |
| SCRN2\|42120\|RI | 7.320 | 1.822 | 29.415 | 0.005 |
| HLF\|42576\|AP | 2.116 | 1.029 | 4.354 | 0.042 |
| HLF\|42579\|AP | 0.384 | 0.185 | 0.797 | 0.010 |
| BCAS3\|42864\|AP | 0.343 | 0.133 | 0.885 | 0.027 |
| BCAS3\|42865\|AP | 3.017 | 1.172 | 7.768 | 0.022 |
| CYGB\|43589\|AP | 0.393 | 0.201 | 0.770 | 0.007 |
| CYGB\|43591\|AP | 2.530 | 1.295 | 4.940 | 0.007 |
| SEC14L1\|43703\|AP | 6.299 | 1.680 | 23.619 | 0.006 |
| AFMID\|43794\|ES | 0.226 | 0.099 | 0.516 | 0.000 |
| AFMID\|43795\|ES | 0.215 | 0.103 | 0.447 | 0.000 |
| AFMID\|43796\|ES | 0.170 | 0.051 | 0.566 | 0.004 |
| AFMID\|43800\|ES | 0.091 | 0.033 | 0.251 | 0.000 |
| AFMID\|43801\|ES | 0.109 | 0.030 | 0.396 | 0.001 |
| AFMID\|43804\|ES | 0.211 | 0.094 | 0.474 | 0.000 |
| AFMID\|43811\|ES | 7.248 | 2.271 | 23.135 | 0.001 |
| AFMID\|43814\|ES | 0.327 | 0.173 | 0.619 | 0.001 |
| AFMID\|43824\|ES | 0.406 | 0.181 | 0.911 | 0.029 |
| AFMID\|43832\|ES | 0.271 | 0.135 | 0.541 | 0.000 |
| CANT1\|43973\|RI | 0.196 | 0.055 | 0.697 | 0.012 |
| BAIAP2\|44096\|ES | 0.286 | 0.118 | 0.694 | 0.006 |
| PCYT2\|44230\|ES | 0.194 | 0.062 | 0.606 | 0.005 |
| ENOSF1\|44462\|AT | 6.906 | 1.343 | 35.515 | 0.021 |
| ENOSF1\|44464\|AT | 0.145 | 0.028 | 0.745 | 0.021 |
| MYOM1\|44486\|ES | 2.960 | 1.166 | 7.514 | 0.022 |
| KIAA1468\|45699\|ME | 3.505 | 1.379 | 8.908 | 0.008 |
| PQLC1\|46257\|ES | 0.183 | 0.039 | 0.870 | 0.033 |
| TXNL4A\|46282\|ES | 0.334 | 0.139 | 0.802 | 0.014 |
| MUM1\|46457\|ES | 6.770 | 2.378 | 19.275 | 0.000 |
| INSR\|47099\|ES | 0.218 | 0.063 | 0.760 | 0.017 |
| AKAP8L\|48076\|ES | 0.067 | 0.017 | 0.258 | 0.000 |
| TPM4\|48124\|AP | 0.019 | 0.002 | 0.166 | 0.000 |
| TPM4\|48125\|AP | 50.043 | 5.914 | 423.468 | 0.000 |
| COPE\|48519\|ES | 0.290 | 0.135 | 0.622 | 0.001 |
| ZNF506\|48680\|AT | 0.069 | 0.011 | 0.441 | 0.005 |
| ZNF738\|48763\|AT | 0.342 | 0.132 | 0.885 | 0.027 |
| ZNF675\|48823\|AT | 0.281 | 0.085 | 0.930 | 0.038 |
| WDR62\|49337\|RI | 0.211 | 0.072 | 0.621 | 0.005 |
| WDR62\|49339\|RI | 0.317 | 0.135 | 0.743 | 0.008 |
| ZNF567\|49414\|AP | 2.930 | 1.584 | 5.420 | 0.001 |
| ZNF567\|49415\|AP | 0.263 | 0.128 | 0.538 | 0.000 |
| CEACAM1\|50162\|ES | 2.145 | 1.071 | 4.294 | 0.031 |
| OPA3\|50488\|AT | 0.076 | 0.009 | 0.653 | 0.019 |
| OPA3\|50489\|AT | 13.232 | 1.532 | 114.308 | 0.019 |
| BCAT2\|50810\|AP | 2.274 | 1.139 | 4.539 | 0.020 |
| BCAT2\|50811\|AP | 0.373 | 0.177 | 0.788 | 0.010 |
| BCAT2\|50816\|ES | 0.461 | 0.231 | 0.920 | 0.028 |
| IRF3\|51010\|ES | 0.217 | 0.085 | 0.555 | 0.001 |
| ISOC2\|52105\|ES | 0.161 | 0.078 | 0.329 | 0.000 |
| ZNF530\|52304\|ES | 0.412 | 0.198 | 0.858 | 0.018 |
| ITGB1BP1\|52617\|AA | 0.333 | 0.140 | 0.792 | 0.013 |
| KHK\|52929\|ME | 5.719 | 2.317 | 14.119 | 0.000 |
| PUS10\|53672\|AP | 0.312 | 0.110 | 0.880 | 0.028 |
| PUS10\|53673\|AP | 3.262 | 1.125 | 9.463 | 0.030 |
| UGP2\|53744\|AP | 4.271 | 1.753 | 10.406 | 0.001 |
| UGP2\|53745\|AP | 0.244 | 0.101 | 0.592 | 0.002 |
| CBWD2\|55054\|ES | 3.971 | 1.858 | 8.484 | 0.000 |
| BIN1\|55200\|ES | 2.862 | 1.019 | 8.037 | 0.046 |
| ITGA6\|55968\|ES | 0.214 | 0.062 | 0.738 | 0.015 |
| GULP1\|56499\|AT | 0.390 | 0.212 | 0.719 | 0.003 |
| GULP1\|56500\|AT | 2.570 | 1.395 | 4.732 | 0.002 |
| FN1\|57392\|AA | 33.003 | 2.043 | 533.087 | 0.014 |
| FN1\|57393\|AA | 3.713 | 1.330 | 10.360 | 0.012 |
| FN1\|57395\|ES | 29.195 | 2.533 | 336.443 | 0.007 |
| FN1\|57397\|ES | 4.477 | 1.909 | 10.500 | 0.001 |
| ATG9A\|57634\|AP | 0.094 | 0.020 | 0.448 | 0.003 |
| ATG9A\|57635\|AP | 9.182 | 1.947 | 43.293 | 0.005 |
| GMPPA\|57715\|AD | 5.008 | 1.331 | 18.843 | 0.017 |
| COPS7B\|57936\|AP | 3.858 | 1.350 | 11.025 | 0.012 |
| COPS7B\|57937\|AP | 0.224 | 0.077 | 0.649 | 0.006 |
| FARP2\|58380\|AT | 3.036 | 1.225 | 7.527 | 0.016 |
| FARP2\|58381\|AT | 0.328 | 0.132 | 0.813 | 0.016 |
| SNX5\|58751\|RI | 0.196 | 0.066 | 0.582 | 0.003 |
| HM13\|58889\|AD | 18.547 | 4.505 | 76.350 | 0.000 |
| HM13\|58893\|ES | 0.276 | 0.125 | 0.607 | 0.001 |
| RBM39\|59248\|ME | 6.043 | 1.144 | 31.930 | 0.034 |
| ZGPAT\|60161\|AA | 0.282 | 0.111 | 0.712 | 0.007 |
| ZGPAT\|60162\|ES | 0.219 | 0.060 | 0.801 | 0.022 |
| RCAN1\|60494\|AP | 0.448 | 0.209 | 0.960 | 0.039 |
| TMPRSS3\|60707\|AT | 0.301 | 0.111 | 0.815 | 0.018 |
| TMPRSS3\|60708\|AT | 3.333 | 1.233 | 9.015 | 0.018 |
| SLC19A1\|60877\|AP | 0.355 | 0.170 | 0.741 | 0.006 |
| SLC19A1\|60878\|AP | 3.443 | 1.683 | 7.043 | 0.001 |
| NF2\|61626\|ES | 0.298 | 0.111 | 0.800 | 0.016 |
| FBXO7\|61930\|AP | 5.100 | 1.657 | 15.695 | 0.005 |
| FBXO7\|61931\|AP | 0.208 | 0.067 | 0.641 | 0.006 |
| TCF20\|62501\|ES | 0.218 | 0.083 | 0.577 | 0.002 |
| FBLN1\|62666\|AT | 0.198 | 0.065 | 0.607 | 0.005 |
| TRABD\|62789\|AP | 0.286 | 0.085 | 0.966 | 0.044 |
| TRABD\|62790\|AP | 3.574 | 1.101 | 11.603 | 0.034 |
| OGG1\|63164\|RI | 4.684 | 1.224 | 17.920 | 0.024 |
| FANCD2\|63304\|AT | 0.005 | 0.000 | 0.112 | 0.001 |
| FANCD2\|63307\|AT | 43.073 | 5.994 | 309.549 | 0.000 |
| SGOL1\|63696\|AT | 0.112 | 0.015 | 0.835 | 0.033 |
| SGOL1\|63698\|AT | 8.915 | 1.198 | 66.334 | 0.033 |
| ACAA1\|64017\|AD | 0.277 | 0.099 | 0.776 | 0.015 |
| PCBP4\|65125\|AA | 100.934 | 4.060 | 2509.061 | 0.005 |
| RPP14\|65434\|AD | 0.292 | 0.108 | 0.790 | 0.015 |
| FILIP1L\|65811\|AT | 0.346 | 0.129 | 0.931 | 0.036 |
| FILIP1L\|65812\|AT | 2.876 | 1.070 | 7.734 | 0.036 |
| GRAMD1C\|66220\|AP | 2.030 | 1.126 | 3.661 | 0.019 |
| GRAMD1C\|66223\|AP | 0.377 | 0.205 | 0.696 | 0.002 |
| MASP1\|68073\|AT | 3.670 | 1.560 | 8.636 | 0.003 |
| MASP1\|68075\|AT | 0.217 | 0.103 | 0.457 | 0.000 |
| CCDC50\|68126\|ES | 0.298 | 0.103 | 0.867 | 0.026 |
| OPA1\|68139\|ES | 0.264 | 0.072 | 0.971 | 0.045 |
| ABLIM2\|68744\|AT | 0.186 | 0.079 | 0.437 | 0.000 |
| ABLIM2\|68745\|AT | 5.314 | 2.265 | 12.468 | 0.000 |
| SEPSECS\|68957\|ES | 2.539 | 1.109 | 5.813 | 0.027 |
| SLAIN2\|69214\|ES | 0.044 | 0.011 | 0.175 | 0.000 |
| OCIAD1\|69246\|ES | 3.110 | 1.132 | 8.541 | 0.028 |
| FIP1L1\|69312\|ES | 0.094 | 0.018 | 0.478 | 0.004 |
| FIP1L1\|69315\|ES | 0.078 | 0.020 | 0.299 | 0.000 |
| RUFY3\|69446\|AP | 0.346 | 0.128 | 0.935 | 0.036 |
| USO1\|69551\|ES | 0.339 | 0.157 | 0.728 | 0.006 |
| NUDT6\|70521\|AP | 0.189 | 0.089 | 0.402 | 0.000 |
| NUDT6\|70523\|AP | 4.838 | 2.255 | 10.383 | 0.000 |
| NEIL3\|71226\|AT | 0.144 | 0.068 | 0.306 | 0.000 |
| NEIL3\|71227\|AT | 6.891 | 3.240 | 14.658 | 0.000 |
| ING2\|71271\|AP | 4.477 | 1.490 | 13.453 | 0.008 |
| ING2\|71272\|AP | 0.215 | 0.071 | 0.646 | 0.006 |
| ACSL1\|71316\|AP | 0.314 | 0.111 | 0.889 | 0.029 |
| ACSL1\|71319\|AP | 5.244 | 1.652 | 16.652 | 0.005 |
| SEPP1\|71907\|ES | 0.212 | 0.090 | 0.496 | 0.000 |
| CENPK\|72212\|AT | 5.049 | 1.285 | 19.843 | 0.020 |
| CENPK\|72213\|AT | 0.197 | 0.050 | 0.774 | 0.020 |
| POLR3G\|72767\|AT | 3.419 | 1.224 | 9.552 | 0.019 |
| POLR3G\|72768\|AT | 0.292 | 0.105 | 0.817 | 0.019 |
| ABLIM3\|74015\|AP | 0.269 | 0.086 | 0.841 | 0.024 |
| ABLIM3\|74017\|AP | 3.451 | 1.088 | 10.953 | 0.036 |
| ZNF346\|74698\|AT | 34.488 | 3.082 | 385.952 | 0.004 |
| ZNF346\|74699\|AT | 0.029 | 0.003 | 0.325 | 0.004 |
| SIRT5\|75389\|AP | 3.718 | 1.113 | 12.418 | 0.033 |
| SIRT5\|75390\|AP | 0.257 | 0.077 | 0.858 | 0.027 |
| SLC17A4\|75550\|AD | 2.670 | 1.274 | 5.595 | 0.009 |
| MTCH1\|76000\|AD | 18.627 | 2.972 | 116.735 | 0.002 |
| CCND3\|76154\|AP | 4.796 | 2.054 | 11.197 | 0.000 |
| CCND3\|76156\|AP | 0.280 | 0.120 | 0.650 | 0.003 |
| GPR116\|76429\|ES | 4.367 | 1.183 | 16.118 | 0.027 |
| STEAP4\|80361\|AT | 0.187 | 0.070 | 0.499 | 0.001 |
| STEAP4\|80362\|AT | 5.339 | 2.005 | 14.216 | 0.001 |
| MCM7\|80880\|AP | 0.219 | 0.062 | 0.766 | 0.017 |
| MCM7\|80881\|AP | 4.509 | 1.295 | 15.704 | 0.018 |
| RASA4\|81120\|AP | 4.475 | 1.515 | 13.216 | 0.007 |
| RASA4\|81121\|AP | 0.410 | 0.174 | 0.965 | 0.041 |
| STRIP2\|81747\|AT | 24.809 | 3.216 | 191.378 | 0.002 |
| STRIP2\|81748\|AT | 0.040 | 0.005 | 0.311 | 0.002 |
| C7orf49\|81873\|AD | 0.321 | 0.154 | 0.670 | 0.002 |
| MTUS1\|82818\|AP | 0.414 | 0.198 | 0.866 | 0.019 |
| FGL1\|82824\|AP | 3.815 | 1.567 | 9.287 | 0.003 |
| TNFRSF10C\|83064\|AT | 0.497 | 0.263 | 0.939 | 0.031 |
| TNFRSF10C\|83065\|AT | 2.009 | 1.064 | 3.793 | 0.032 |
| ADRA1A\|83135\|AT | 2.530 | 1.379 | 4.643 | 0.003 |
| ADRA1A\|83140\|AT | 0.201 | 0.081 | 0.497 | 0.001 |
| ERLIN2\|83343\|AP | 0.101 | 0.017 | 0.588 | 0.011 |
| PPAPDC1B\|83387\|AT | 5.034 | 1.314 | 19.281 | 0.018 |
| PPAPDC1B\|83388\|AT | 0.213 | 0.053 | 0.861 | 0.030 |
| TACC1\|83437\|AP | 0.268 | 0.126 | 0.570 | 0.001 |
| GINS4\|83515\|AT | 3.668 | 1.150 | 11.702 | 0.028 |
| GINS4\|83516\|AT | 0.273 | 0.085 | 0.870 | 0.028 |
| SLC20A2\|83727\|AP | 3.276 | 1.379 | 7.785 | 0.007 |
| ADHFE1\|84004\|AA | 2.572 | 1.098 | 6.023 | 0.030 |
| STAU2\|84157\|AT | 2.795 | 1.082 | 7.221 | 0.034 |
| ZFAND1\|84309\|ES | 0.257 | 0.088 | 0.755 | 0.013 |
| COX6C\|84676\|AT | 5.397 | 1.747 | 16.674 | 0.003 |
| COX6C\|84681\|AT | 0.275 | 0.095 | 0.801 | 0.018 |
| RNF19A\|84703\|AP | 0.358 | 0.130 | 0.985 | 0.047 |
| OXR1\|84847\|AP | 0.193 | 0.053 | 0.705 | 0.013 |
| TATDN1\|85085\|AD | 6.582 | 1.074 | 40.345 | 0.042 |
| ZFP41\|85399\|AT | 0.133 | 0.024 | 0.724 | 0.020 |
| SCRIB\|85500\|ES | 0.151 | 0.040 | 0.566 | 0.005 |
| ADCK5\|85590\|RI | 3.676 | 1.227 | 11.017 | 0.020 |
| CBWD1\|85690\|ES | 3.792 | 1.728 | 8.321 | 0.001 |
| CBWD1\|85692\|RI | 2.319 | 1.010 | 5.323 | 0.047 |
| SPATA6L\|85752\|AT | 2.907 | 1.143 | 7.394 | 0.025 |
| RCL1\|85779\|AP | 0.217 | 0.112 | 0.418 | 0.000 |
| RCL1\|85780\|AP | 4.717 | 2.451 | 9.081 | 0.000 |
| CNTLN\|85938\|AT | 0.087 | 0.012 | 0.630 | 0.016 |
| CNTLN\|85939\|AT | 7.744 | 1.170 | 51.251 | 0.034 |
| PLIN2\|85964\|RI | 0.248 | 0.084 | 0.728 | 0.011 |
| ARHGEF39\|86268\|AP | 0.383 | 0.167 | 0.878 | 0.023 |
| ARHGEF39\|86269\|AP | 2.610 | 1.140 | 5.977 | 0.023 |
| ARHGEF39\|86271\|AT | 0.248 | 0.070 | 0.883 | 0.031 |
| ARHGEF39\|86272\|AT | 4.034 | 1.133 | 14.368 | 0.031 |
| MELK\|86353\|ES | 0.417 | 0.250 | 0.696 | 0.001 |
| CBWD6\|86488\|AT | 0.273 | 0.079 | 0.939 | 0.039 |
| CBWD6\|86489\|AT | 3.445 | 1.005 | 11.816 | 0.049 |
| CBWD5\|86499\|AT | 5.347 | 1.256 | 22.763 | 0.023 |
| SLC44A1\|87112\|AT | 0.336 | 0.142 | 0.797 | 0.013 |
| SLC44A1\|87113\|AT | 2.975 | 1.254 | 7.058 | 0.013 |
| DNAJC25\|87223\|AT | 0.044 | 0.006 | 0.326 | 0.002 |
| DNAJC25\|87224\|AT | 22.823 | 3.069 | 169.733 | 0.002 |
| NEK6\|87526\|AP | 4.693 | 1.093 | 20.151 | 0.038 |
| TOR2A\|87657\|AD | 0.292 | 0.143 | 0.593 | 0.001 |
| CRAT\|87827\|ES | 0.135 | 0.020 | 0.923 | 0.041 |
| CRAT\|87828\|ES | 0.102 | 0.019 | 0.566 | 0.009 |
| SARDH\|88074\|AP | 3.368 | 1.511 | 7.508 | 0.003 |
| SARDH\|88075\|AP | 0.287 | 0.130 | 0.632 | 0.002 |
| EXD3\|88296\|AT | 5.153 | 2.255 | 11.776 | 0.000 |
| EXD3\|88298\|AT | 0.293 | 0.100 | 0.861 | 0.026 |
| RPS6KA3\|88669\|AP | 0.343 | 0.160 | 0.736 | 0.006 |
| PQBP1\|89029\|AD | 0.251 | 0.099 | 0.634 | 0.003 |
| KIF4A\|89372\|AT | 0.121 | 0.042 | 0.348 | 0.000 |
| KIF4A\|89373\|AT | 8.298 | 2.878 | 23.926 | 0.000 |
| UPF3B\|89980\|ES | 0.087 | 0.024 | 0.318 | 0.000 |
| LAMP2\|89999\|AT | 9.376 | 1.838 | 47.826 | 0.007 |
| DNASE1L1\|90581\|ES | 0.174 | 0.054 | 0.555 | 0.003 |
| TAZ\|90586\|ES | 0.118 | 0.029 | 0.477 | 0.003 |
| GLYATL1\|92586\|AP | 0.445 | 0.227 | 0.871 | 0.018 |
| GLYATL1\|92588\|AP | 2.121 | 1.084 | 4.152 | 0.028 |
| AFMID\|94690\|ES | 0.168 | 0.080 | 0.354 | 0.000 |
| AFMID\|94694\|ES | 0.081 | 0.027 | 0.247 | 0.000 |
| AFMID\|99319\|ES | 2.881 | 1.254 | 6.621 | 0.013 |
| APOC1\|99361\|ES | 0.123 | 0.051 | 0.298 | 0.000 |
| AFMID\|101499\|ES | 0.289 | 0.124 | 0.674 | 0.004 |
| SLC39A14\|140283\|ME | 0.118 | 0.048 | 0.286 | 0.000 |
| KHK\|206708\|ES | 0.246 | 0.111 | 0.544 | 0.001 |
| APOC3\|215383\|ES | 0.215 | 0.062 | 0.752 | 0.016 |
| HPX\|235159\|ES | 0.321 | 0.124 | 0.829 | 0.019 |
| HPX\|235160\|ES | 0.276 | 0.101 | 0.752 | 0.012 |
| ITIH3\|235314\|ES | 6.713 | 2.880 | 15.648 | 0.000 |
| APOC3\|235465\|ES | 3.344 | 1.303 | 8.583 | 0.012 |
| ITIH3\|235594\|ES | 0.159 | 0.060 | 0.419 | 0.000 |
| ECHDC2\|319385\|ES | 12.386 | 2.347 | 65.354 | 0.003 |

Supplementary Table 4. Summary of correlation coefficient for expressions of SF3B6 and SF3B6-related survival alternative splicing events (SF3B6-SASEs)

| **SF3B6-SASE** | **Correlation** | **P-value** |
| --- | --- | --- |
| FANCD2\|63307\|AT | 0.512 | 0.000 |
| FANCD2\|63304\|AT | -0.455 | 0.000 |
| KIF4A\|89372\|AT | -0.449 | 0.000 |
| KIF4A\|89373\|AT | 0.449 | 0.000 |
| NEK2\|9718\|AT | -0.443 | 0.000 |
| NEK2\|9717\|AT | 0.443 | 0.000 |
| RCL1\|85780\|AP | 0.434 | 0.000 |
| HM13\|58893\|ES | -0.431 | 0.000 |
| RCL1\|85779\|AP | -0.428 | 0.000 |
| KHK\|52929\|ME | 0.425 | 0.000 |
| RPS6KA3\|88669\|AP | -0.424 | 0.000 |
| UNG\|24278\|AP | -0.410 | 0.000 |
| SLC44A1\|87112\|AT | -0.406 | 0.000 |
| SLC44A1\|87113\|AT | 0.406 | 0.000 |
| TROAP\|21551\|AT | -0.405 | 0.000 |
| NEIL3\|71226\|AT | -0.403 | 0.000 |
| NEIL3\|71227\|AT | 0.403 | 0.000 |

Supplementary Table 5. Primers sequences of siRNAs for SF3B6

| **Gene** | **Primer** | **Sequence** |
| --- | --- | --- |
| si-NC | Sense | 5’-UUCUCCGAACGUGUCACGUTT-3’ |
|  | Anti-sense | 5’-ACGUGACACGUUCGGAGAATT-3' |
| si-SF3B6-1 | Sense | 5’-CCUGAAGUAAAUCGGAUAUTT-3' |
|  | Anti-sense | 5’-AUAUCCGAUUUACUUCAGGTT-3' |
| si-SF3B6-2 | Sense | 5’-GAAAUAUGGACCUAUUCGUTT-3' |
|  | Anti-sense | 5’-ACGAAUAGGUCCAUAUUUCTT-3' |
| si-SF3B6-3 | Sense | 5’-CGGGAUUCAAUGUUUGUAATT-3' |
|  | Anti-sense | 5’-UUACAAACAUUGAAUCCCGTT-3' |

Supplementary Table 6. Primers sequences used in qRT-PCR

| **Name** | **Forward primer** | **Reverse primer** |
| --- | --- | --- |
| β-ACTIN | 5’-CACCATTGGCAATGAGCGGTTCC-3’ | 5’-GTAGTTTCGTGGATGCCACAGG-3’ |
| SF3B6 | 5’-AGGGCGAACATTCGACTTCC-3’ | 5’-TTCAGGTGTGTTCCCCACTC-3’ |
